# In Silico Identification of Promising New Pyrazole Derivative-Based Small Molecules for Modulating CRMP2, C-RAF, CYP17, VEGFR, C-KIT, and HDAC—Application towards Cancer Therapeutics

**DOI:** 10.3390/cimb44110361

**Published:** 2022-10-31

**Authors:** Fatima Ezzahra Bennani, Khalid Karrouchi, Latifa Doudach, Mario Scrima, Noor Rahman, Luca Rastrelli, Trina Ekawati Tallei, Christopher E. Rudd, My El Abbes Faouzi, M’hammed Ansar

**Affiliations:** 1 Laboratory of Pharmacology and Toxicology, Bio Pharmaceutical and Toxicological Analysis Research Team, Faculty of Medicine and Pharmacy, Mohammed V University in Rabat, Rabat BP6203, Morocco; 2 Division of Immunology-Oncology, Centre de Recherche Hôpital Maisonneuve-Rosemont (CR-HMR), Montreal, QC H1T 2M4, Canada; 3 Laboratory of Analytical Chemistry, Faculty of Medicine and Pharmacy, Mohammed V University in Rabat, Rabat BP6203, Morocco; 4 Department of Biomedical Engineering Medical Physiology, Higher School of Technical Education of Rabat, Mohammed V University in Rabat, Rabat BP6203, Morocco; 5 Biogem Scarl, Via Camporeale, 83031 Ariano Irpino, AV, Italy; 6 Department of Biochemstry, Abdul Wali Khan University Mardan, Mardan 23200, Pakistan; 7 Dipartimento di Farmacia, University of Salerno, Via Giovanni Paolo II, 84084 Fisciano, SA, Italy; 8 Department of Biology, Faculty of Mathematics and Natural Sciences, Sam Ratulangi University, Manado 95115, North Sulawesi, Indonesia; 9 Department of Microbiology, Infection and Immunology, Faculty of Medicine, Université de Montreal, Montreal, QC H3T 1J4, Canada; 10 Division of Experimental Medicine, Department of Medicine, McGill University Health Center, McGill University, Montreal, QC H4A 3J1, Canada; 11 Laboratory of Medicinal Chemistry, Faculty of Medicine and Pharmacy, Mohammed V University in Rabat, Rabat BP6203, Morocco

**Keywords:** molecular docking, molecular dynamic simulation, pyrazole derivatives, cancer targets

## Abstract

Despite continual efforts being made with multiple clinical studies and deploying cutting-edge diagnostic tools and technologies, the discovery of new cancer therapies remains of severe worldwide concern. Multiple drug resistance has also emerged in several cancer cell types, leaving them unresponsive to the many cancer treatments. Such a condition always prompts the development of next-generation cancer therapies that have a better chance of inhibiting selective target macromolecules with less toxicity. Therefore, in the present study, extensive computational approaches were implemented combining molecular docking and dynamic simulation studies for identifying potent pyrazole-based inhibitors or modulators for CRMP2, C-RAF, CYP17, c-KIT, VEGFR, and HDAC proteins. All of these proteins are in some way linked to the development of numerous forms of cancer, including breast, liver, prostate, kidney, and stomach cancers. In order to identify potential compounds, 63 in-house synthesized pyrazole-derivative compounds were docked with each selected protein. In addition, single or multiple standard drug compounds of each protein were also considered for docking analyses and their results used for comparison purposes. Afterward, based on the binding affinity and interaction profile of pyrazole compounds of each protein, potentially strong compounds were filtered out and further subjected to 1000 ns MD simulation analyses. Analyzing parameters such as RMSD, RMSF, RoG and protein–ligand contact maps were derived from trajectories of simulated protein–ligand complexes. All these parameters turned out to be satisfactory and within the acceptable range to support the structural integrity and interaction stability of the protein–ligand complexes in dynamic state. Comprehensive computational analyses suggested that a few identified pyrazole compounds, such as M33, M36, M72, and M76, could be potential inhibitors or modulators for HDAC, C-RAF, CYP72 and VEGFR proteins, respectively. Another pyrazole compound, M74, turned out to be a very promising dual inhibitor/modulator for CRMP2 and c-KIT proteins. However, more extensive study may be required for further optimization of the selected chemical framework of pyrazole derivatives to yield improved inhibitory activity against each studied protein receptor.

## 1. Introduction

Every year, more than one million new cancer cases are diagnosed around the world [1]. Despite the continuing efforts being made to conduct numerous clinical trials and the use of cutting-edge diagnostic techniques and equipment, treating cancer remains a considerable issue for researchers [1,2]. Cancer treatment has long been regarded as one of the most important critical and clinical issues [2]. Cancer is currently the world’s second leading cause of death [3], and is expected to become the primary cause of mortality in the coming few years. Despite substantial progresses being made in the development of numerous anticancer medicines over the last few decades, treatment regimens still have some critical limitations in terms of selective anticancer drug targets [4], which ultimately leads to undesirable side effects. As a consequence, several cancer cell types developed multiple-drug resistance, rendering them impassive to the various available cancer treatments [5]. Depending on the nature and stage of the tumors, a variety of approaches have been developed for treating cancer, which include systemic therapy or chemoradiotherapy, adjuvant therapy, and gene therapy [2,6]. Over the last few decades with technological advancement and increasing knowledge of tumor biology research, the perspective of clinical oncology has shifted from “one size fits all” to “personalized cancer treatment” [6].

The current study intended to evaluate the efficacy of a set of pyrazole derivatives synthesized in-house [7,8,9,10,11,12,13,14,15,16,17,18] (63 structures that are displayed in Supplementary Information) against six important cancer targets: CRMP2, C-RAF, CYP17, c-KIT, VEGFR, and HDAC9.

The importance of these proteins is not only as anticancer drug targets but has also emerged in a number of allied health-related issues. These seven proteins—(CRMP2, C-RAF, CYP17, c-KIT+ VEGFR, and HDAC9)were considered in this in silico study owing to their association with several cancers: breast cancer [19], liver cancer [20], prostate cancer [21], kidney cancer (for both C-KIT [22] and VEGFR [23]), and stomach cancer [24]. To evaluate the possible binding mechanism of the 63 structures against the defined target, we compared the findings with their reference modulator (agonist or antagonist), which are nalidixic acid (demonstrated in a recent study to activate CRMP2 to treat metastatic breast cancer [1]), sorafenib (i.e., Nexavar^®^) (approved for the treatment of hepatocellular carcinoma, downstream intracellular serine/threonine kinases, especially the RAF kinases, and is more selective for C-Raf than the other isoforms of RAF kinase) [25,26,27], abiraterone acetate (i.e., Zytiga^®^, Yonsa^®^) (a promiscuous medicine, interacting with several targets that include CYP17A1 and a panel of hepatic CYP enzymes—it suppress the production of androgens and decreases the production of testosterone) [28,29,30,31,32], orteronel (reported as inhibitor of CYP 17,20-lyase in the androgen signaling pathway for metastatic castration-resistant prostate cancer) [33,34], galeterone (TOK-001, VN/124-1) (also inhibitor of CYP-17) [35], sunitinib (Sutent^®^) (modulates both C-KIT and VEGFR) [36,37,38], SAHA (recently reported as an inhibitor of the HDAC-9) [24], and TMP269 (described as an inhibitor across HDAC1–11 but remarkably selective for class IIa HDAC family) [39].

The CRMP2 (collapsin response mediator protein 2) is a phosphoprotein found in the cytosol that regulates cytoskeletal dynamics. Earlier studies have demonstrated that changes or alteration in CRMP2 expression have been linked to breast cancer progression [19,40,41], although the definitive underlying mechanism is still unknown. A study suggested that in TNBC tissues, the triple-phosphorylated CRMP2 increased level was observed in the C-terminal residues, particularly at the positions Thr509, Ser518, and Ser522 of CRMP2 [41].

Regarding the RAF (rapid accelerated fibrosarcoma) kinase isoforms, i.e., C-RAF protein was considered in the present study, which is involved in cell mitogenesis in response to growth factors, cytokines, and numerous oncogenes [42]. It is now known that all three mammalian RAF proteins, including C-RAF, can be stimulated by the RAS oncogene, which can exert both kinase-dependent and/or independent tumor-promoting functions. The MEK/ERK pathway, whose activation is linked to proliferation in a wide range of human malignancies, is primarily responsible for kinase-dependent actions [43]. C-RAF is a 74 kDa protein that is found in all adult tissues, but is most abundant in muscle, cerebellum, and fetal brain [44] and has been found to be highly expressed in liver cancer, [20,25,43,45]. Although mutational alterations in C-RAF protein are uncommon in human malignancies, i.e., approximately 1% (http://www.sanger.ac.uk/genetics/CGP/cosmic (accessed on 10 October 2022)), in androgen-independent prostate cancer, the amplification of C-RAF and other members of the ERK pathway has been significantly observed during hormone escape. Most importantly, both C-RAF amplifications (4%) and deletions (2.2%) were found to be strongly connected with tumor progression [46]. Therefore, targeting the C-RAF protein can be an interesting approach for therapeutic agent development against many cancer types, including liver cancer.

Another important enzyme studied in this article is the cytochrome 450 (CYP) superfamily, which is responsible for about 75% of total drug metabolism [47]. CYP enzymes mediate enzymatic conversion or activation of chemical or drug entities to produce specific essential compounds that can bind to macromolecules for further necessary action [48]. Likewise, many CYP subfamilies, CYP17α hydroxylase/17,20 lyase (CYP17), have been found to be associated with many cancer types, including prostate cancer. The CYP17 enzyme has an important role in androgen synthesis and its association is linked with prostate cancer development [21]. Since androgen signaling plays an important role and its association is found with prostate cancer proliferation and metastasis, androgen deprivation therapy (ADT) or castration therapy is the treatment of choice for newly diagnosed metastatic prostate cancer. Although the FDA-approved drug abiraterone acetate used as the CYP17 inhibitor for the treatment of metastatic prostate cancer [32,49], still there is much ongoing research on prostate cancer towards development of newer anticancer drug entities against this important drug target. Moreover, several studies have reported higher levels of CYP17 expression in prostate cancer [50,51,52].

Kidney cancer or renal cell carcinoma is one of the most frequent cancer types, accounting for approximately 3% of total reported human cancers in the world [53]. Over the years, understanding of the biological background of c-KIT in renal cell carcinoma has been gained, particularly the overexpression of c-Kit, which has been observed to play a role in tumor growth and progression, making it an appealing therapeutic target for kidney cancer therapeutic development [53,54,55]. C-KIT is expressed or found in many tumor types derived from melanocytes, germ cells, hematopoietic stem cells, mast cells, and Cajal interstitial cells, as well as other cancers, such as chromophobe renal cell carcinoma [22]. Not only the c-KIT protein but also another tyrosine kinase family receptor, i.e., vascular endothelial growth factor receptor (VEGFR), has also involved in progression of cancer development, mainly due to mutation or upregulation/overexpression of VEGFR [56,57,58]. Prognostic role of both proteins was found to be associated with aggressive disease progression in renal cell carcinoma. Therefore, evaluating diagnostic usefulness as a biomarker, targeting the c-KIT and VEGFR pathways can possibly provide a potentially fit targeted tumor therapeutic modality against kidney cancer or renal cell carcinoma. Beside kidney cancer, stomach cancer also occurs frequently and is the third leading cause of cancer deaths and fifth in terms of cancer cases globally [59].

The last target studied was HDAC7. HDACs are known to have many key roles in the regulation of genes involved in the genesis and progression of variety of cancers, including stomach cancer [60]. The 18 HDACs identified so far are broadly categorized into four HDAC classes (I–IV). HDAC7, a class II HDAC, was found to be involved in cancer development and progression [61,62]. This class II HDAC family member was also found to be associated with several numbers of transcription factors and corepressors, such as KLF4, STAT3, MEF2A/C/D, HIF-1α, RUNX2, ERα, FOXA1, FOXP3, and (NCOR1) [63,64,65]. Earlier studies have revealed strong and significant correlations with aberrant expression of HDAC7 and progression in breast, lung, ovarian, and gastric cancer, glioma, nasopharyngeal carcinoma, and hemolymphatic tumors [66,67,68,69,70,71,72,73,74]. In both normal and pathological conditions, HDAC7 affects cell proliferation, differentiation, migration, apoptosis, and stemness [68,75]. One of the important FDA-approved drug targets, HDAC7 also could offer novel therapeutic application to explore the underlying mechanism of cancer progression and perturbation of its ligands systems for development of better or optimized drug candidates. Based on substantial research findings, it is now obvious that any dysregulated protein activities are directly associated with a wide spectrum of human disorders, including malignancies. As a result, the inhibition of those proteins has become an attractive area in drug-development studies. Concomitantly, the present research goal was to identify pyrazole derivative-based small molecules that were synthesized in our laboratory that can modulate or inhibit the action of six selected proteins/receptors, and possibly can trigger a halt or restoration of dysregulated protein functions.

A number of studies have demonstrated the therapeutic voyage of pyrazole derivatives as potential anticancer agents [76,77,78,79,80,81,82,83,84]. The progression of exploring pyrazole derivative-based anticancer agents is still a highly demanding area of research for developing therapeutic interventions against several different types of cancers. In the last few decades, as per extensive studies on pyrazole derivatives, it is now proven that the pyrazole nucleus has broader spectra of biological roles in many areas, including medicine to agrochemicals and other fields, such as materials and dyes, etc. Moreover, some pyrazole moieties, such as aminopyrine or amino phenazone, phenylbutazone, sulfinpyrazone phenazone, and metamizole or dipyrone, are the main constituents of commercially available therapeutic agents. As a result, pyrazole chemistry continues to grow people’s interest for therapeutic exploration. Throughout the last few decades, the use of pyrazole derivatives in the development of anticancer agents has acquired a lot of attention in research communities. In addition to the anticancer activity implicated by numbers of pyrazole derivatives, various other biological properties or activities such as antihypertensive, antimicrobial, anticonvulsant, anti-HIV, antifungal, and anti-inflammatory properties are possessed by this important chemical scaffold constituting of heterocyclic five member rings linked with two nitrogen atoms. From a similar perspective, the current research goal was to evaluate and identify pyrazole compounds for modulation or inhibitory function of six selected proteins that have been linked to a variety of pathological disorders, including several malignancies, and thus could help to rethink unmet cancer therapeutic problems.

In silico based studies have been conducted for evaluating the efficacy of several pyrazole derivatives for assessing different types of biological properties, including anticancer activity [85,86,87,88,89,90,91,92,93,94,95]. In the present study, using molecular docking and molecular dynamic (MD) simulation, a total of 63 pyrazole derivatives were computationally investigated for assessing the modulatory or inhibitory effects against the cited protein targets linked to diverse cancer types. In silico docking of all 63 pyrazole derivatives was performed initially for all six protein targets and the best interacting protein–pyrazole complexes were further subjected to 1000 ns MD studies for evaluating interaction stability. The results were thoroughly examined and based on binding interaction profile and acceptable MD simulation characteristics, a few pyrazole compounds, such as M33, M36, M72, and M76, were identified to have the most potential as inhibitors/modulators for the HDAC, C-RAF, CYP72, and VEGFR proteins, respectively. Another compound, M74, was also found to be a dual inhibitor of two proteins: CRMP2 and c-Kit. The appearance of intermolecular interactions, such as hydrogen bonds (H-bonds) or hydrophobic contacts between pyrazole derivatives and the six protein targets, suggest that the identified pyrazole derivatives can be used as drug-like small molecules for exhibiting essential pharmacological action against many cancer types, though this may also need further experimental validation for better understanding the inhibitory mechanism analyses.

## 2. Materials and Methods

With the advancement of modern computational technology, it has become possible to simulate large macromolecular complex systems and understand the dynamic nature of binding events for any specified time period. This was used in the present study, although conventional but extensive computational techniques, such as molecular docking and dynamic simulation studies and ADMET estimation, were implemented toward identification of potential pyrazole derivatives against a few attractive drug targets: CRMP2, C-RAF, CYP17, c-KIT, VEGFR, and HDAC proteins.

### 2.1. Molecular Docking

#### 2.1.1. Ligand (Pyrazole Derivatives) Preparation

All 63 pyrazole derivatives were drawn using the ChemDraw module of ChemBioOffice software suite [96] They were then checked for any structural or bonding errors. The 2D structures of all 63 pyrazole derivatives are displayed in the Supplementary Information. All molecules were saved in SMILES, mol2 and .sdf format for further use in different tools. Using the AutoDock Tool 1.5.6 (ADT), all compounds were prepared with addition of hydrogen atoms, assigning the atoms to AD4 type and adjustment of the charges. Finally, all compounds were saved into pdbqt file format, an essential format for computing in AutoDock Vina [97].

#### 2.1.2. Protein Structure Selection and Preparation

All the studied target proteins were retrieved from the Protein Data Bank [98]. The PDB IDs of each protein and their corresponding atomic resolutions are given in Table 1. In order to prepare each protein structure, all structures were first checked for missed residues if present and repaired accordingly. Using the AutoDock Tools 1.5.6 program, all target proteins were prepared following the addition of hydrogen atoms, removal of hetero atoms, such as water and other cofactors, etc. The Gasteiger charge was also adjusted to all the protein structures. Finally, each crystal structure was insert into AutoDock Tools 1.5.6 [99], then converted to pdbqt format.

#### 2.1.3. Configuration File Generation and Molecular Docking Execution

For docking execution, a configuration file was created that contained the information of input parameters, such as protein and ligand information, size and center of grid box coordinates, and output file information. For docking execution, the AutoDock Vina tool was used, which was installed on a Windows operating system. Following a default setting, all the proteins were docked with the synthesized pyrazole derivatives, which generated a maximum of nine docked poses for each compound. Further, based on the best binding affinity score, potential pyrazole compounds were selected for subsequent modeling studies.

### 2.2. Molecular Dynamic Simulation

Molecular dynamic simulation is a broadly used approach to inspect inter- or intramolecular interactions at atomic level and describe the realistic motions in protein–complexes, which in turn provides an understanding of ligand-binding status in the physiological milieu [104]. The top-ranked protein–ligand complexes derived from molecular docking study were further subjected to molecular dynamic simulations using Desmond v.4.2 Molecular Dynamics System simulation wizard integrated in the Schrodinger suite LLC for a period of 1000 ns [105]. Firstly, the simulation system was solvated by encapsulating the protein–ligand complex within a default orthorhombic box of 10 Å containing the TIP3P (Transferable intermolecular interaction potential 3 points) water model using the “System builder” tool. In addition, appropriate numbers of counter ions were also adjusted to the protein–ligand complex system. A 0.15 M concentration of NaCl ions was adjusted to the molecular systems and allowed relaxation before the simulations. OPLS_2005 (optimized potentials for liquid simulations) force field parameters [106] were applied to the system. For minimization of the protein–ligand complex, each system was slowly equilibrated using NPT (isothermal–isobaric: number of atoms (N), pressure (P), and temperature (T) are conserved) ensemble and simulation run was carried out at NVT ensemble via shake algorithm under bar pressure of 1.013 and temperature of 300 K [107]. Applying the r-RESPA algorithm (reversible reference system propagation algorithm integrator) [108] the non-bonded interactions were evaluated with an update of short-range forces at every step and the long-range forces every three steps. During simulation runs, the particle mesh Ewald method (PME) [109] was applied in order to assess the long-range Coulomb interactions. For short-range Coulombic interaction evaluation, a cutoff radius 9.0 Å was defined. Finally, the trajectories were saved at 4.8 ps intervals for analysis. After completion of MD simulation runs, each trajectory was monitored by investigating various parameters, such as root-mean-square deviation (RMSD), root-mean-square fluctuation (RMSF), radius of gyration (RoG), position of the ligand in different frames, and distance between ligand and receptor.

### 2.3. In Silico Drug-Likeness and ADME_T Profiles Estimation

The web servers SwissADME [110] and pkCSM [111] were used for computing the drug-likeness, pharmacokinetics, ADME (absorption, distribution, metabolism, excretion), and toxicity profile of all pyrazole derivatives. The SMILE format of the studied compounds was given as input in both web tools in order to estimate the abovementioned profiles and then checked for compliance with their standard ranges. SwissADME, a highly regarded application tool tremendously being used in computational drug discovery to examine drug-likeness (Lipinski’s rule [112] of five, and Veber’s rule [113]) and other pharmacokinetic properties, such as metabolism. Absorption, distribution, excretion, and toxicity properties were assessed via pkCSM. The pkCSM tool uses a graph-based signature technique to estimate various parameters. The predictive models of validated pharmacokinetic and toxicological aspects, such as AMES toxicity, maximum tolerated dose (human), hERG-I/hERG-II inhibitor, oral rat acute various toxicity parameters such as AMES toxicity, oral rat chronic toxicity (LOAEL), hepatotoxicity, skin sensitization, *T. pyriformis* toxicity, and minnow toxicity, were successfully estimated via this tool.

## 3. Results and Discussion

A structure-based virtual screening (SBVS) of the synthesized pyrazole derivatives (the structures of the 63 considered molecules and their chemical spectroscopy are displayed in the Supplementary Information) [7,8,9,10,11,12,13,14,15,16,17,18] was adopted to identify potential therapeutic pyrazole derivatives compounds against six cancer protein targets: CRMP2, C-RAF, CYP17, c-KIT, VEGFR, and HDAC.

Through molecular docking, we filtered out the less active pyrazole compounds against each target protein based on their binding. Standard drugs or known inhibitors against the five studied proteins were also included for comparison of the study outcomes and also for better selection purposes. The approved drugs and known inhibitors chosen for the current study were nalidixic acid (CRMP2 agonist), sorafenib (RAF kinase inhibitor, and more selective on C-Raf than the other isoforms of RAF kinase), abiraterone acetate (CYP17A1 inhibitor), orteronel (inhibitor of CYP 17,20-lyase), galeterone (also inhibitor of CYP-17), sunitinib (modulates both C-KIT and VEGFR), SAHA (inhibitor of HDAC-9), and TMP269 (TMP269 inhibitor across HDAC1–11). (For more details and references, refer to the introduction section).

The outcome analyses reveal that the top-ranked small molecules identified among our pyrazole derivatives were M74 (potential modulator of CRMP2), M36 (for E-RAF), M72 (for CYP17), M76 (for VEGFR), M74 (for C-KIT), and M33 (potential inhibitor of HDAC).

In order to further explore the stability and binding interactions in physiological milieu at atomic level, a long-range atomic molecular dynamic (MD) simulation (1000 ns) for the top-ranked complexes was carried out for all the best complexes. The findings for both molecular docking and molecular dynamic simulation are displayed and discussed below.

**Figure 1:**
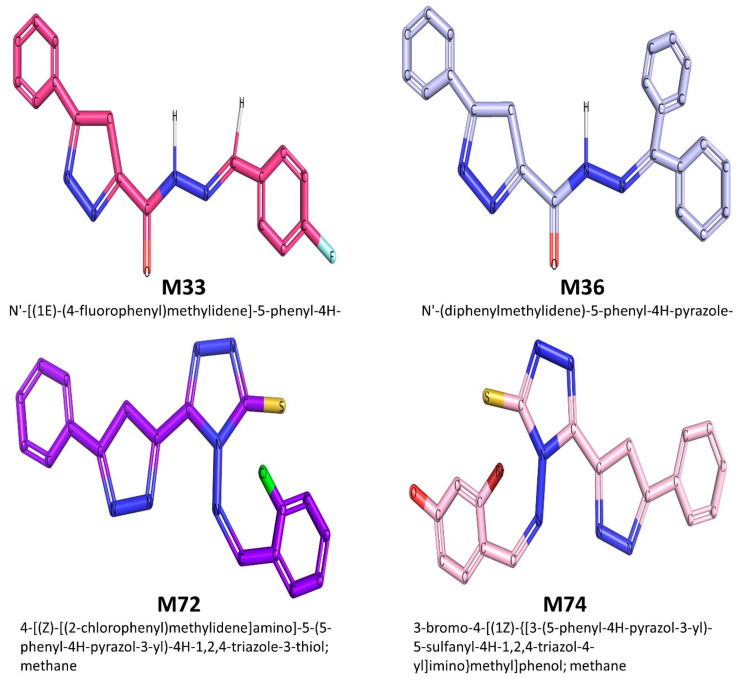
The top-ranked compounds selected from the 63 synthesized pyrazoles derivatives.

The two dimensional (2D) chemical structure of the top-ranked active pyrazole compounds selected against the studied protein targets is given in Figure 1.

### 3.1. Binding Interaction Profile Analysis of CRMP2 and Pyrazole Derivative Complex

**Figure 2:**
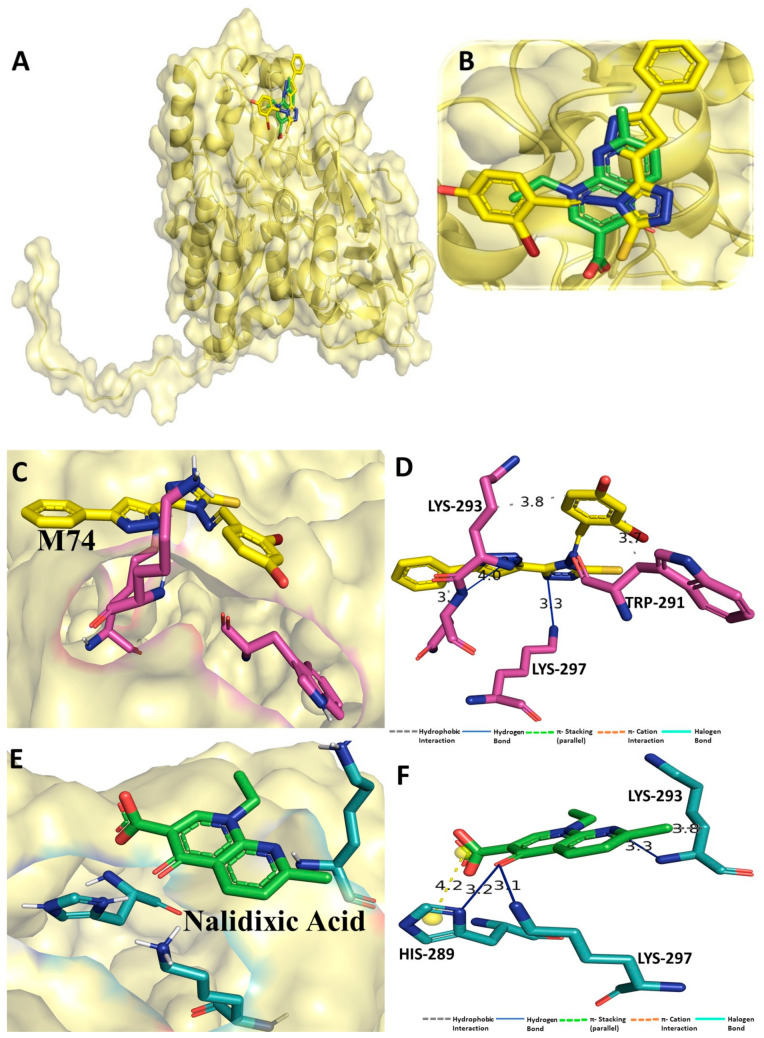
(**A**) Molecular docking-generated binding orientations of standard compound (nalidixic acid) and the proposed pyrazole-based inhibitor compound M74 in complex with CRMP2 (6JV9) protein; (**B**) close-up binding interaction view exhibiting similar binding pattern of all the compounds at the active site cavity of CRMP2 (6JV9) protein; (**C**) binding mode of pyrazole compound M74 in the active site cavity of CRMP2 (6JV9) displayed in surface view representation; (**D**) molecular binding interaction and orientation of compound M74 with CRMP2 (6JV9) protein; (**E**) binding mode of standard compound (nalidixic acid) in the active site cavity of CRMP2 (6JV9) displayed in surface view representation; (**F**) molecular binding interaction and orientation of nalidixic acid with CRMP2 (6JV9) protein.

Molecular docking was performed for all 63pyrazole derivatives with CRMP2 (PDB ID: 6JV9), and docking scores ranged from −4.1 to −7 kcal/mol. With the lowest docking score of −6.9 kcal/mol, the binding affinity of pyrazole derivative M74 was found to be the most important among all pyrazoles series to interact with CRMP2. On the other hand, the standard drug compound nalidixic acid was also docked with CRMP2, which revealed relatively lower binding affinity score of −5.0 kcal/mol compared to M74. Although the majority of the compounds were successfully docked in the active site region of the CRMP2, M74 showed considerable intermolecular interaction with CRMP2. In particular, the hydrogen bonding (H-bond) interaction was found with residue backbone chain Asn294 (Figure 2) and –NH group of M74 compound. Along with the H-bond interaction, some important residues, such as Ser292, Lys293, and Lys297, were also found to be establishing hydrophobic interactions with CRMP2. Yuying Wang et. al. reported similar residue involvement in the formation of H-bond interaction with the antiepileptic drug lacosamide [95]. Certainly, compound M74 showed promising binding interaction at the receptor cavity, which might help in modulating biological action. Binding scores of all the pyrazole derivatives, including the standard drug compounds, are listed in Table S1 (Supplementary Information).

### 3.2. Molecular Dynamic Simulation Analysis of CRMP2–Pyrazole Derivative M74 Complex

**Figure 3:**
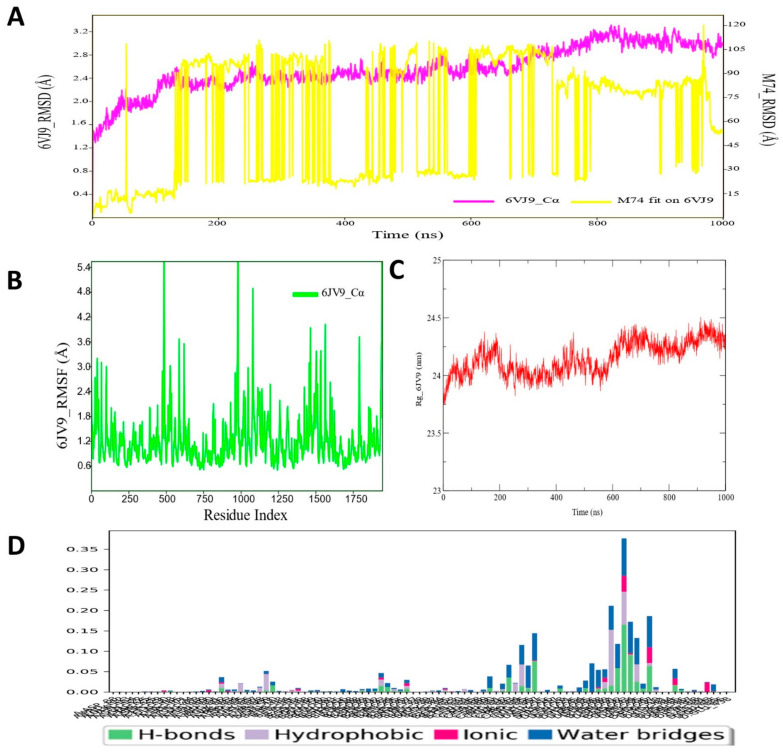
(**A**) RMSD profile of CRMP2 (6JV9) protein backbone and compound M74 during 1000 ns simulation span; (**B**) RMSF profile of CRMP2 (6JV9) protein backbone during 1000 ns simulation span; (**C**) RoG profile of CRMP2 (6JV9) protein backbone during 1000 ns simulation span; (**D**) illustration of the CRMP2 (6JV9)–M74 contacts or interactions map monitored during 1000 ns simulation run.

A long range (up to 1000 ns) molecular dynamic simulation of the protein ligand (CRMP2–M74) complex was critically analyzed and various important parameters, such as RMSD, RMSF, and interaction contact map, were deduced to evaluate the dynamic stability of the bound ligand M74. The RMSD values of CRMP2 backbone atom and ligand M74 are plotted in Figure 3. The simulated protein–ligand complex appeared to be oscillated for a certain period in the initial phase; however, the RMSD for the CRMP2 protein backbone gradually achieved a stable state at the end of the simulation run. The RMSD values of the ligand M74 atoms were found to be highly fluctuated during the simulation run, perhaps indicating that the ligand was probably either getting a better orientation to fit at the binding pocket of CRMP2 or diffused away from its initial binding site. Such instances were clarified through exploration of final MD simulated state of the protein–ligand complex (i.e., trajectory at 1000 ns), which revealed that the ligand remained at the binding-site region of CRMP2. Therefore, it can be said that although the ligand RMSD fluctuated on a relatively high scale, those fluctuations did not hamper the ligand stability at larger magnitudes for interaction with the CRPM2 protein for longer periods. In particular, the average protein backbone RMSD value was found to be 2.67 Å, whereas for the compound M74, average RMSD was 75.5 Å. The RMSF values of each amino residue of CRMP2 bound with pyrazole compound M74 were recorded. RMSF values of the protein backbone showed that nearly all residues of CRMP2 had moderate fluctuation in a few regions (~ at 484–496, 956–967, 1078–1086) during the simulation run, displayed in Figure 3A. Figure 3B shows the RoG profile of the CRMP2 backbone during simulation bound with compound M74, demonstrating a stable folding nature of the protein backbone in dynamic conditions. The protein–ligand contacts/interactions were also explored during MD simulation studies for all the protein–ligand complexes, including CRMP2 protein (Figure 3C). MD simulation analyses also explored the protein–ligand contacts map of the M74–CRMP2 complex to see how and whether the docking-obtained interactions remained unchanged or during MD simulation such interactions were abolished and/or formed newer types of interactions with CRMP2. Interestingly, few amino acid residues, such as Ser292, Lys293, Asn294, and Lys297, were found to form hydrogen bond interactions with CRMP2, as observed in MD simulation analyses. Most importantly, all those interactions remained in place for almost 80–90% of the time in the dynamic environment (Figure 3D). 

### 3.3. Binding Interaction Profile of C-RAF and Pyrazole Derivative Complex

**Figure 4:**
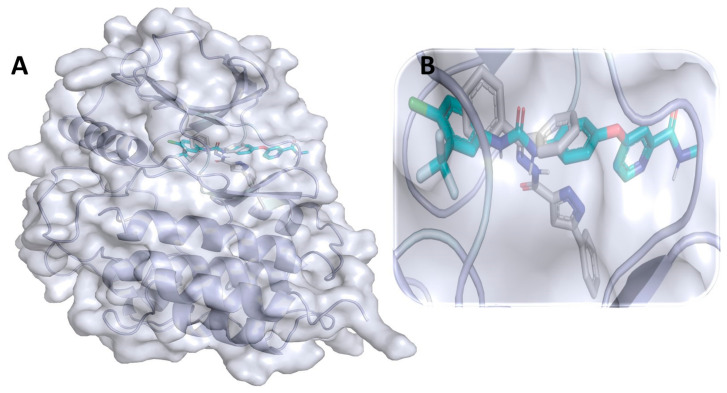

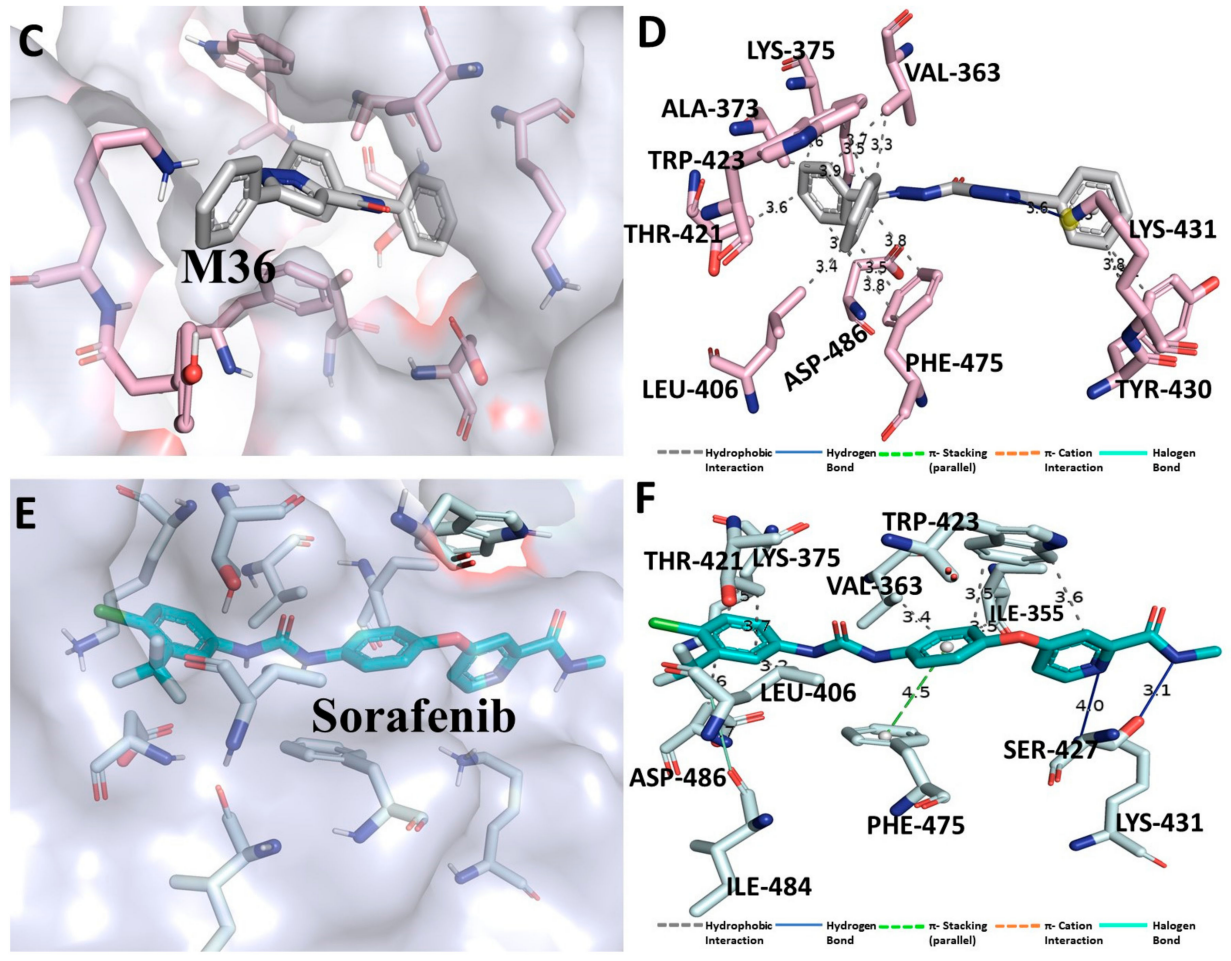
(**A**) Molecular docking-generated binding orientations of standard compound (sorafenib) and the proposed pyrazole-based inhibitor compound M36 in complex with C-RAF (3OMV) protein; (**B**) close-up binding interaction view exhibiting similar binding pattern of all the compounds at the active site cavity of C-RAF (3OMV) protein; (**C**) binding mode of pyrazole compound M36 in the active site cavity of C-RAF (3OMV) in surface view; (**D**) molecular binding interaction and orientation of compound M36 with C-RAF (3OMV) protein; (**E**) binding mode of standard compound (sorafenib) in the active site cavity of C-RAF (3OMV) displayed in surface view; (**F**) molecular binding interaction and orientation of sorafenib with C-RAF (3OMV) protein.

The molecular docking-based binding mode and orientation of C-RAF protein with all pyrazole derivatives was explored for identifying the compound with the most potential that interacted with C-RAF protein. Molecular docking of the selective drug compound sorafenib was also carried out, which revealed a binding affinity score of −10.2 kcal/mol. However, among all the 63 pyrazole derivatives, compound M36 exhibited the lowest binding affinity score (i.e., −9.7 kcal/mol), which was nearer to the binding score of the standard compound. In docking, M36 interacted with several active site amino acid residues, such as Phe475 and Asp486 of the C-RAF protein, to established numbers of hydrophobic contacts with M36 (Figure 4). Kaboli et al. demonstrated the importance of amino acid residues Phe475 and Asp486 of C-RAF protein for establishment of intermolecular interaction with alkaloid compound berberine using docking [114]. They also used the same PDB ID: 3OMV for conducting the in silico investigation. Kim et al. also confirmed the importance of intermolecular interaction with the surrounding residues of the DFG region of C-RAF. They demonstrated that the residue Asp486 interacted strongly with the compound 3-carbonyl-5-phenyl-1H-pyrazole for exhibiting potential inhibitory activity against C-RAF protein [114]. Docking affinity scores for all other compounds are given in Table S2 (Supplementary File).

### 3.4. Molecular Dynamic Simulation Analysis of C-RAF–Pyrazole Derivative M36 Complex

**Figure 5:**
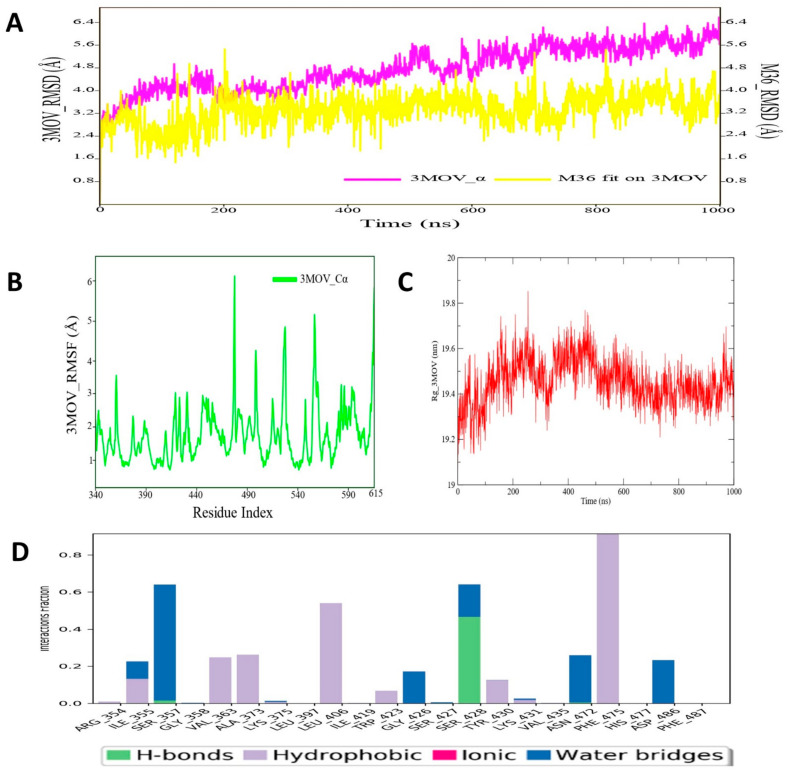
(**A**) RMSD profile of C-RAF (3OMV) protein backbone and compound M36 during 1000 ns simulation span; (**B**) RMSF profile of C-RAF (3OMV) protein backbone during 1000 ns simulation span; (**C**) RoG profile of C-CRAF protein backbone during 1000 ns simulation span; (**D**) illustration of the C-RAF (3OMV)–M36 contacts or interaction map monitored during 1000 ns simulation run.

The MD simulated RMSD plot for C-RAF protein backbone atoms and ligand M36 was calculated from 1000 ns simulation trajectories and is depicted in Figure 5. The RMSD plot of the abovementioned protein–ligand complex revealed that there was no such noticeable fluctuation in RMSD values in either protein backbone or ligand M36. It was found that for the entire simulation run period that the C-RAF protein maintained a stable conformation organization till the simulation end. Similar types of RMSD profile were also observed for the ligand M36 atoms, as no high-magnitude fluctuation was observed during MD simulation. The average protein backbone RMSD value was found to be 4.98 Å for C-RAF protein, and for compound M36, the average RMSD value was 3.99 Å. Such low RMSD values throughout the MD simulation run undoubtedly indicated substantial consistency and conformational integrity in both macromolecular complex structures. Kaboli et al. also demonstrated a similar RMSD profile and other thermodynamic parameters for C-RAF backbone atoms for 10,000 ps MD simulation time span of CRAF–berberine complexes [114]. Another important parameter, RMSF, which was also measured for the C-RAF protein backbone atoms, revealed less fluctuation in backbone atoms during simulation (Figure 5). Some regions of the C-RAF protein (at ~381–389, 489–496, 526–537) were found to deviate a little bit, as observed from the RMSF values. Such fluctuation might be due to no involvement of intermolecular interaction with M36 at the particular region of C-RAF protein. On the other hand, the crucial amino acid residues Phe475 and Asp486 showed much less fluctuation during the entire MD simulation span. The RoG values for the protein backbone were obtained to evaluate the structural compactness of the simulated C-RAF protein and are depicted in Figure 5. The RoG values demonstrated that although there was high fluctuation observed at the initial span of simulation (during 200–300 ns), afterward fluctuation achieved a plateau state and stayed the same till the simulation end. Furthermore, the protein–ligand contacts or interactions map was derived from the entire simulation trajectories, which revealed that during MD simulation of the protein–ligand complex, several intermolecular interactions were formed: residue Ser428 formed an H-bond, residues Ser357, Asn472, and Asn486 formed water bridges, and hydrophobic residues Val363, Ala373, Leu406, and Phe475 of C-RAF protein established hydrophobic contacts with M36 (Figure 5). All these interactions were maintained for at least ~50–80% of the time in the dynamic environment. During the entire simulation run, all those intermolecular interactions were found to persist for approximately 40–50% of the time. Therefore, findings for the C-RAF protein and ligand M36 MD simulation study supported overall structural and interaction stability of the studied macromolecular complex that can deliver essential biological action for modulating the role of the C-RAF protein.

### 3.5. Binding Interaction Profile Analysis of CYP17 and Pyrazole Derivative Complex

**Figure 6:**
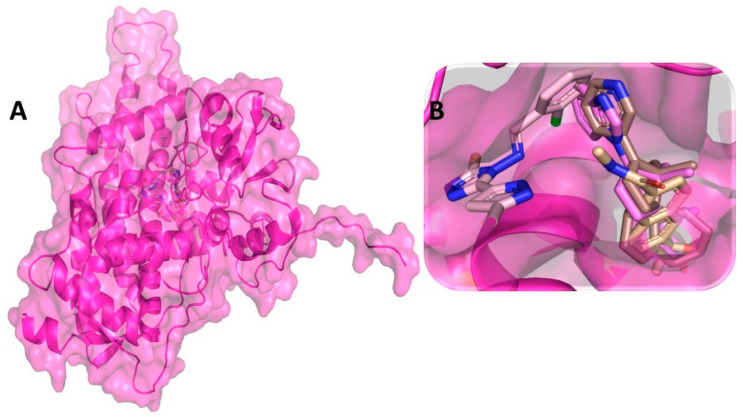

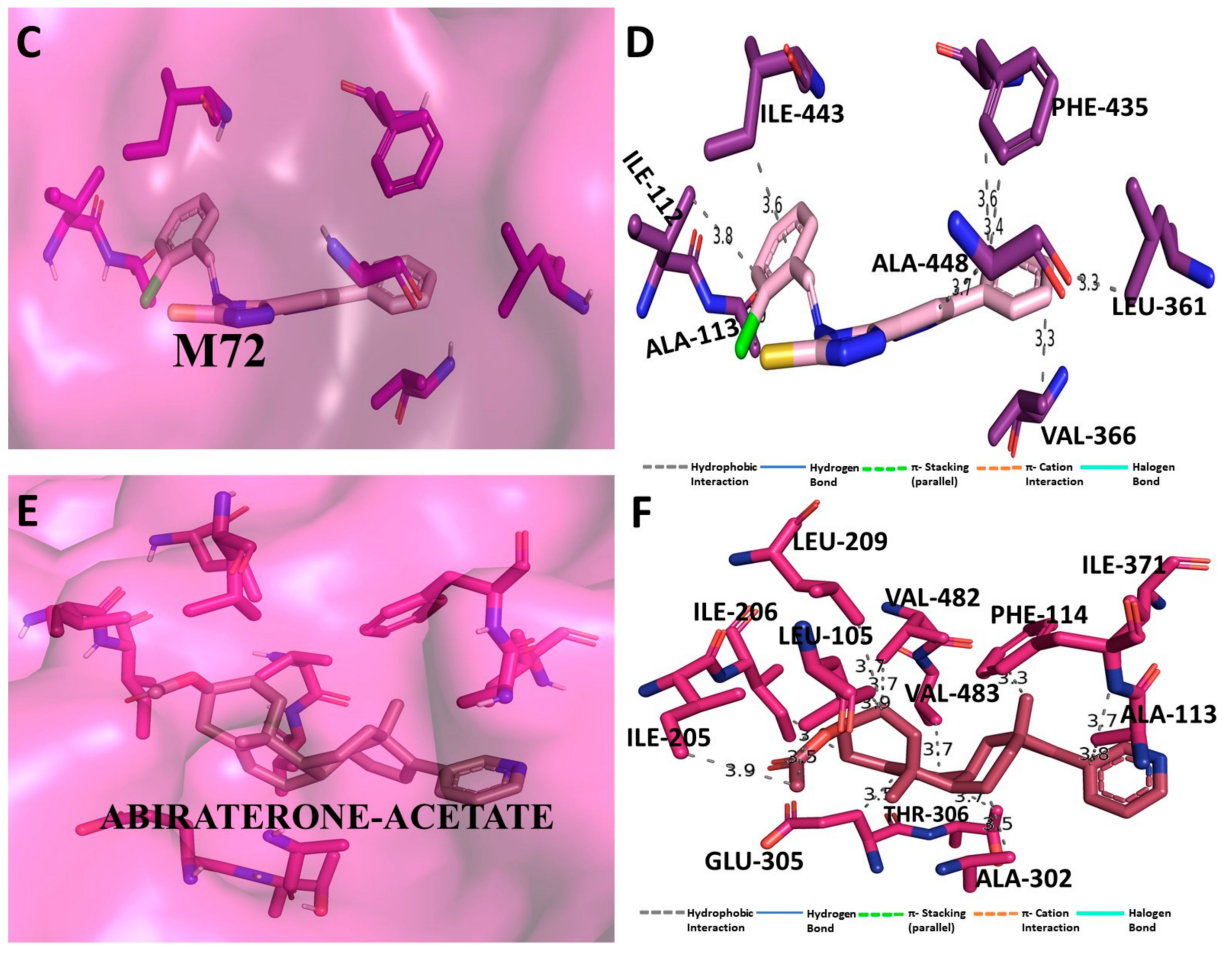

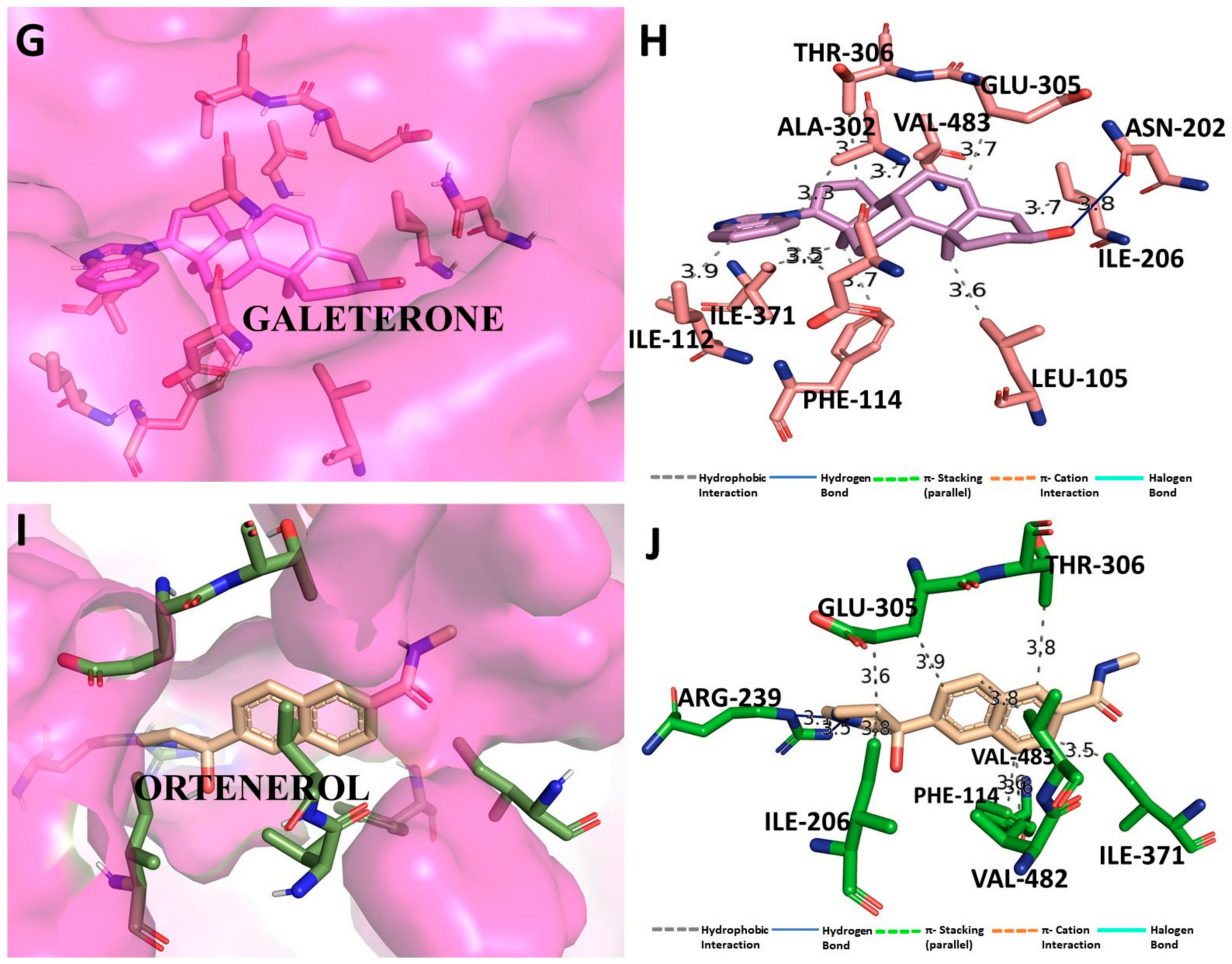
(**A**) Molecular docking-generated binding orientations of all standard compounds (abiraterone acetate, galeterone, orteronel) and the proposed pyrazole-based inhibitor compound M72 in complex with CYP17 (4NKV) protein; (**B**) close-up binding interaction view exhibiting similar binding pattern of all the standard compounds including M72 at the active site cavity of CYP17 (4NKV) protein; (**C**) binding mode of pyrazole compound M72 in the active site cavity of CYP17 (4NKV) displayed in surface view; (**D**) molecular binding interaction and orientation of compound M72 with CYP17 (4NKV) protein; (**E**) binding mode of standard compound (abiraterone acetate) in the active site cavity of CYP17 (4NKV) displayed in surface view; (**F**) molecular binding interaction and orientation of abiraterone acetate with CYP17 (4NKV) protein; (**G**) binding mode of standard compound galeterone in active site cavity of CYP17 (4NKV) displayed in surface view; (**H**) molecular binding interaction and orientation of galeterone with CYP17 (4NKV) protein; (**I**) binding mode of standard compound orteronel in active site cavity of CYP17 (4NKV) displayed in surface view; (**J**) molecular binding interaction and orientation of orteronel with CYP17 (4NKV) protein.

Binding interaction of CYP17 protein with all the 63 pyrazole derivatives analyzed through molecular docking revealed quite strong binding interaction affinity with some pyrazole derivatives, including M72, which turned out to be the compound with the most potential. For protein CYP17, the docking-based binding affinity scores of the pyrazole derivatives were within the range of −3.7 to −10.4 kcal/mol. Molecular docking performed with the standard drug compounds galeterone and olaparib revealed binding affinity scores of −11.6 and −11.4 kcal/mol, respectively. Binding affinity scores of all the 63 pyrazole compounds docked with CYP17 are listed in Table S3 (Supplementary Information). Compound M72 exhibited a binding affinity score of −10.4 kcal/mol and it was found to be majorly involved through hydrophobic interaction with several of the amino acid residues: Ile112, Ala113, Phe132, Ile299, Ala302, Phe435, Leu361, Val366, Cys442, Ile443, Gly444, Leu447, and Ala448 of CYP17 protein Figure 6. In a prior work reported by Al-Masoudi et al., molecular docking investigation of CYP17 with various drugs yielded a binding mechanism that was comparable to that of compound M72 [115]. It was interesting to note that compound M72 appeared to be completely wrapped by many active site amino acid residues for making such type of intermolecular interaction network at the active site pocket.

### 3.6. Molecular Dynamic Simulation Analysis of CYP17–Pyrazole Derivative M72 Complex

**Figure 7:**
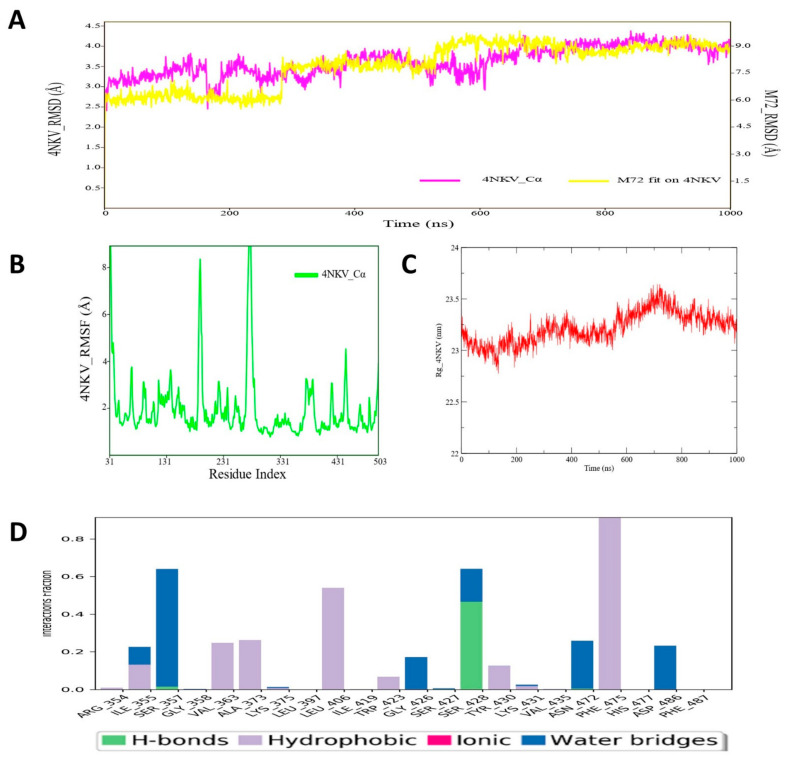
(**A**) RMSD profile of CYP17 (4NKV) protein backbone and compound M72 during 1000 ns simulation; (**B**) RMSF profile of CYP17 (4NKV) protein backbone during 1000 ns simulation; (**C**) RoG profile of CYP17 (4NKV) protein backbone during 1000 ns simulation; (**D**) illustration of the CYP17 (4NKV)–M72 contacts or interactions map monitored during 1000 ns simulation.

MD simulation analysis revealed an interesting observation for the CYP17-M72 protein–ligand complex. Obtaining the RMSD plot revealed that initially the RMSD values for both the protein backbone atoms and the ligand atoms were quite low; however, at the later phase (~400 ns) of MD simulation, a relatively higher oscillation or deviation was noticed in RMSD values for CYP17 as well as ligand M72 atoms (Figure 7). The average protein backbone RMSD was 3.67 Å, and for compound M72, average RMSD was estimated as 7.899 Å. The RMSF value of protein backbone of each amino residue of CYP17 bound with compound M72 was recorded and is displayed in Figure 7. The RMSF values showed that a majority of the amino acid residues of CYP17 showed less fluctuation throughout the simulation run period, which might indicate the structural integrity of the protein with the bound state of compound M72. Another important MD trajectory analysis parameter, i.e., RoG, was also measured to estimate the structural rigidity and compactness of the simulated CYP17 protein backbone and the RoG values are depicted in Figure 7. The RoG of CYP17 revealed consistent values for most of the time span; however, for a small period (~600–700 ns), there was little fluctuation observed, which indicated a reorganization of protein backbone for governing better compactness to the CYP17 structure. The protein–ligand contact map of the CYP17-M72 complex was derived from simulation trajectories (Figure 7) revealed that during MD simulation, amino acid residue Arg96 formed an H-bond interaction that was maintained for almost 80–90% of time span in the dynamic environment (Figure 7A). Amino acids Ile 371, Phe435, and Arg440 were also found to form hydrophobic and water bridge interactions with compound M72. All the hydrophobic and water bridge intermolecular interactions were found to persist for approximately 40–50% of the time during the simulation run. Overall, the MD simulation study suggested that the pyrazole derivative M72 might possibly mediate enough stability in terms of intermolecular interaction with the CYP17 protein in the dynamic condition and hence could contribute towards newer therapeutic agents for development of potential inhibitors of CYP17 protein.

### 3.7. Binding Interaction Profile Analysis of VEGFR and Pyrazole Derivative Complex

**Figure 8:**
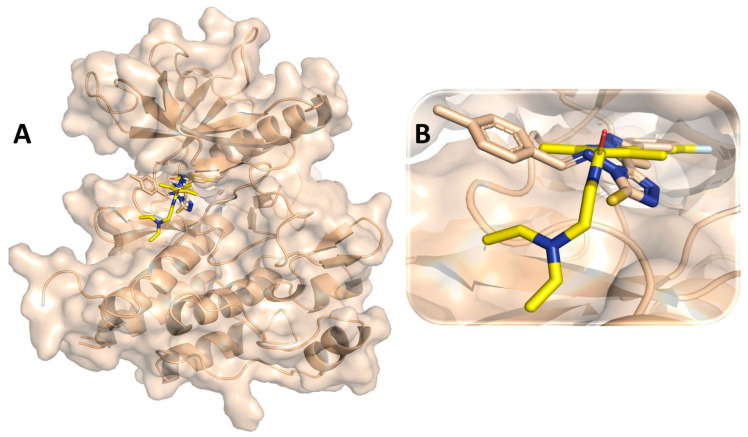

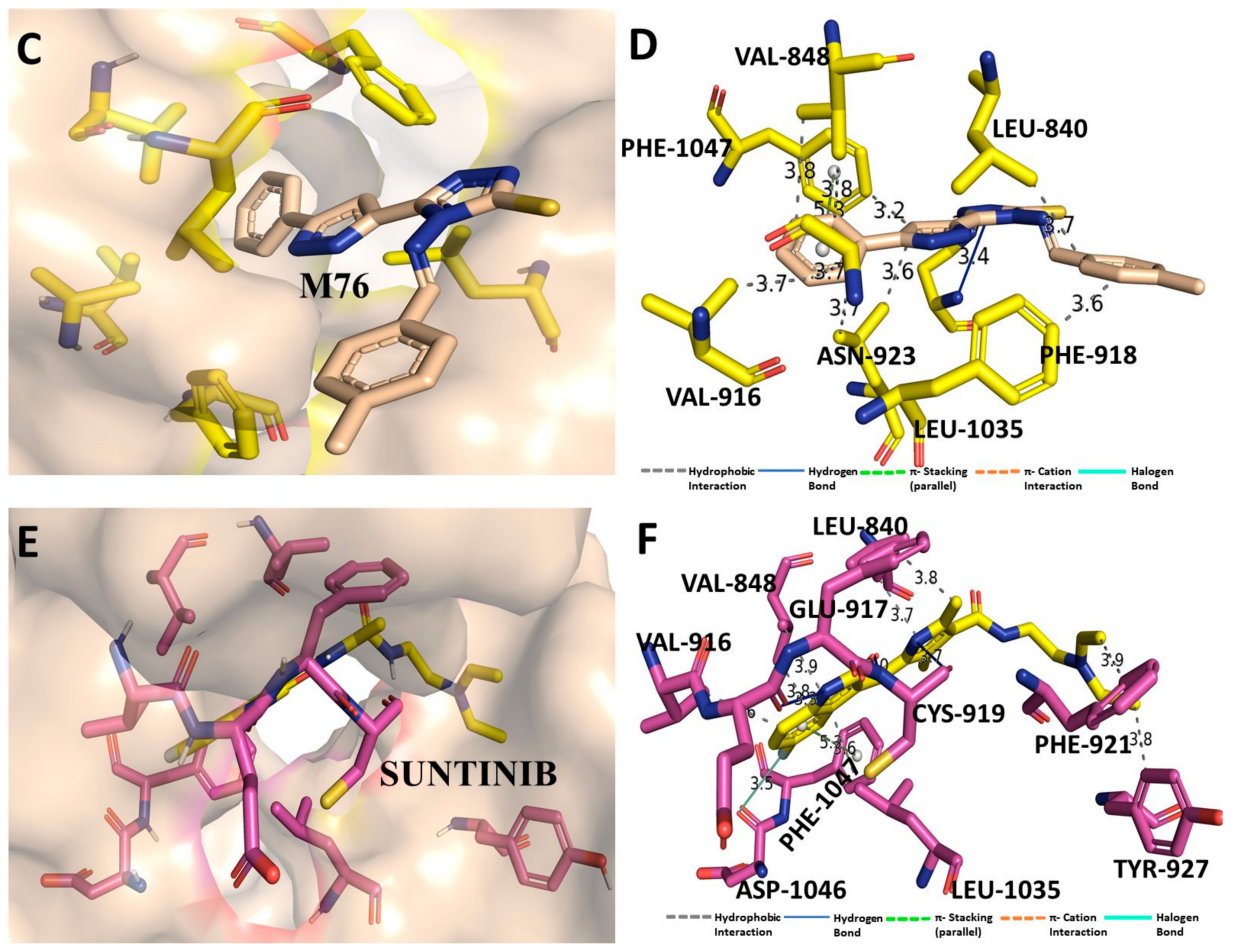
(**A**) Molecular docking-generated binding orientations of standard compound (sunitinib) and the proposed pyrazole-based inhibitor compound M76 in complex with VEGFR (4AGD) protein; (**B**) close-up binding interaction view exhibiting similar binding pattern of all the compounds at the active site cavity of VEGFR (4AGD) protein; (**C**) binding mode of pyrazole compound M76 in the active site cavity of VEGFR (4AGD) displayed in surface view; (**D**) molecular binding interaction and orientation of compound M76 with VEGFR (4AGD) protein; (**E**) binding mode of standard compound (sunitinib) in the active site cavity of VEGFR (4AGD) displayed in surface view; (**F**) molecular binding interaction and orientation of sunitinib with VEGFR (4AGD) protein.

All 63 pyrazole derivatives docked with VEGFR (PDB ID: 4AGD) using the Auto Dock Vina tool revealed a strong binding interaction affinity for many compounds. In addition to that, the standard drug compounds sunitinib and pazopanib were also docked with VEGFR and generated binding affinity scores of −10.0 and −9.9 kcal/mol, respectively. Molecular docking-based binding affinity analyses of all pyrazole derivatives compounds revealed that compound M76 showed the lowest binding affinity score: −9.2 kcal/mol. Binding affinity scores of all pyrazole compounds with VEGFR are shown in Table S4 (Supplementary Information). Molecular binding interaction of compound M76 with VEGFR was critically analyzed and revealed that a single H-bond interaction was formed between amino acid residue Asn923 and the –NH group of compound M76 Figure 8. Other than the H-bond interaction, several hydrophobic interactions were found, which were mostly mediated through amino acid residues Leu840, Val848, Ala866, Phe918, Leu1035 and Phe1047 of VEGFR. A docking study-based investigation previously showed that most residues of VEGFR mentioned above interacted with compounds axitinib, flubendazole, rilpivirine, and papaverine at the active site residues of VEGFR [116]. All these residues are present in close vicinity of the active site region of VEGFR. Therefore, any intermolecular interactions with those residues might have some impact on modulating the activity of the receptor (Figure 8).

### 3.8. Molecular Dynamic Simulation Analysis of VEGFR–Pyrazole Derivative M76 Complex

**Figure 9:**
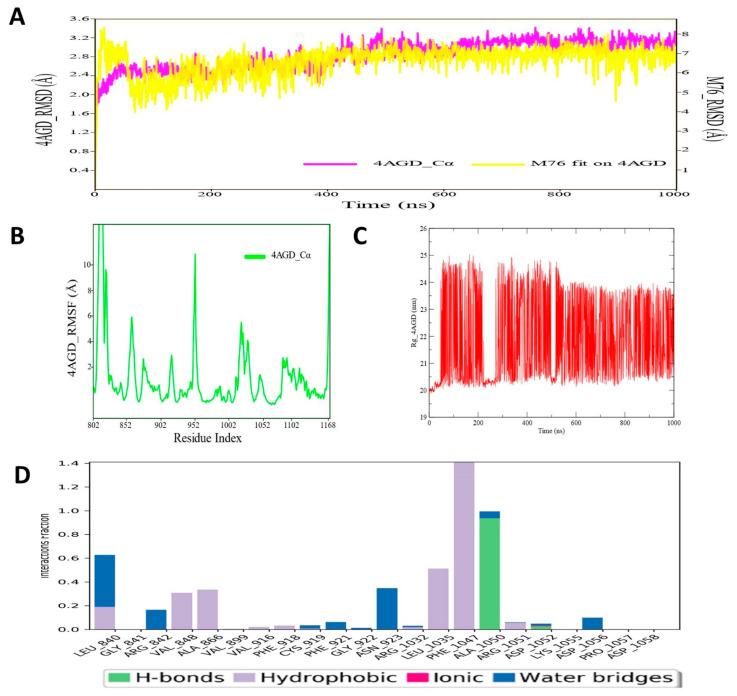
(**A**) RMSD profile of VEGFR (4AGD) protein backbone and compound M76 during 1000 ns simulation; (**B**) RMSF profile of VEGFR (4AGD) protein backbone during 1000 ns simulation; (**C**) RoG profile of VEGFR (4AGD) protein backbone during 1000 ns simulation; (**D**) illustration of the VEGFR (4AGD)–M76 contacts or interactions map monitored during 1000 ns simulation.

MD simulation analysis of VEGFR–pyrazole derivative M76 complex was carried out for 1000 ns and RMSD, RMSF and RoG profiles analyzed from the simulated trajectories. The RMSD values of VEGFR protein backbone atoms and ligand M76 were plotted and are given in Figure 9. From the RMSD plot of the VEGFR protein backbone and ligand M76, it was noticed that there was no noticeable deviation in the molecular system in the dynamic environment. Moreover, throughout the simulation run, the RMSD values of VEGFR protein backbone atoms and ligand M76 remained quite stable, indicating that compound M76 probably formed a strong interaction profile with VEGFR. The average protein backbone RMSD value for VEGFR and compound M76 was 2.96 and 7.86 Å, respectively. Meng et al. in an MD simulation study of VEGFR kinase–sorafenib complex illustrated that simulated trajectories were stabilized after 500 ps and maintained the same till the simulation end [117]. Another study reported only 20 ns MD simulation with the compound ZINC00759038, as the potent VEGFR inhibitor also showed quite stable and similar types of RMSD profile for VEGFR protein throughout the simulation run time. The RMSF values of each amino acid residue of VEGFR protein bound with compound M76 were recorded and are displayed in Figure 9. The RMSF of the protein backbone indicated that nearly all residues of VEGFR showed little fluctuation except for the region around 940–986 (which did not participate in any interaction), with M76 fluctuating slightly higher. Amino acid residues Leu840, Val848, Ala866, Phe918, Leu1035 and Phe1047 showed much less fluctuation than other residues of VEGFR, which suggested considerable conformational stability along the protein backbone of VEGFR during the MD simulation time span. In order to assess the structural compactness of the simulated VEGFR protein, the RoG profile was measured and is depicted in Figure 9. Values were consistent for the entire simulation. Though a slightly high RoG value was observed throughout the MD simulation, the magnitude of such oscillation did not suggest any disturbance in the folding state of the protein in the dynamic condition. The protein–ligand contacts/interactions were also deduced during MD simulation of the protein–ligand complex of VEGFR protein. The protein–ligand contacts map of the compound M76–VEGFR exhibited an interesting result, which highly corroborated with the binding interaction profiles of the docking investigation (Figure 8D). The amino acid residuesLeu1035 and Phe1047, which have been found to form hydrophobic interactions, herein, MD simulation showed a similar interaction pattern for almost 80–90% of the time span in the dynamic environment (Figure 9). Ala1050 was also found to be involved in the H-bond interaction. Leu840, Val848, and Ala866 were found to form hydrophobic or water bridge interactions with M76. Such intermolecular interactions persisted for approximately 40–50% during the entire simulation run. As observed and analyzed, the pyrazole derivative M76 can give enough stability to the VEGFR protein under dynamic conditions, which can aid medicinal chemistry research for the development of improved or prospective VEGFR inhibitors.

### 3.9. Binding Interaction Profile Analysis of c-Kit–Pyrazole Derivative Complex

**Figure 10:**
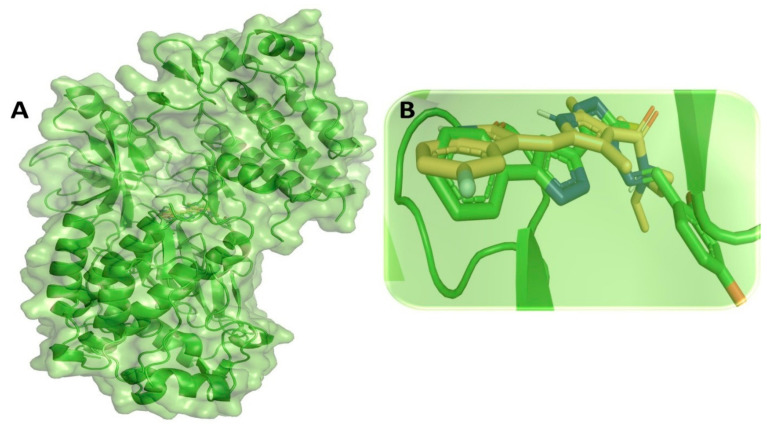

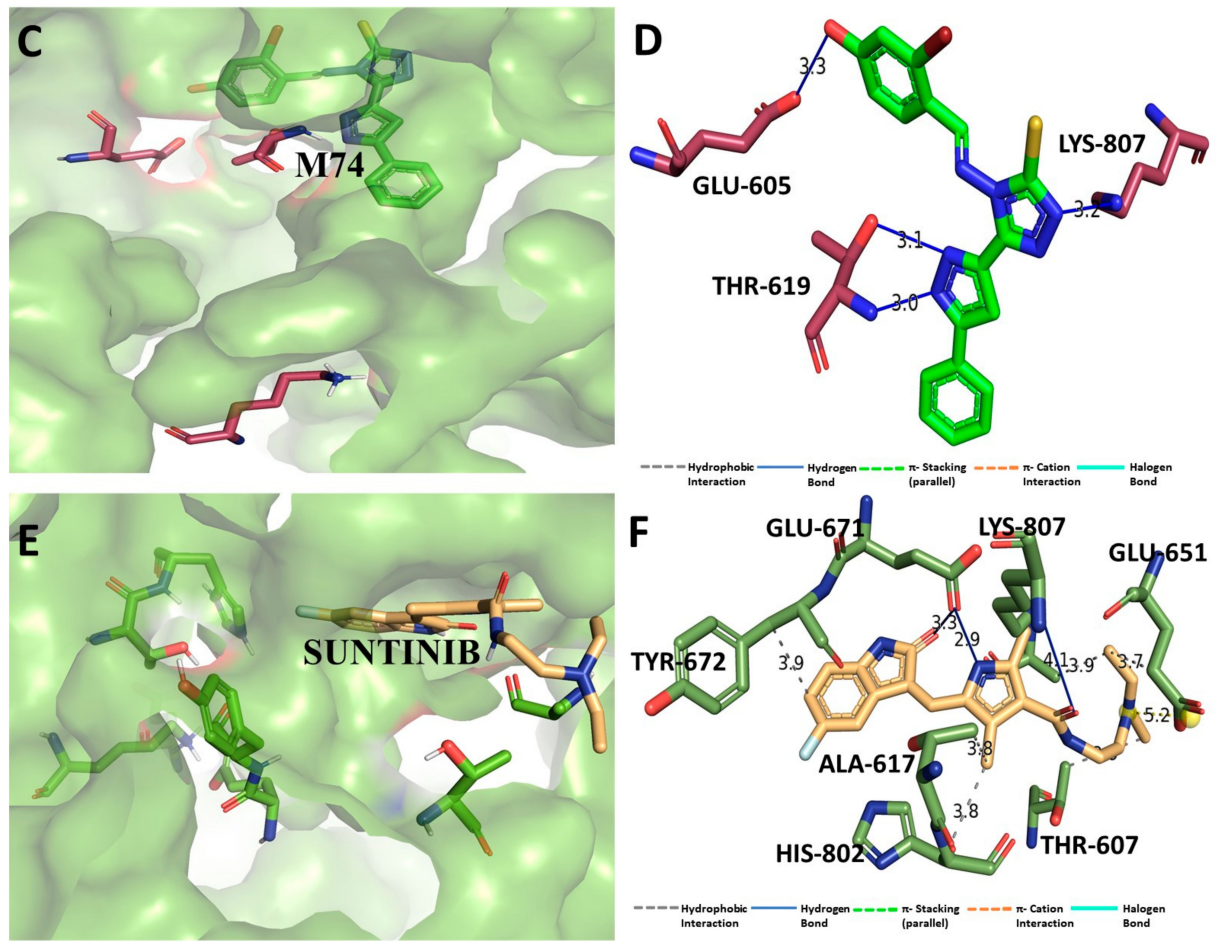
(**A**) Molecular docking-generated binding orientations of standard compound (sunitinib) and the proposed pyrazole-based inhibitor compound M74 in complex with c-KIT (6XVB) protein; (**B**) close-up binding interaction view exhibiting similar binding pattern of all the compounds at the active site cavity of c-KIT (6XVB) protein; (**C**) binding mode of pyrazole compound M74 in the active site cavity of c-KIT (6XVB) displayed in surface view; (**D**) molecular binding interaction and orientation of compound M74 with c-KIT (6XVB) protein; (**E**) binding mode of standard compound (sunitinib) in the active site cavity of c-KIT (6XVB) displayed in surface view; (**F**) molecular binding interaction and orientation of sunitinib with c-KIT (6XVB) protein.

Molecular docking was performed to explore the binding orientation of the pyrazole compounds to the active site region of c-KIT protein (PDB ID: 6XVB), revealing that compound M74 exhibited the lowest binding affinity score among all studied compounds including the standard compound pazopanib. The molecular docking-generated binding affinity scores for pazopanib and M74 were −8.7 and −9.2 kcal/mol, respectively. The binding mode of the best-binding-affinity complex was analyzed, which revealed that compound M74 docked into the binding site of the c-KIT and established intermolecular interactions of H-bonding and hydrophobic interaction with different amino acid residues of the c-KIT protein. Compound M74 showed H-bond interactions with amino acid residues Thr619 and Lys807 of c-KIT protein. Interestingly, the pyrazole ring of M74 participated to formed an H-bond interaction with residue Thr619, which is mediated by the -NH group (Figure 10). Ala617, Met618, Tyr672, and Ile805 of c-KIT protein were found to be involved in hydrophobic interaction. Binding affinity scores of all pyrazole compounds and the standard drug against c-KIT are presented in Table S5 (Supplementary Information).

### 3.10. Molecular Dynamic Simulation Analyses of c-Kit–Pyrazole Derivative M74 Complex

**Figure 11:**
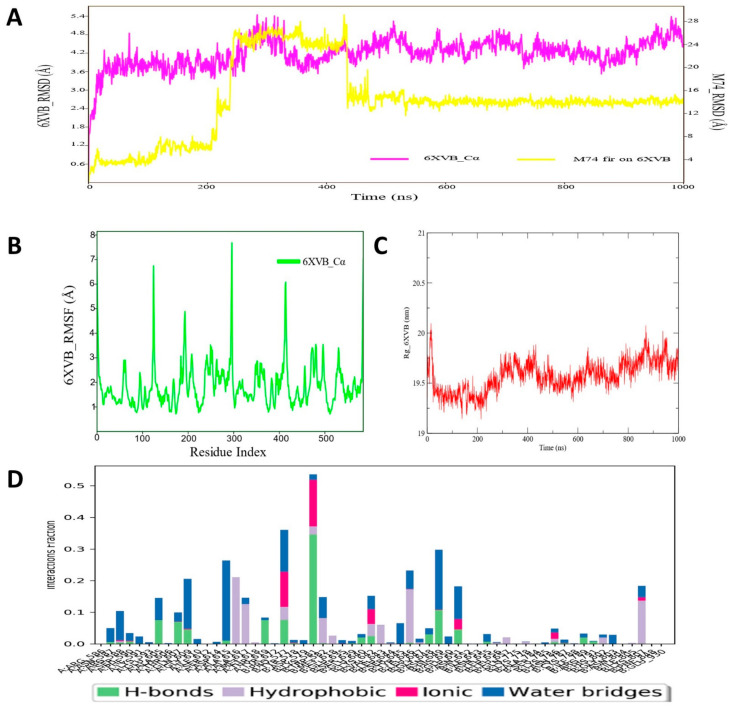
(**A**) RMSD profile of c-KIT (6XVB) protein backbone and compound M74 during 1000 ns simulation; (**B**) RMSF profile of c-KIT (6XVB) protein backbone during 1000 ns simulation; (**C**) RoG profile of c-KIT (6XVB) protein backbone during 1000 ns simulation; (**D**) illustration of the c-KIT (6XVB)–M74 contacts or interactions map monitored during 1000 ns simulation.

The RMSD values were analyzed to check the stability of the c-KIT protein and ligand M74 compound, which revealed that during MD simulation, the protein backbone remained low and consistent RMSD values were observed for c-Kit. However, the ligand M74 showed increased deviation in RMSD till ~420 ns, and thereafter reached a plateau state, indicating molecular system equilibration in the long run (Figure 11). After a 420 ns run, there was no high oscillation observed in RMSD values of ligand M74 atoms. The average protein backbone RMSD value was found to be 4.67 Å, whereas for the compound M74 the average RMSD value was 13.8 Å. The RMSF values of each amino acid of c-KIT bound with M74 are displayed in Figure 9B. RMSF of the protein backbone indicated that nearly all residues of C-KIT showed less fluctuation through the MD simulation run. RoG values for the structural compactness of the simulated c-KIT protein backbone are displayed in Figure 11C. These demonstrated that with very little fluctuation, the c-KIT protein folded steadily in the presence of bound compound M74. Additionally, the protein–ligand contact map was monitored for the complex c-KIT protein–ligand M74 and revealed that amino acid residues Pro577, Lys581, Asn649, and Glu651 formed H-bond interactions during MD simulation, which were maintained for 80–90% of the span in the dynamic environment (Figure 11D). Asp615, Ala616, and Ala617 were found to form hydrophobic or water bridge interactions with compound M74. All intermolecular interactions were found to persist for approximately 40–50% of the simulation run. Overall, the MD simulation study analysis showed that the pyrazole derivative M74 can provide strong interaction stability with the c-KIT protein in dynamic conditions and hence possibly contribute towards designing and development of newer or potential inhibitors for c-Kit.

### 3.11. Binding Interaction Profile Analysis of HDAC–Pyrazole Derivative Complex

**Figure 12:**
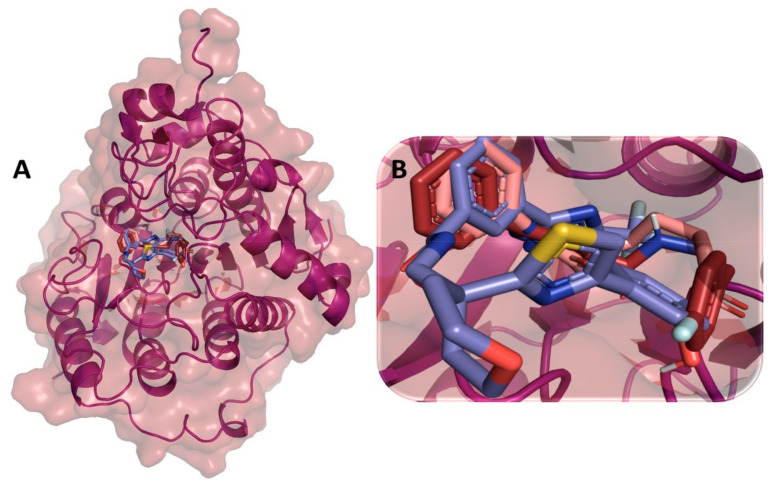

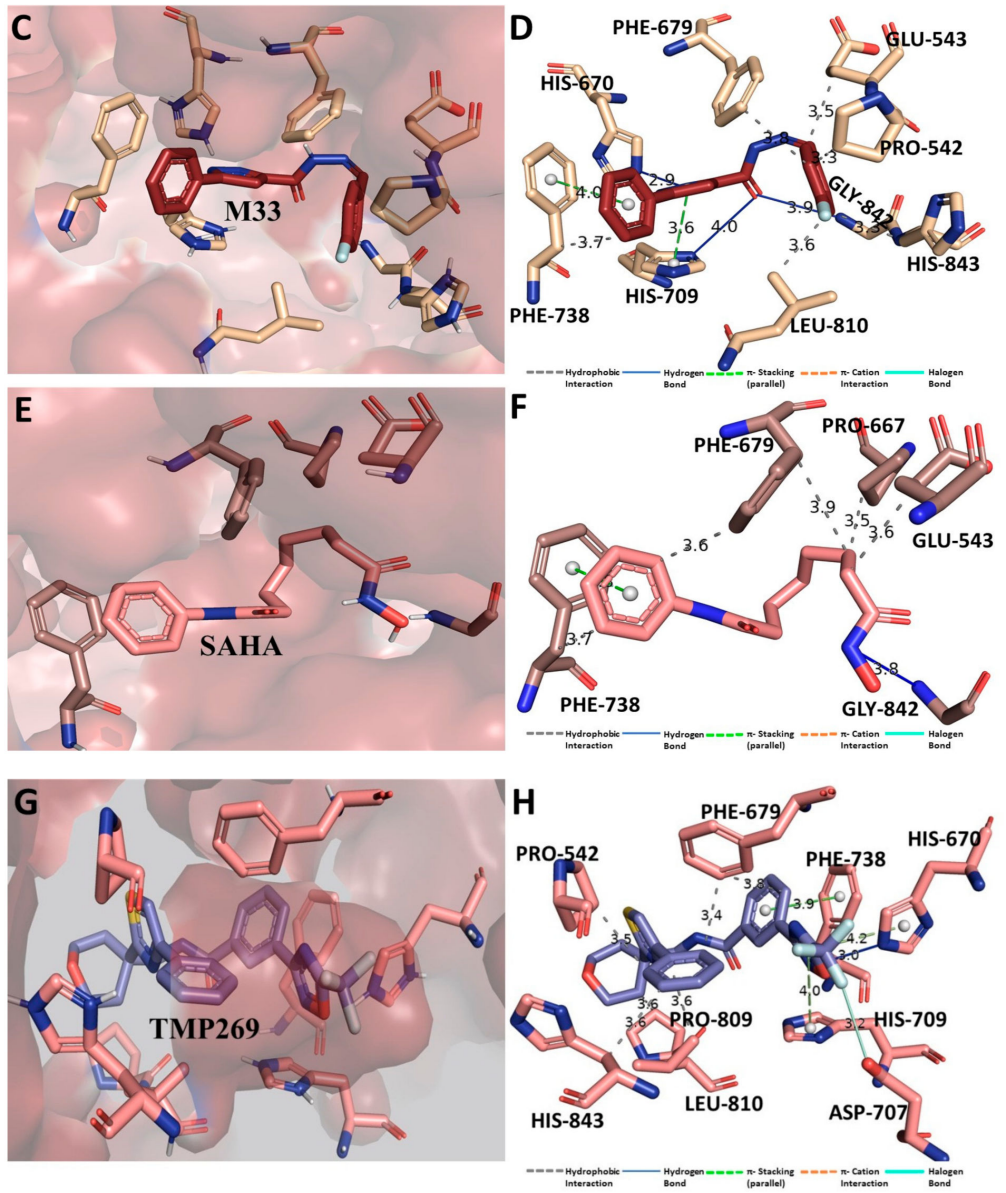
(**A**) Molecular docking-generated binding orientations of all standard compounds (SAHA; TMP269) and the proposed pyrazole inhibitor compound M33 in complex with HDAC7 (3ZNR) protein; (**B**) close-up binding interaction view exhibiting similar binding pattern of all the standard compounds including M33 at the active site cavity of HDAC7 (3ZNR) protein; (**C**) binding mode of the pyrazole derivative compound M33 in the active site cavity of HDAC7 (3ZNR) displayed in surface view; (**D**) molecular binding interaction and orientation of compound M33 with HDAC7 (3ZNR) protein; (**E**) binding mode of standard inhibitor (SAHA) in the active site cavity of HDAC7 (3ZNR) displayed in surface view; (**F**) molecular binding interaction and orientation of SAHA with HDAC7 (3ZNR) protein; (**G**) binding mode of standard compound TMP269 in active site cavity of HDAC7 (3ZNR) displayed in surface view; (**H**) molecular binding interaction and orientation of TMP269 with HDAC7 (3ZNR) protein.

Molecular docking analysis of the HDAC protein (PDB ID: 3ZNR) with all 63 pyrazole derivative compounds revealed that compound M33 was the most favorable compound with a binding affinity score of −10.1 kcal/mol. All 63 pyrazole compounds’ molecular docking-obtained binding affinity scores are shown in Table S6 (Supplementary Information). Docking of standard compound TMP269 yielded a binding affinity score of −10.3 kcal/mol, which is very close to the binding affinity score of M33 compound. Compound M33 showed an H-bond interaction with His670. Hydrophobic interactions were observed at the active site amino acid residues of HDAC: Pro542, Pro667, Phe679, Phe738, Pro809, and Leu810 of HDAC protein (Figure 12). It was quite interesting to observe a similar binding interaction map or network obtained for some phenacetyl and phenylbenzoyl hydroxamate compounds studied for HDAC inhibitory activity analyses [118]. 

### 3.12. Molecular Dynamic Simulation Analysis of HDAC–Pyrazole Derivative M33 Complex

**Figure 13:**
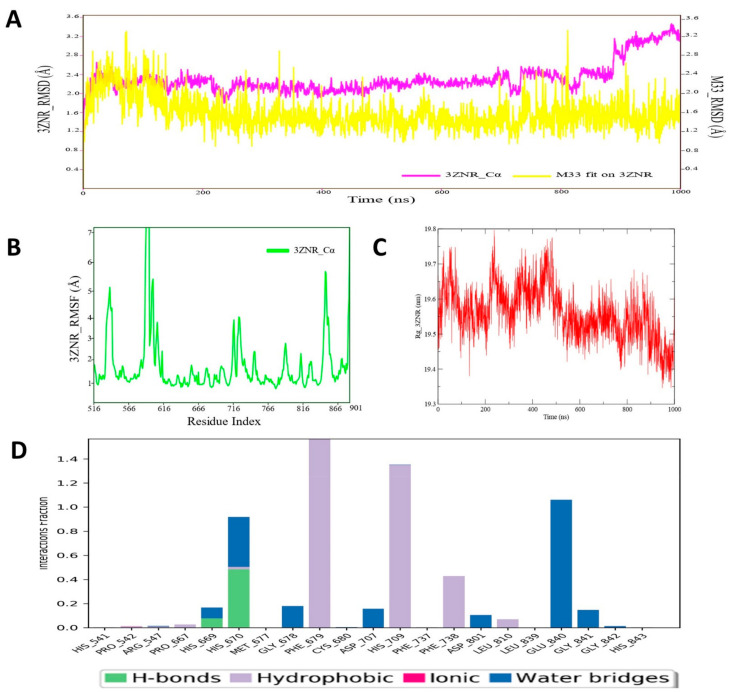
(**A**) RMSD profile of HDAC7 (3ZNR) protein backbone and compound M74 during 1000 ns simulation; (**B**) RMSF profile of c-KIT (6XVB) protein backbone during 1000 ns simulation; (**C**) RoG profile of c-KIT (6XVB) protein backbone during 1000 ns simulation; (**D**) illustration of the c-KIT (6XVB)–M74 contacts or interactions map monitored during 1000 ns simulation run.

MD simulation of HDAC–pyrazole derivative M33 complex was performed for 1000 ns, and upon successful competition, the RMSD, RMSF and RoG and protein–ligand contact map was analyzed. The RMSD plot is depicted in Figure 13A, revealing strong conformational stability for the both the ligand and M33 and HDAC protein backbone molecular system. For longer periods, the ligand RMSDs of M33 atom remained consistent, whereas protein backbone RMSD reflected a little oscillation at the end of the simulation run (~ at 820–1000 ns). The average RMSD value of HDAC protein backbone was 2.38 Å, whereas for compound M33 the it was estimated as 1.86 Å, which indicated substantial consistency and conformational stability of the molecular system during the MD simulation run. Assessment of RMSF values for the HDAC protein backbone atom of each amino acid residue showed that all residues maintained a stable conformation, except for a few residues that extended from ~586 to 622 and fluctuated slightly during simulation (Figure 13B). Such fluctuation was observed in the region of protein backbone, where no specific intermolecular interaction was recorded between M33 and HDAC protein. In contrast, few important active sites or crucial amino acid residues, such as Pro542, Pro667, Phe679, Phe738, Pro809, and Leu810 of HDAC protein, which formed different types of molecular interaction, exhibited less fluctuation during the entire MD simulation. To further investigate the structural rigidity or compactness of the simulated HDAC protein backbone, the RoG profile of the backbone atoms was measured and is displayed in Figure 13C. The RoG values demonstrated that although initially RoG values fluctuated a little bit, during the end of the simulation run, the RoG achieved a plateau state, indicating proper organization of structural backbone in dynamic conditions. A protein–ligand contacts map was also developed to study the actual dynamic interaction profile between M33 and HDAC protein (Figure 13D). The protein–ligand contacts map revealed an intriguing observation in terms of involvement of residue His670 for the creation of an H-bond interaction. Other residues, such as Phe679 and His709, were also found to form hydrophobic interactions. All such interactions remained intact during MD simulation for approximately 40–90% of the time in the dynamic environment. Apart from the abovementioned residues, Phe738, Glu840, and His670 established hydrophobic or water bridge interactions, etc. during MD simulation. Such interaction patterns also persisted for approximately 40–50% of the time span during the entire simulation run. Notably, MD simulation study analysis provided an insight that there were no sudden changes in HDAC protein upon binding of compound M33, indicating greater stability of the protein–ligand complex, which might be beneficial for designing and development of better potential inhibitors for HDAC protein.

### 3.13. ADME_T Prediction

Table 2 shows the different physicochemical and drug-likeness properties of the proposed pyrazole compounds. It was found that all compounds were significantly compliant with the recommended values of two well-known drug-likeness rules: Lipinski’s rule of five and Veber’s rule.

#### 3.13.1. Absorption Profile

Absorption profiles predicted for the top-ranked pyrazole compounds proposed as inhibitors/modulators for the studied target proteins are given in Table 3. For the rest of the pyrazole derivatives, results of absorption parameters are given in Table S7 of Supplementary Information. Estimated water solubility of compounds M33, M36, M72, M74, and M76 was −3.78, −4.472, −4.969, −4.813, and −4.527, respectively. For most of the proposed pyrazole compounds, the percentage of human intestinal absorption was also found to be very high, mostly >90%. Skin irritancy was checked using the skin permeability parameter prediction and was also found to be safe for most of the proposed compounds. CaCo2 permeability profile values were 0.91, 1.144, 0.953, 0.964, and 0.976 for the proposed compounds M33, M36, M72, M74, and M76, respectively. All proposed pyrazole compounds except M33 (inhibitor of Pgp II) were found to be potential inhibitor of Pgp I and II, and thereby can increase the oral bioavailability.

#### 3.13.2. Distribution Profile

Distribution parameters for all pyrazole compounds and results of such parameters, such as steady-state volume of distribution (VDss), fraction unbound (human), blood–brain barrier (logBB) and CNS permeability, are given in Table 4 for proposed compounds, and results of the rest compounds are given in Supplementary Information Table S9. The predicted VDss values were within the range of 0.08 to −0.02 L/kg, and categorized as low. Another important parameter, BBB, was 0.011, −0.046, −0.752, −0.996, and −0.587 for the proposed compounds M33, M36, M72, M74, and M76, respectively, indicating less probability to cross the BBB. Estimation of CNS permeability of all proposed compounds also highlighted less chance of passing or penetrating the CNS: −2.229, −2.502, −1.946, −2.113, and −1.986 for compounds M33, M36, M72, M74, and M76, respectively.

#### 3.13.3. Metabolism Profile

The expected metabolism profile of all proposed compounds was determined in terms of measuring the inhibition profile of different cytochrome (CYP2D6, CYP3A4, CYP1A2, CYP2C19, CYP2C9, CYP2D6, and CYP3A4) enzymes. Obtained results of metabolism profile of proposed compounds are given in Table 5. The predicted result of the rest of the pyrazole derivatives is given in Supplementary Information Table S10. It was found that all five compounds were expected inhibitors of CYP1A2 and CYP2C19. For enzyme CYP2C9, compound M33 was only found as a non-inhibitor, but predicted results all other four compounds (M36, M72, M74, and M76) found to be inhibitors for this enzyme class. Two other enzymes, CYP2D6 and CYP3A4, were indicated as non-inhibitors for all proposed compounds in the present study.

#### 3.13.4. Excretion Profile

Excretion profiles of all proposed compounds were determined using pkCSM, and results are presented in Table 6. Importantly, two parameters—renal organic cation transporter 2 (OCT2) and total drug clearance—were estimated under excretion profile. All compounds were non-inhibitors of OCT2, except compound M33. On the other hand, CLtot indicated the assessment of total clearance of drug in terms of combination of hepatic and renal clearance. The values of all proposed compounds were within the range of 0.03 to 0.80. The excretion predicted result of the rest of the pyrazole derivatives is given in Supplementary Information Table S11.

#### 3.13.5. Toxicity Profile

Various toxicity properties were predicted for all five compounds (M33, M36, M72, M74, and M76) and results are given in Table 7. None of the proposed compounds exhibited signs of AMES toxicity; hence, they can be classified as non-mutagenic. In addition, no indication of hERG I-inhibiting properties was revealed for any proposed compound. As per the predicted profile of hepatotoxicity indicators, compounds M33 and M36 were atoxic, whereas compounds M72, M74, and M76 were liver-toxic. No compounds showed indication of skin sensitization. The oral acute toxicity (LD_50_) of proposed compounds M33, M36, M72, M74, and M76 was 2.472, 2.276, 2.751, 2.776, and 2.615 mol/kg, respectively. For this toxicity parameter, the accepted or recommended value should be <2.5 mol/kg. On the other hand, oral chronic toxicity was also found within the recommended value (i.e., log 4.4.mg/toxicity;/day) for all proposed pyrazole compounds (Table 7). Another two important toxicity profiles, measured as *T. pyriformis* and minnow toxicity, were also low, as indicated by the values obtained. The toxicity predicted result of the rest of the pyrazole derivatives is given in Supplementary Information Table S12.

## 4. Conclusions

This comprehensive in silico study intended to identify the potential pyrazole derivatives as the inhibitors/modulators of six attractive therapeutic drug targets (CRMP2, C-RAF, CYP17, c-KIT, VEGFR, and HDAC) for different cancer types that have been extensively studied, including breast, liver, prostate, kidney, and stomach cancers. All these proteins were chosen based on their significant association with progression of oncogenesis or certain types of cancer development. Extensive binding interactions were studied through molecular docking for all 63 pyrazole derivatives in conjunction with six significant therapeutic target proteins. Initially, the best pyrazole derivatives were screened and selected for further study based on their binding affinity score against each protein target. Initial screening suggested that a majority of the identified pyrazole compounds (M33, M36, M72, and M76) have comparatively close binding affinity scores, as obtained for their respective standard drug compounds. The four pyrazole compounds M33, M36, M72, and M76 were identified as the best potential inhibitors or modulators for HDAC, C-RAF, CYP72 and VEGFR proteins, respectively. Interestingly, another pyrazole compound, M74, was found to be the best potential compound against two targeted proteins, i.e., CRMP2 and c-KIT exhibited much better binding affinity than the standard drug compounds studied. Molecular docking studies also suggested that all identified compounds prominently occupied the active site pocket of all the studied proteins. Based on the binding mode and intermolecular interaction profile of all the studied target proteins, it can be suggested that several types of biologically important or relevant molecular interactions (H-bond and hydrophobic contact) might definitely enhance the chances of stabilizing protein–ligand complex formation energetically. Amino acid residues Ser292, Lys293, and Lys297 of CRMP2, Ser357, Asn472, Asn486, Val363, Ala373, Leu406, and Phe475 of C-RAF, Ile112, Ala113, Phe132, Ile299, Ala302, Phe435, Leu361, Val366, Cys442, Ile443, Gly444, Leu447, and Ala448 of CYP17, Leu840, Val848, Ala866, Phe918, Leu1035, and Phe1047 of VEGFR, Pro542, His670, Phe679, His709, Phe738, Asp801, and Leu810 of HDAC, and Thr619, Glu605, Lys807, and Glu651 of c-KIT protein were found to be critically important residues for making biologically relevant interactions for exhibiting necessary inhibitory action. Moreover, to understand the dynamic characteristics, all selected compounds bound with respective target proteins underwent long-range 1000 ns MD simulation study. The MD simulation study also indicated that for longer periods, all identified pyrazole compounds remained in an energetically stable conformational state, which was reflected in the low protein backbone RMSD values obtained from each trajectory. Overall, the MD simulation study of all protein–ligand complexes demonstrated that all proteins in a compound-bound state achieved equilibration within a short time, indicating a stable conformation for both the proteins and identified ligand molecules. In sum, the present study has established the selectivity and potentiality profiles of a few pyrazole derivatives that can be further studied for modulation of targeted proteins and their signaling pathways for development of next-generation cancer therapeutics.

## Figures and Tables

**Table 1:** PDB IDs, atomic resolutions and number of residues of target proteins used in the current study.

Proteins	PDB IDs	References	Resolution (Å)	Residue’s Count
**CRMP2**	6JV9	[100]	2.26	540
**C-RAF**	3OMV	[101]	2.4	95
**CYP17**	4NKV	[52]	2.65	494
**VEGFR**	4AGD	[102]	2.81	353
**C-KIT**	6XVB	[103]	2.15	328
**HDAC**	3ZNR	[39]	2.4	423

**Table 2:** Predicted properties under Lipinski’s rule of five and Veber’s rule of selected pyrazole compounds.

Compounds	Lipinski Rule of 5					Veber’s Rule		
	MW	LogP	HBA	HBD	Lipinski’s Rule Violations	NRB	Surface Area	Vebers’ Violations
**M33**	308.316	2.5248	4	4	Suitable	1	131.811	Suitable
**M36**	366.424	3.8042	5	4	Suitable	1	162.702	Suitable
**M72**	380.864	3.6995	4	7	Suitable	1	159.911	Suitable
**M74**	441.314	3.5142	4	8	Suitable	2	168.269	Suitable
**M76**	360.446	3.35452	4	7	Suitable	1	155.973	Suitable

**MW** = molecular weight; **LogP** = lipophilicity; **HBA** = hydrogen bond acceptor; **HBD** = hydrogen bond donor; **NRB** = number of rotatable bonds.

**Table 3:** Various absorption parameters of the proposed pyrazole derivatives.

ABSORPTION							
Compounds	Water Solubility (Log mol/L)	CaCO_2_ Permeability (cm/s)	Intestinal Absorption (Human) in %	Skin Permeability	P-Glycoprotein Substrate	P-Glycoprotein II Inhibitor	P-Glycoprotein I Inhibitor
**M33**	−3.78	0.91	92.749	−2.774	−	+	−
**M36**	−4.472	1.144	94.034	−2.656	−	+	+
**M72**	−4.969	0.953	91.145	−2.878	−	+	+
**M74**	−4.813	0.964	88.182	−2.986	−	+	+
**M76**	−4.527	0.976	92.592	−2.889	−	+	+

**Table 4:** Distribution profile of the proposed pyrazole derivatives.

DITRIBUTION				
Molecules	VDss (Human) L kg^−1^	Fraction Unbound (Human)	BBB Permeability	CNS Permeability
**M33**	−0.021	0.053	0.011	−2.229
**M36**	0.088	0.008	−0.046	−2.502
**M72**	−0.365	0.035	−0.752	−1.946
**M74**	−0.623	0.058	−0.996	−2.113
**M76**	−0.342	0.050	−0.587	−1.986

**Table 5:** Estimated metabolism parameters for the proposed pyrazole compounds.

METABOLISM					
Molecules	CYP1A2 Inhibitor	CYP2C19 Inhibitor	CYP2C9 Inhibitor	CYP2D6 Inhibitor	CYP3A4 Inhibitor
**M33**	Inhibitor	Inhibitor	Non-Inhibitor	Non-Inhibitor	Non-Inhibitor
**M36**	Inhibitor	Inhibitor	Inhibitor	Non-Inhibitor	Non-Inhibitor
**M72**	Inhibitor	Inhibitor	Inhibitor	Non-Inhibitor	Non-Inhibitor
**M74**	Inhibitor	Inhibitor	Inhibitor	Non-Inhibitor	Non-Inhibitor
**M76**	Inhibitor	Inhibitor	Inhibitor	Non-Inhibitor	Non-Inhibitor

**Table 6:** Various excretion profiles of the proposed pyrazole compounds.

EXCRETION		
Molecules	Total Drug Clearance	Renal OCT2 Substrate Inhibitor
**M33**	0.543	Yes
**M36**	0.809	No
**M72**	0.322	No
**M74**	0.031	No
**M76**	0.276	No

**Table 7:** Predicted toxicity properties of the proposed pyrazole compounds.

TOXICITY										
Molecules	AMES Toxicity	Max. Tolerated Dose (Human)	hERG I Inhibitor	hERG II Inhibitor	Oral Rat Acute Toxicity (LD_50_)	Oral Rat Chronic Toxicity (LOAEL)	Hepatotoxicity	Skin Sensitization	*T.* *Pyriformis* Toxicity	Minnow Toxicity
**M33**	No	0.091	No	No	2.472	1.379	No	No	1.994	1.289
**M36**	No	0.225	No	Yes	2.276	2.261	No	No	0.894	0.054
**M72**	No	−0.127	No	Yes	2.751	1.531	Yes	No	1.207	0.478
**M74**	No	−0.142	No	Yes	2.776	1.556	Yes	No	0.898	0.748
**M76**	No	−0.119	No	Yes	2.615	1.649	Yes	No	1.253	0.768

## Supplementary Materials

The following supporting information can be downloaded at https://www.mdpi.com/article/10.3390/cimb44110361/s1.
