# Supplementary material for: In Silico Identification of Promising New Pyrazole Derivative-Based Small Molecules for Modulating CRMP2, C-RAF, CYP17, VEGFR, C-KIT, and HDAC—Application towards Cancer Therapeutics
Source: Curr Issues Mol Biol. 2022 Oct 31;44(11):5312–51. doi: 10.3390/cimb44110361 (PMC9689108; doi:10.3390/cimb44110361)
Supplement: Supplementary file 1 [file cimb-44-00361-s001.zip › cimb-1910348-supplementary.pdf]

Supplementary Information

In Silico Identification of Promising New Pyrazole Derivative-Based Small Molecules for Modulating CRMP2, C-RAF, CYP17, VEGFR, C-KIT, and HDAC—Application towards Cancer Therapeutics

Fatima Ezzahra Bennani [1,2,*], Khalid Karrouchi [3], Latifa Doudach [4], Mario Scrima [5,*], Noor Rahman [6], Luca Rastrelli [7], Trina Ekawati Tallei [8], Christopher E. Rudd [2,9,10,†], My El Abbes Faouzi [1,†] and M'hammed Ansar [11,†]

[1] Laboratory of Pharmacology and Toxicology, Bio Pharmaceutical and Toxicological Analysis Research Team, Faculty of Medicine and Pharmacy, Mohammed V University in Rabat, Rabat BP6203, Morocco
[2] Division of Immunology-Oncology, Centre de Recherche Hôpital Maisonneuve-Rosemont (CR-HMR), Montreal, QC H1T 2M4, Canada
[3] Laboratory of Analytical Chemistry, Faculty of Medicine and Pharmacy, Mohammed V University in Rabat, Rabat BP6203, Morocco
[4] Department of Biomedical Engineering Medical Physiology, Higher School of Technical Education of Rabat, Mohammed V University in Rabat, Rabat BP6203, Morocco
[5] Biogem Scarl, Via Camporeale, 83031 Ariano Irpino, AV, Italy
[6] Department of Biochemstry, Abdul Wali Khan University Mardan, Mardan 23200, Pakistan
[7] Dipartimento di Farmacia, University of Salerno, Via Giovanni Paolo II, 84084 Fisciano, SA, Italy
[8] Department of Biology, Faculty of Mathematics and Natural Sciences, Sam Ratulangi University, Manado 95115, North Sulawesi, Indonesia
[9] Department of Microbiology, Infection and Immunology, Faculty of Medicine, Université de Montreal, Montreal, QC H3T 1J4, Canada
[10] Division of Experimental Medicine, Department of Medicine, McGill University Health Center, McGill University, Montreal, QC H4A 3J1, Canada
[11] Laboratory of Medicinal Chemistry, Faculty of Medicine and Pharmacy, Mohammed V University in Rabat, Rabat BP6203, Morocco
* Correspondence: bennani.fatima.ezzahra@gmail.com (F.E.B.); marioscrima@gmail.com (M.S.)
† These authors contributed equally to this work.

Table S1. Molecular docking based binding affinity of all 63 pyrazoles derivatives with CRMP2 (PDB ID: 6JV9) protein.

	Protein-Pdb ID	Binding Affinity K/Mol
**All pyrazole derivatives series 63 Molecules**	CRMP2_6JV9__M47	-7
	CRMP2_6JV9__M74	-6,9
	CRMP2_6JV9__M33	-6,8
	CRMP2_6JV9__M72	-6,8
	CRMP2_6JV9__M77	-6,8
	CRMP2_6JV9__M79	-6,8
	CRMP2_6JV9__M25	-6,7
	CRMP2_6JV9__M76	-6,7
	CRMP2_6JV9__M78	-6,6
	CRMP2_6JV9__M73	-6,5
	CRMP2_6JV9__M69	-6,4
	CRMP2_6JV9__M28	-6,3
	CRMP2_6JV9__M36	-6,3
	CRMP2_6JV9__M71	-6,3
	CRMP2_6JV9__M24	-6,2
	CRMP2_6JV9__M31	-6,2
	CRMP2_6JV9__M27	-6,1
	CRMP2_6JV9__M34	-6,1
	CRMP2_6JV9__M22	-6
	CRMP2_6JV9__M26	-6
	CRMP2_6JV9__M29	-6
	CRMP2_6JV9__M32	-6
	CRMP2_6JV9__M75	-6
	CRMP2_6JV9__M83	-6
	CRMP2_6JV9__M21	-5,9
	CRMP2_6JV9__M23	-5,9
	CRMP2_6JV9__M42	-5,8
	CRMP2_6JV9__M67	-5,8
	CRMP2_6JV9__M59	-5,7
	CRMP2_6JV9__M63	-5,7
	CRMP2_6JV9__M64	-5,7
	CRMP2_6JV9__M54	-5,6
	CRMP2_6JV9__M13	-5,5
	CRMP2_6JV9__M7	-5,5
	CRMP2_6JV9__M12	-5,4
	CRMP2_6JV9__M18	-5,4
	CRMP2_6JV9__M10	-5,3

	CRMP2_6JV9__M11	-5,3
	CRMP2_6JV9__M15	-5,3
	CRMP2_6JV9__M16	-5,3
	CRMP2_6JV9__M17	-5,3
	CRMP2_6JV9__M19	-5,3
	CRMP2_6JV9__M20	-5,3
	CRMP2_6JV9__M8	-5,3
	CRMP2_6JV9__M9	-5,3
	CRMP2_6JV9__M14	-5,2
	CRMP2_6JV9__M82	-5,2
	CRMP2_6JV9__M39	-4,9
	CRMP2_6JV9__M41	-4,7
	CRMP2_6JV9__M48	-4,7
	CRMP2_6JV9__M86	-4,7
	CRMP2_6JV9__M44	-4,6
	CRMP2_6JV9__M53	-4,6
	CRMP2_6JV9__M56	-4,6
	CRMP2_6JV9__M37	-4,5
	CRMP2_6JV9__M43	-4,5
	CRMP2_6JV9__M50	-4,5
	CRMP2_6JV9__M40	-4,4
	CRMP2_6JV9__M38	-4,3
	CRMP2_6JV9__M45	-4,2
	CRMP2_6JV9__M46	-4,2
	CRMP2_6JV9__M49	-4,2
	CRMP2_6JV9__M3	-4,1
**Standars Activator**	CRMP2_6JV9_ nalidixic_acid	-5

Table S2. Molecular docking based binding affinity of all 63 pyrazoles derivatives with E-RAF (PDB ID:3OMV) protein

	**Protein-Pdb ID**	**Binding Affinity K/Mol**
**All pyrazole derivatives series 63 Molecules**	E-RAF_3OMV__M36	-9,7
	E-RAF_3OMV__M76	-9,6
	E-RAF_3OMV__M78	-9,6
	E-RAF_3OMV__M21	-9,5
	E-RAF_3OMV__M33	-9,4
	E-RAF_3OMV__M74	-9,3
	E-RAF_3OMV__M77	-9,3
	E-RAF_3OMV__M26	-9,2
	E-RAF_3OMV__M72	-9,2
	E-RAF_3OMV__M27	-9

	E-RAF_3OMV__M31	-9
	E-RAF_3OMV__M47	-9
	E-RAF_3OMV__M22	-8,9
	E-RAF_3OMV__M24	-8,9
	E-RAF_3OMV__M34	-8,9
	E-RAF_3OMV__M42	-8,9
	E-RAF_3OMV__M32	-8,8
	E-RAF_3OMV__M28	-8,7
	E-RAF_3OMV__M83	-8,7
	E-RAF_3OMV__M23	-8,5
	E-RAF_3OMV__M25	-8,5
	E-RAF_3OMV__M29	-8,5
	E-RAF_3OMV__M75	-8,5
	E-RAF_3OMV__M16	-8,4
	E-RAF_3OMV__M12	-8,3
	E-RAF_3OMV__M18	-8,3
	E-RAF_3OMV__M54	-8,3
	E-RAF_3OMV__M10	-8,2
	E-RAF_3OMV__M11	-8,2
	E-RAF_3OMV__M17	-8,2
	E-RAF_3OMV__M20	-8,2
	E-RAF_3OMV__M8	-8,2
	E-RAF_3OMV__M15	-8,1
	E-RAF_3OMV__M19	-8,1
	E-RAF_3OMV__M63	-8,1
	E-RAF_3OMV__M64	-8,1
	E-RAF_3OMV__M7	-8,1
	E-RAF_3OMV__M50	-8
	E-RAF_3OMV__M73	-8
	E-RAF_3OMV__M9	-8
	E-RAF_3OMV__M13	-7,9
	E-RAF_3OMV__M59	-7,8
	E-RAF_3OMV__M48	-7,7
	E-RAF_3OMV__M79	-7,7
	E-RAF_3OMV__M37	-7,6
	E-RAF_3OMV__M41	-7,6
	E-RAF_3OMV__M69	-7,6
	E-RAF_3OMV__M71	-7,6
	E-RAF_3OMV__M40	-7,5
	E-RAF_3OMV__M45	-7,5
	E-RAF_3OMV__M39	-7,4
	E-RAF_3OMV__M44	-7,4
	E-RAF_3OMV__M46	-7,2
	E-RAF_3OMV__M49	-7,2
	E-RAF_3OMV__M14	-7,1
	E-RAF_3OMV__M38	-7,1

	E-RAF_3OMV__M82	-7
	E-RAF_3OMV__M43	-6,9
	E-RAF_3OMV__M56	-6,7
	E-RAF_3OMV__M67	-6,4
	E-RAF_3OMV__M53	-5,9
	E-RAF_3OMV__M86	-5,8
	E-RAF_3OMV__M3	-5,1
**Standars Inhibitor**	E-RAF_3OMV__Sorafenib	-10,2

Table S3. Molecular docking based binding affinity of all 63 pyrazoles derivatives with CYP17 (PDB ID:4NKV) protein

	**Protein-Pdb ID**	**Binding Affinity K/Mol**
**All pyrazole derivatives series 63 Molecules**	CYP17_4NKV__M72	-10,4
	CYP17_4NKV__M74	-9,8
	CYP17_4NKV__M36	-9,7
	CYP17_4NKV__M73	-9,7
	CYP17_4NKV__M77	-9,6
	CYP17_4NKV__M78	-9,6
	CYP17_4NKV__M28	-9,5
	CYP17_4NKV__M33	-9,5
	CYP17_4NKV__M76	-9,5
	CYP17_4NKV__M71	-9,1
	CYP17_4NKV__M25	-9
	CYP17_4NKV__M26	-9
	CYP17_4NKV__M79	-9
	CYP17_4NKV__M83	-9
	CYP17_4NKV__M23	-8,8
	CYP17_4NKV__M27	-8,8
	CYP17_4NKV__M31	-8,8
	CYP17_4NKV__M24	-8,7
	CYP17_4NKV__M22	-8,6
	CYP17_4NKV__M64	-8,6
	CYP17_4NKV__M69	-8,6
	CYP17_4NKV__M32	-8,5
	CYP17_4NKV__M34	-8,5
	CYP17_4NKV__M63	-8,4
	CYP17_4NKV__M21	-8,3
	CYP17_4NKV__M29	-8,2
	CYP17_4NKV__M75	-8,2
	CYP17_4NKV__M40	-7,9
	CYP17_4NKV__M11	-7,8
	CYP17_4NKV__M59	-7,8
	CYP17_4NKV__M67	-7,7

	CYP17_4NKV__M12	-7,6
	CYP17_4NKV__M46	-7,6
	CYP17_4NKV__M48	-7,6
	CYP17_4NKV__M38	-7,5
	CYP17_4NKV__M42	-7,5
	CYP17_4NKV__M45	-7,5
	CYP17_4NKV__M54	-7,5
	CYP17_4NKV__M7	-7,5
	CYP17_4NKV__M13	-7,4
	CYP17_4NKV__M20	-7,4
	CYP17_4NKV__M43	-7,4
	CYP17_4NKV__M10	-7,3
	CYP17_4NKV__M15	-7,3
	CYP17_4NKV__M16	-7,3
	CYP17_4NKV__M18	-7,3
	CYP17_4NKV__M37	-7,3
	CYP17_4NKV__M41	-7,3
	CYP17_4NKV__M49	-7,1
	CYP17_4NKV__M50	-7,1
	CYP17_4NKV__M8	-7,1
	CYP17_4NKV__M17	-7
	CYP17_4NKV__M39	-7
	CYP17_4NKV__M9	-7
	CYP17_4NKV__M14	-6,9
	CYP17_4NKV__M19	-6,9
	CYP17_4NKV__M82	-6,9
	CYP17_4NKV__M44	-6,8
	CYP17_4NKV__M56	-6
	CYP17_4NKV__M53	-5,6
	CYP17_4NKV__M86	-5,6
	CYP17_4NKV__M3	-5,1
	CYP17_4NKV__M47	-3,7
**Standars Inhibitor**	CYP17_4NKV__Galeterone	-11,6
	CYP17_4NKV__Abiraterone_acetate	-9,7
	CYP17_4NKV__Orteronel	-8,7

Table S4. Molecular docking based binding affinity of all 63 pyrazoles derivatives with VEGFR (PDB ID:4AGD) protein

	Protein-Pdb ID	Binding Affinity K/Mol
All pyrazole derivatives series 63 Molecules	VEGFR_4AGD__M72	-9,2
	VEGFR_4AGD__M76	-9,2
	VEGFR_4AGD__M25	-9,1
	VEGFR_4AGD__M78	-9,1
	VEGFR_4AGD__M26	-9
	VEGFR_4AGD__M27	-9
	VEGFR_4AGD__M73	-9
	VEGFR_4AGD__M33	-8,9
	VEGFR_4AGD__M77	-8,9
	VEGFR_4AGD__M83	-8,9
	VEGFR_4AGD__M22	-8,8
	VEGFR_4AGD__M31	-8,8
	VEGFR_4AGD__M32	-8,8
	VEGFR_4AGD__M34	-8,8
	VEGFR_4AGD__M69	-8,8
	VEGFR_4AGD__M74	-8,8
	VEGFR_4AGD__M54	-8,7
	VEGFR_4AGD__M23	-8,6
	VEGFR_4AGD__M28	-8,6
	VEGFR_4AGD__M36	-8,6
	VEGFR_4AGD__M24	-8,5
	VEGFR_4AGD__M29	-8,5
	VEGFR_4AGD__M64	-8,4
	VEGFR_4AGD__M79	-8,2
	VEGFR_4AGD__M20	-8,1
	VEGFR_4AGD__M42	-8
	VEGFR_4AGD__M75	-8
	VEGFR_4AGD__M59	-7,9
	VEGFR_4AGD__M63	-7,9
	VEGFR_4AGD__M71	-7,9
	VEGFR_4AGD__M47	-7,9
	VEGFR_4AGD__M10	-7,7
	VEGFR_4AGD__M12	-7,7
	VEGFR_4AGD__M13	-7,7
	VEGFR_4AGD__M16	-7,7
	VEGFR_4AGD__M18	-7,7
	VEGFR_4AGD__M21	-7,7
	VEGFR_4AGD__M11	-7,6
	VEGFR_4AGD__M45	-7,6
	VEGFR_4AGD__M9	-7,5

	VEGFR_4AGD__M17	-7,4
	VEGFR_4AGD__M19	-7,4
	VEGFR_4AGD__M39	-7,4
	VEGFR_4AGD__M40	-7,4
	VEGFR_4AGD__M46	-7,4
	VEGFR_4AGD__M7	-7,4
	VEGFR_4AGD__M82	-7,4
	VEGFR_4AGD__M15	-7,3
	VEGFR_4AGD__M37	-7,2
	VEGFR_4AGD__M8	-7,2
	VEGFR_4AGD__M49	-7,1
	VEGFR_4AGD__M38	-7
	VEGFR_4AGD__M14	-6,9
	VEGFR_4AGD__M50	-6,9
	VEGFR_4AGD__M41	-6,8
	VEGFR_4AGD__M53	-6,6
	VEGFR_4AGD__M48	-6,5
	VEGFR_4AGD__M67	-6,4
	VEGFR_4AGD__M86	-6,4
	VEGFR_4AGD__M43	-6,3
	VEGFR_4AGD__M44	-6,2
	VEGFR_4AGD__M56	-6,1
	VEGFR_4AGD__M3	-5,6
**Standars Inhibitor**	protein2\r_Sunitinib	-10
	Sorafenib mst be dock	

Table S5. Molecular docking based binding affinity of all 63 pyrazoles derivatives with C-KIT (PDB ID:6XVB) protein

	Protein-Pdb ID	Binding Affinity K/Mol
**All pyrazole derivatives series 63 Molecules**	C-KIT_6XVB_M74	-9,2
	C-KIT_6XVB_M72	-9,1
	C-KIT_6XVB_M75	-9,1
	C-KIT_6XVB_M76	-9,1
	C-KIT_6XVB_M78	-9,1
	C-KIT_6XVB_M77	-8,9
	C-KIT_6XVB_M27	-8,8
	C-KIT_6XVB_M69	-8,8
	C-KIT_6XVB_M26	-8,7
	C-KIT_6XVB_M31	-8,7
	C-KIT_6XVB_M32	-8,7
	C-KIT_6XVB_M34	-8,7
	C-KIT_6XVB_M25	-8,6
	C-KIT_6XVB_M47	-8,6

	C-KIT_6XVB_M71	-8,6
	C-KIT_6XVB_M22	-8,5
	C-KIT_6XVB_M28	-8,5
	C-KIT_6XVB_M79	-8,5
	C-KIT_6XVB_M23	-8,4
	C-KIT_6XVB_M33	-8,4
	C-KIT_6XVB_M29	-8,3
	C-KIT_6XVB_M83	-8,3
	C-KIT_6XVB_M36	-8,2
	C-KIT_6XVB_M73	-8,1
	C-KIT_6XVB_M40	-8
	C-KIT_6XVB_M24	-7,9
	C-KIT_6XVB_M63	-7,9
	C-KIT_6XVB_M21	-7,8
	C-KIT_6XVB_M64	-7,8
	C-KIT_6XVB_M67	-7,8
	C-KIT_6XVB_M39	-7,7
	C-KIT_6XVB_M48	-7,7
	C-KIT_6XVB_M20	-7,6
	C-KIT_6XVB_M38	-7,6
	C-KIT_6XVB_M41	-7,6
	C-KIT_6XVB_M43	-7,6
	C-KIT_6XVB_M7	-7,6
	C-KIT_6XVB_M11	-7,5
	C-KIT_6XVB_M13	-7,5
	C-KIT_6XVB_M16	-7,5
	C-KIT_6XVB_M45	-7,5
	C-KIT_6XVB_M54	-7,5
	C-KIT_6XVB_M59	-7,5
	C-KIT_6XVB_M10	-7,4
	C-KIT_6XVB_M15	-7,4
	C-KIT_6XVB_M18	-7,4
	C-KIT_6XVB_M42	-7,4
	C-KIT_6XVB_M46	-7,4
	C-KIT_6XVB_M49	-7,4
	C-KIT_6XVB_M12	-7,3
	C-KIT_6XVB_M17	-7,3
	C-KIT_6XVB_M37	-7,3
	C-KIT_6XVB_M19	-7,2
	C-KIT_6XVB_M9	-7,2
	C-KIT_6XVB_M44	-7,1
	C-KIT_6XVB_M8	-7,1
	C-KIT_6XVB_M14	-6,9
	C-KIT_6XVB_M82	-6,9
	C-KIT_6XVB_M50	-6,4
	C-KIT_6XVB_M56	-6,1

	C-KIT_6XVB_M86	-5,9
	C-KIT_6XVB_M53	-5,8
	C-KIT_6XVB_M3	-5,3
**Standars Inhibitor**	protein4\r_Sunitinib	-7,9
	Sorafenib mst be dock	

Table S6. Molecular docking based binding affinity of all 63 pyrazoles derivatives with HDAC (PDB ID:3ZNR) protein

	**Protein-Pdb ID**	**Binding Affinity K/Mol**
**All pyrazole derivatives series 63 Molecules**	HDAC_3ZNR_M33	-10,1
	HDAC_3ZNR_M83	-9,7
	HDAC_3ZNR_M25	-9,6
	HDAC_3ZNR_M23	-9,4
	HDAC_3ZNR_M21	-9
	HDAC_3ZNR_M40	-8,8
	HDAC_3ZNR_M72	-8,8
	HDAC_3ZNR_M28	-8,7
	HDAC_3ZNR_M36	-8,7
	HDAC_3ZNR_M74	-8,6
	HDAC_3ZNR_M76	-8,5
	HDAC_3ZNR_M78	-8,5
	HDAC_3ZNR_M77	-8,4
	HDAC_3ZNR_M79	-8,4
	HDAC_3ZNR_M82	-8,4
	HDAC_3ZNR_M63	-8,3
	HDAC_3ZNR_M64	-8,2
	HDAC_3ZNR_M69	-8,1
	HDAC_3ZNR_M73	-8,1
	HDAC_3ZNR_M24	-8
	HDAC_3ZNR_M48	-8
	HDAC_3ZNR_M50	-8
	HDAC_3ZNR_M54	-8
	HDAC_3ZNR_M59	-8
	HDAC_3ZNR_M41	-7,9
	HDAC_3ZNR_M43	-7,9
	HDAC_3ZNR_M45	-7,9
	HDAC_3ZNR_M38	-7,8
	HDAC_3ZNR_M46	-7,8
	HDAC_3ZNR_M37	-7,7
	HDAC_3ZNR_M39	-7,7
	HDAC_3ZNR_M42	-7,7
	HDAC_3ZNR_M49	-7,7

	HDAC_3ZNR_M71	-7,7
	HDAC_3ZNR_M75	-7,7
	HDAC_3ZNR_M13	-7,6
	HDAC_3ZNR_M20	-7,6
	HDAC_3ZNR_M26	-7,6
	HDAC_3ZNR_M44	-7,5
	HDAC_3ZNR_M47	-7,5
	HDAC_3ZNR_M67	-7,5
	HDAC_3ZNR_M11	-7,4
	HDAC_3ZNR_M10	-7,3
	HDAC_3ZNR_M22	-7,3
	HDAC_3ZNR_M15	-7,2
	HDAC_3ZNR_M16	-7,2
	HDAC_3ZNR_M17	-7,2
	HDAC_3ZNR_M18	-7,2
	HDAC_3ZNR_M19	-7,2
	HDAC_3ZNR_M27	-7,2
	HDAC_3ZNR_M7	-7,2
	HDAC_3ZNR_M29	-7,1
	HDAC_3ZNR_M9	-7,1
	HDAC_3ZNR_M12	-7
	HDAC_3ZNR_M31	-7
	HDAC_3ZNR_M8	-7
	HDAC_3ZNR_M32	-6,9
	HDAC_3ZNR_M34	-6,9
	HDAC_3ZNR_M53	-6,7
	HDAC_3ZNR_M56	-6,6
	HDAC_3ZNR_M86	-6,6
	HDAC_3ZNR_M14	-6,5
	HDAC_3ZNR_M3	-5,9
**Standars Inhibitor**	HDAC_3ZNR_SAHA	-7,6
	HDAC_3ZNR_TMP269	-10,3

Table S7: *In Silico* predicted Lipinski rule of five and veer's rule of all pyrazole compounds.

Molecule	Lipinski rule of 5					Veber's Rule		
	MW	LogP	HBA	HBD	Lipinski's violations	NRB	Surface area	Veber's violations
M3	140.146	-0.8031	4	2	Suitable	1	57.975	Suitable
M7	228.255	1.3573	3	4	Suitable	1	98.953	Suitable
M8	258.281	1.3659	4	5	Suitable	1	110.431	Suitable
M9	271.324	1.4233	4	5	Suitable	1	117.443	Suitable
M10	273.252	1.2655	4	6	Suitable	1	113.606	Suitable

M11	262.7	2.0107	3	4	Suitable	1	109.256	Suitable
M12	297.145	2.6641	3	4	Suitable	1	119.559	Suitable
M13	323.15	1.8254	3	5	Suitable	2	117.615	Suitable
M14	218.216	0.9503	3	5	Suitable	1	91.747	Suitable
M15	274.28	1.0715	6	2	Suitable	4	115.226	Suitable
M16	242.282	1.66572	3	4	Suitable	1	105.318	Suitable
M17	262.7	2.0107	3	4	Suitable	1	109.256	Suitable
M18	246.245	1.4964	3	4	Suitable	1	103.118	Suitable
M19	307.151	2.1198	3	4	Suitable	1	112.82	Suitable
M20	242.282	1.7474	3	4	Suitable	1	105.318	Suitable
M21	304.353	2.7758	4	4	Suitable	1	134.01	Suitable
M22	290.326	2.3857	4	4	Suitable	1	127.645	Suitable
M23	320.352	2.3943	5	5	Suitable	1	139.123	Suitable
M24	333.395	2.4517	5	5	Suitable	1	146.135	Suitable
M25	335.323	2.2939	5	6	Suitable	1	142.298	Suitable
M26	324.771	3.0391	4	4	Suitable	1	137.948	Suitable
M27	359.216	3.6925	4	4	Suitable	1	148.252	Suitable
M28	385.221	2.8538	4	5	Suitable	2	146.307	Suitable
M29	280.287	1.9787	4	5	Suitable	1	120.439	Suitable
M31	304.353	2.69412	4	4	Suitable	1	134.01	Suitable
M32	324.771	3.0391	4	4	Suitable	1	137.948	Suitable
M33	308.316	2.5248	4	4	Suitable	1	131.811	Suitable
M34	369.222	3.1482	4	4	Suitable	1	141.513	Suitable
M36	366.424	3.8042	5	4	Suitable	1	162.702	Suitable
M37	256.309	1.65024	4	4	Suitable	1	111.497	Suitable
M38	286.335	1.65884	5	5	Suitable	1	122.976	Suitable
M39	299.378	1.71624	5	5	Suitable	1	129.987	Suitable
M40	301.306	1.55844	5	6	Suitable	1	126.15	Suitable
M41	290.754	2.30364	4	4	Suitable	1	121.801	Suitable
M42	325.199	2.95704	4	4	Suitable	1	132.104	Suitable
M43	351.204	2.11834	4	5	Suitable	2	130.159	Suitable
M44	246.27	1.24324	4	5	Suitable	1	104.292	Suitable
M45	270.336	1.95866	4	4	Suitable	1	117.862	Suitable
M46	290.754	2.30364	4	4	Suitable	1	121.801	Suitable
M47	274.299	1.78934	4	4	Suitable	1	115.663	Suitable
M48	302.334	1.36444	5	6	Suitable	2	127.77	Suitable
M49	335.205	2.41274	4	4	Suitable	1	125.365	Suitable
M50	270.336	2.04034	4	4	Suitable	1	117.862	Suitable
M53	196.239	-0.1507	1	7	Suitable	2	79.938	Suitable
M54	258.31	0.8777	2	7	Suitable	2	108.63	Suitable

M56	224.293	0.14224	2	7	Suitable	2	92.483	Suitable
M59	327.417	2.0837	4	8	Suitable	1	139.405	Suitable
M63	379.243	2.4858	3	8	Suitable	2	139.577	Suitable
M64	298.375	2.32612	3	7	Suitable	1	127.281	Suitable
M67	330.373	1.7319	4	9	Suitable	2	137.188	Suitable
M69	346.419	3.0461	4	7	Suitable	1	149.608	Suitable
M71	389.488	3.1121	5	8	Suitable	1	168.097	Suitable
M72	380.864	3.6995	4	7	Suitable	1	159.911	Suitable
M73	415.309	4.3529	4	7	Suitable	1	170.214	Suitable
M74	441.314	3.5142	4	8	Suitable	2	168.269	Suitable
M75	336.38	2.6391	4	8	Suitable	1	142.402	Suitable
M76	360.446	3.35452	4	7	Suitable	1	155.973	Suitable
M77	380.864	3.6995	4	7	Suitable	1	159.911	Suitable
M78	364.409	3.1852	4	7	Suitable	1	153.773	Suitable
M79	425.315	3.8086	4	7	Suitable	1	163.475	Suitable
M82	241.254	1.1293	2	5	Suitable	1	102.692	Suitable
M83	303.325	2.1577	3	5	Suitable	1	131.384	Suitable
M86	182.208	0.927	1	6	Suitable	1	74.003	Suitable

MW = molecular weight; LogP = lipohilicity; HBA = hydrogen bond acceptor

HBD = hydrogen bond donor; NRB = number of rotatable bonds

Table S8: Predicted absorption profile of all pyrazole derivatives

ABSORPTION							
**Molecule**	**Water solubility**	**Caco2 permeability**	**Intestinal absorption (human)**	**Skin Permeability**	**P-glycoprotein substrate**	**P-glycoprotein I inhibitor**	**P-glycoprotein II inhibitor**
M3	-0.954	0.59	83.582	-4.22	-	-	-
M7	-2.286	0.787	94.084	-2.722	-	-	-
M8	-2.884	0.564	94.428	-3.146	-	-	-
M9	-2.989	0.67	94.665	-3.135	-	-	-
M10	-3.049	-0.073	83.589	-2.625	-	-	-
M11	-3.214	0.833	92.607	-2.983	-	-	-
M12	-4.146	0.864	90.946	-2.67	-	-	-
M13	-3.497	0.989	90.823	-3.338	-	-	-
M14	-2.592	0.753	90.184	-3.628	-	-	-
M15	-3.193	0.039	77.005	-3.534	-	-	-
M16	-2.891	0.843	94.065	-2.738	-	-	-
M17	-3.119	0.843	92.423	-2.662	-	-	-
M18	-2.399	0.794	93.325	-2.853	-	-	-
M19	-3.256	0.838	92.356	-2.654	-	-	-
M20	-2.758	0.851	93.879	-2.998	-	-	-

M21	-4.37	1.09	94.932	-2.975	-	+	-
M22	-3.334	0.904	93.307	-2.545	-	-	-
M23	-3.87	0.957	93.668	-2.832	-	+	-
M24	-4.013	0.948	93.905	-2.764	+	+	-
M25	-3.717	-0.077	89.226	-2.731	-	+	-
M26	-3.942	0.886	91.647	-2.56	-	+	-
M27	-5.004	0.877	90.186	-2.711	+	+	-
M28	-4.053	0.908	88.895	-2.939	+	+	-
M29	-3.415	0.975	94.474	-2.778	-	-	-
M31	-3.868	0.916	93.305	-2.688	-	+	-
M32	-4.3	0.895	91.847	-2.699	-	+	-
M33	-3.78	0.91	92.749	-2.774	-	+	-
M34	-4.421	0.89	91.78	-2.697	-	+	-
M36	-4.472	1.144	94.034	-2.656	-	+	+
M37	-3.777	1.266	96.423	-3.001	-	-	-
M38	-3.34	1.402	95.44	-2.605	-	-	-
M39	-4.394	0.914	96.821	-3.041	-	-	-
M40	-4.65	0.013	83.785	-2.873	-	-	-
M41	-4.513	1.312	94.762	-2.967	-	-	-
M42	-5.21	1.357	93.101	-2.941	-	-	-
M43	-4.573	1.059	91.81	-3.282	-	-	-
M44	-3.781	0.841	95.591	-3.3	-	-	-
M45	-3.514	1.308	95.077	-2.586	-	-	-
M46	-4.513	1.312	94.762	-2.967	-	-	-
M47	-4.029	1.279	95.664	-3.101	-	-	-
M48	-3.14	1.411	94.581	-3.02	+	-	-
M49	-4.621	1.317	94.695	-2.955	-	-	-
M50	-4.078	1.289	96.034	-2.956	-	-	-
M53	-2.962	0.644	100	-2.899	-	-	-
M54	-2.919	0.645	71.418	-3.186	-	-	-
M56	-2.99	1.216	100	-2.748	-	-	-
M59	-4.09	1.006	94.136	-2.882	-	-	-
M63	-3.749	1.257	91.928	-3.049	+	-	-
M64	-3.892	0.982	93.462	-2.856	-	-	-
M67	-4.238	-0.14	83.813	-3.272	+	-	-
M69	-4.885	1.185	94.088	-2.864	-	+	-
M71	-4.686	1.007	93.193	-2.936	-	+	+
M72	-4.969	0.953	91.145	-2.878	-	+	+
M73	-5.526	0.548	89.473	-2.868	-	+	+
M74	-4.813	0.964	88.182	-2.986	-	+	+
M75	-4.841	1.108	93.828	-2.969	-	-	-
M76	-4.527	0.976	92.592	-2.889	-	+	+
M77	-4.931	0.954	91.134	-2.877	-	+	+
M78	-4.494	0.969	92.036	-2.931	-	+	+
M79	-5.029	0.95	91.067	-2.877	-	+	+
M82	-2.477	1.035	70.439	-2.875	-	-	-

M83	-3.347	0.786	93.58	-2.736	+	-	-
M86	-3.094	0.654	100	-3.25	-	-	-

Table S9: Predicted distribution profile of all pyrazole derivatives

DITRIBUTION				
**Molecules**	**VDss (human)** L kg-1	**Fraction unbound (human)**	**BBB permeability**	**CNS permeability**
M3	-0.342	0.772	-0.17	-3.476
M7	0.052	0.364	-0.178	-2.439
M8	-0.045	0.34	-0.412	-2.64
M9	0.014	0.328	-0.22	-2.54
M10	0.046	0.206	-0.728	-2.675
M11	-0.007	0.313	-0.054	-2.335
M12	0.059	0.298	-0.036	-2.218
M13	-0.077	0.379	-0.431	-2.992
M14	-0.198	0.595	-0.373	-3.04
M15	-0.25	0.439	-0.527	-3.02
M16	0.144	0.36	-0.022	-2.374
M17	0.026	0.32	-0.067	-2.325
M18	-0.121	0.36	-0.168	-2.478
M19	0.037	0.312	-0.069	-2.302
M20	0.054	0.327	-0.04	-2.358
M21	-0.16	0.167	-0.097	-2.655
M22	0.292	0.123	0.011	-2.178
M23	-0.013	0.013	-0.344	-2.382
M24	0.181	0.037	-0.28	-2.281
M25	0.162	0.017	-0.655	-2.412
M26	0.276	0.089	0.011	-2.064
M27	0.127	0	0.017	-1.961
M28	-0.072	0.048	-0.55	-2.243
M29	0.074	0.339	-0.335	-2.854
M31	0.198	0.038	0.031	-2.116
M32	0.142	0.022	0.018	-2.075
M33	-0.021	0.053	0.011	-2.229
M34	0.157	0.016	0.016	-2.053
M36	0.088	0.008	-0.046	-2.502
M37	-0.108	0.416	0.018	-2.804
M38	-0.221	0.183	-0.49	-2.71
M39	-0.125	0.384	-0.029	-2.825
M40	-0.463	0.319	-0.7	-2.892
M41	-0.139	0.371	0.017	-2.809
M42	-0.169	0.325	0.016	-2.814
M43	-0.345	0.39	-0.573	-2.913
M44	-0.201	0.57	-0.357	-2.963

M45	0	0.212	0.271	-2.444
M46	-0.139	0.371	0.017	-2.809
M47	-0.275	0.409	0.01	-2.84
M48	-0.09	0.285	-0.542	-3.032
M49	-0.128	0.362	0.016	-2.807
M50	-0.076	0.387	0.031	-2.785
M53	0.018	0.756	-0.043	-3.501
M54	-0.195	0.388	-0.555	-2.964
M56	0.336	0.593	-0.1	-3.482
M59	0.049	0.18	-0.732	-2.535
M63	0.24	0.233	-0.807	-3.05
M64	0.199	0.216	-0.557	-2.37
M67	-0.729	0.382	-1.027	-3.015
M69	-0.419	0.059	-0.591	-2.711
M71	-0.393	0.049	-0.744	-2.151
M72	-0.365	0.035	-0.752	-1.946
M73	-0.422	0	-0.931	-1.831
M74	-0.623	0.058	-0.996	-2.113
M75	-0.522	0.181	-0.776	-2.864
M76	-0.342	0.05	-0.587	-1.986
M77	-0.399	0.034	-0.762	-1.946
M78	-0.549	0.068	-0.795	-2.099
M79	-0.387	0.027	-0.771	-1.923
M82	0.018	0.23	-0.953	-2.617
M83	-0.454	0.163	-0.998	-2.394
M86	0.014	0.659	-0.395	-3.036

Table S10: Predicted metabolism profile of all pyrazole derviatives

**METABOLISM**					
**Molecule**	**CYP1A2 inhibitor**	**CYP2C19 inhibitor**	**CYP2C9 inhibitor**	**CYP2D6 inhibitor**	**CYP3A4 inhibitor**
M3	Non-Inhibitor	Non-Inhibitor	Non-Inhibitor	Non-Inhibitor	Non-Inhibitor
M7	Inhibitor	Non-Inhibitor	Non-Inhibitor	Non-Inhibitor	Non-Inhibitor
M8	Inhibitor	Non-Inhibitor	Non-Inhibitor	Non-Inhibitor	Non-Inhibitor
M9	Non-Inhibitor	Non-Inhibitor	Non-Inhibitor	Non-Inhibitor	Non-Inhibitor
M10	Non-Inhibitor	Non-Inhibitor	Non-Inhibitor	Non-Inhibitor	Non-Inhibitor
M11	Inhibitor	Non-Inhibitor	Non-Inhibitor	Non-Inhibitor	Non-Inhibitor
M12	Inhibitor	Non-Inhibitor	Non-Inhibitor	Non-Inhibitor	Non-Inhibitor
M13	Inhibitor	Non-Inhibitor	Non-Inhibitor	Non-Inhibitor	Non-Inhibitor
M14	Non-	Non-	Non-	Non-	Non-

	Inhibitor	Inhibitor	Inhibitor	Inhibitor	Inhibitor
M15	Non-Inhibitor	Non-Inhibitor	Non-Inhibitor	Non-Inhibitor	Non-Inhibitor
M16	Inhibitor	Non-Inhibitor	Non-Inhibitor	Non-Inhibitor	Non-Inhibitor
M17	Inhibitor	Non-Inhibitor	Non-Inhibitor	Non-Inhibitor	Non-Inhibitor
M18	Inhibitor	Non-Inhibitor	Non-Inhibitor	Non-Inhibitor	Non-Inhibitor
M19	Inhibitor	Non-Inhibitor	Non-Inhibitor	Non-Inhibitor	Non-Inhibitor
M20	Inhibitor	Non-Inhibitor	Non-Inhibitor	Non-Inhibitor	Non-Inhibitor
M21	Inhibitor	Inhibitor	Non-Inhibitor	Non-Inhibitor	Non-Inhibitor
M22	Inhibitor	Inhibitor	Non-Inhibitor	Non-Inhibitor	Non-Inhibitor
M23	Inhibitor	Inhibitor	Inhibitor	Non-Inhibitor	Non-Inhibitor
M24	Inhibitor	Inhibitor	Inhibitor	Non-Inhibitor	Non-Inhibitor
M25	Non-Inhibitor	Non-Inhibitor	Non-Inhibitor	Non-Inhibitor	Non-Inhibitor
M26	Inhibitor	Inhibitor	Inhibitor	Non-Inhibitor	Non-Inhibitor
M27	Inhibitor	Inhibitor	Inhibitor	Non-Inhibitor	Non-Inhibitor
M28	Inhibitor	Inhibitor	Non-Inhibitor	Non-Inhibitor	Non-Inhibitor
M29	Inhibitor	Non-Inhibitor	Non-Inhibitor	Non-Inhibitor	Non-Inhibitor
M31	Inhibitor	Inhibitor	Non-Inhibitor	Non-Inhibitor	Non-Inhibitor
M32	Inhibitor	Inhibitor	Inhibitor	Non-Inhibitor	Non-Inhibitor
M33	Inhibitor	Inhibitor	Non-Inhibitor	Non-Inhibitor	Non-Inhibitor
M34	Inhibitor	Inhibitor	Inhibitor	Non-Inhibitor	Non-Inhibitor
M36	Inhibitor	Inhibitor	Inhibitor	Non-Inhibitor	Non-Inhibitor
M37	Non-Inhibitor	Non-Inhibitor	Non-Inhibitor	Non-Inhibitor	Non-Inhibitor
M38	Non-Inhibitor	Non-Inhibitor	Non-Inhibitor	Non-Inhibitor	Non-Inhibitor
M39	Non-Inhibitor	Non-Inhibitor	Non-Inhibitor	Non-Inhibitor	Non-Inhibitor
M40	Non-Inhibitor	Non-Inhibitor	Non-Inhibitor	Non-Inhibitor	Non-Inhibitor
M41	Inhibitor	Inhibitor	Non-Inhibitor	Non-Inhibitor	Non-Inhibitor
M42	Inhibitor	Inhibitor	Inhibitor	Non-Inhibitor	Non-Inhibitor
M43	Inhibitor	Non-Inhibitor	Non-Inhibitor	Non-Inhibitor	Non-Inhibitor

M44	Non-Inhibitor	Non-Inhibitor	Non-Inhibitor	Non-Inhibitor	Non-Inhibitor
M45	Non-Inhibitor	Non-Inhibitor	Non-Inhibitor	Non-Inhibitor	Non-Inhibitor
M46	Inhibitor	Inhibitor	Non-Inhibitor	Non-Inhibitor	Non-Inhibitor
M47	Inhibitor	Non-Inhibitor	Non-Inhibitor	Non-Inhibitor	Non-Inhibitor
M48	Non-Inhibitor	Non-Inhibitor	Non-Inhibitor	Non-Inhibitor	Non-Inhibitor
M49	Inhibitor	Inhibitor	Inhibitor	Non-Inhibitor	Non-Inhibitor
M50	Non-Inhibitor	Non-Inhibitor	Non-Inhibitor	Non-Inhibitor	Non-Inhibitor
M53	Non-Inhibitor	Non-Inhibitor	Non-Inhibitor	Non-Inhibitor	Non-Inhibitor
M54	Inhibitor	Non-Inhibitor	Non-Inhibitor	Non-Inhibitor	Non-Inhibitor
M56	Non-Inhibitor	Non-Inhibitor	Non-Inhibitor	Non-Inhibitor	Non-Inhibitor
M59	Inhibitor	Inhibitor	Non-Inhibitor	Non-Inhibitor	Non-Inhibitor
M63	Inhibitor	Non-Inhibitor	Non-Inhibitor	Non-Inhibitor	Non-Inhibitor
M64	Inhibitor	Inhibitor	Non-Inhibitor	Non-Inhibitor	Non-Inhibitor
M67	Inhibitor	Inhibitor	Non-Inhibitor	Non-Inhibitor	Non-Inhibitor
M69	Inhibitor	Inhibitor	Inhibitor	Non-Inhibitor	Non-Inhibitor
M71	Inhibitor	Inhibitor	Inhibitor	Non-Inhibitor	Non-Inhibitor
M72	Inhibitor	Inhibitor	Inhibitor	Non-Inhibitor	Non-Inhibitor
M73	Inhibitor	Inhibitor	Inhibitor	Non-Inhibitor	Non-Inhibitor
M74	Inhibitor	Inhibitor	Inhibitor	Non-Inhibitor	Non-Inhibitor
M75	Inhibitor	Inhibitor	Inhibitor	Non-Inhibitor	Non-Inhibitor
M76	Inhibitor	Inhibitor	Inhibitor	Non-Inhibitor	Non-Inhibitor
M77	Inhibitor	Inhibitor	Inhibitor	Non-Inhibitor	Non-Inhibitor
M78	Inhibitor	Inhibitor	Inhibitor	Non-Inhibitor	Non-Inhibitor
M79	Inhibitor	Inhibitor	Inhibitor	Non-Inhibitor	Non-Inhibitor
M82	Inhibitor	Non-Inhibitor	Non-Inhibitor	Non-Inhibitor	Non-Inhibitor
M83	Inhibitor	Inhibitor	Non-Inhibitor	Non-Inhibitor	Non-Inhibitor
M86	Non-Inhibitor	Non-Inhibitor	Non-Inhibitor	Non-Inhibitor	Non-Inhibitor

Table S11: Predicted excretion profile of all pyrazole derviatives.

Excretion		
**Molecule**	**Total Clearance**	**Renal OCT2 substrate**
M3	0.774	No
M7	0.805	No
M8	0.803	No
M9	0.906	No
M10	0.774	No
M11	0.174	No
M12	0.171	No
M13	0.04	No
M14	0.824	No
M15	0.784	No
M16	0.796	No
M17	0.055	No
M18	0.629	No
M19	0.034	No
M20	0.824	No
M21	0.908	No
M22	0.713	Yes
M23	0.703	No
M24	0.812	Yes
M25	0.677	Yes
M26	0.239	Yes
M27	0.23	No
M28	0.098	Yes
M29	0.758	No
M31	0.721	No
M32	0.116	Yes
M33	0.543	Yes
M34	0.095	Yes
M36	0.809	No
M37	0.709	No
M38	0.846	No
M39	0.805	No
M40	0.773	No
M41	0.102	No
M42	0.095	No
M43	-0.189	No
M44	0.816	No
M45	0.742	No
M46	-0.019	No
M47	0.534	No
M48	0.809	No

M49	-0.041	No
M50	0.721	No
M53	0.102	No
M54	0.146	No
M56	0.033	No
M59	0.241	No
M63	0.124	No
M64	0.211	No
M67	0.222	No
M69	0.343	No
M71	0.307	No
M72	0.322	No
M73	0.315	No
M74	0.031	No
M75	0.373	No
M76	0.276	No
M77	0.202	No
M78	0.183	No
M79	0.18	No
M82	1.077	No
M83	0.935	No
M86	0.197	No

Table S12: *In silico* toxicity prediction of all pyrazole derivatives using pkCSM web tool.

Toxicity										
**Molecules**	**AMES toxicity**	**Max. tolerated dose (human)**	**hERG I inhibitor**	**hERG II inhibitor**	**Oral Rat Acute Toxicity (LD50)**	**Oral Rat Chronic Toxicity (LOAEL)**	**Hepatotoxicity**	**Skin Sensitisation**	**T.Pyriformis toxicity**	**Minnow toxicity**
M3	Yes	1.113	No	No	2.279	2.005	No	No	-0.468	3.394
M7	Yes	0.211	No	No	2.73	1.371	No	No	1.596	3.033
M8	Yes	0.26	No	No	2.509	1.383	No	No	0.96	1.853
M9	Yes	0.066	No	No	2.849	1.358	Yes	No	1.459	2.082
M10	Yes	-0.064	No	No	2.545	1.466	No	No	1.764	3.377
M11	Yes	0.183	No	No	2.978	1.283	Yes	No	1.498	1.87
M12	No	0.144	No	No	2.967	1.203	No	No	2.072	1.119
M13	No	0.156	No	No	2.468	1.495	No	No	1.196	1.715
M14	Yes	0.527	No	No	3.027	0.649	Yes	No	0.499	2.308
M15	No	0.152	No	No	2.63	1.178	No	No	0.652	2.114
M16	Yes	0.226	No	No	2.553	1.354	No	No	1.543	1.651
M17	Yes	0.123	No	No	2.961	1.291	Yes	No	2.039	2.719
M18	Yes	0.164	No	No	2.763	1.359	No	No	1.628	3.052
M19	Yes	0.11	No	No	2.959	1.281	Yes	No	2.091	2.573
M20	Yes	0.216	No	No	2.772	1.346	Yes	No	1.299	2.067
M21	Yes	0.694	No	No	2.449	1.373	No	No	1.308	0.796

M22	Yes	-0.101	No	No	2.192	1.389	No	No	2.006	1.895
M23	No	0.101	No	No	2.346	1.422	No	No	1.408	1.248
M24	Yes	0.022	No	No	2.508	1.385	Yes	No	1.974	1.167
M25	Yes	-0.039	No	No	3.172	1.364	No	No	1.299	1.947
M26	Yes	-0.118	No	No	2.307	1.309	No	No	2.138	1.581
M27	No	0.059	No	No	2.727	1.32	No	No	2.171	0.641
M28	Yes	-0.041	No	No	2.591	1.297	No	No	1.74	1.154
M29	No	-0.355	No	No	2.426	1.213	Yes	No	1.336	1.649
M31	Yes	0.096	No	No	2.472	1.381	No	No	2.057	1.173
M32	Yes	0.097	No	No	2.596	1.31	No	No	2.141	0.956
M33	No	0.091	No	No	2.472	1.379	No	No	1.994	1.289
M34	No	0.09	No	No	2.599	1.3	No	No	2.157	0.81
M36	No	0.225	No	Yes	2.276	2.261	No	No	0.894	0.054
M37	No	0.632	No	No	2.322	1.643	Yes	No	0.913	1.708
M38	No	0.545	No	No	2.402	1.474	Yes	No	0.824	1.429
M39	No	0.505	No	No	2.35	1.638	Yes	No	0.982	1.606
M40	Yes	0.741	No	No	2.411	1.946	Yes	No	1.108	1.28
M41	No	0.596	No	No	2.494	1.563	No	No	1.105	1.394
M42	No	0.559	No	No	2.64	1.482	Yes	No	1.203	1.08
M43	No	0.661	No	No	2.433	1.555	Yes	No	0.729	1.592
M44	No	0.318	No	No	2.601	0.782	Yes	No	0.426	2.642
M45	Yes	0.467	No	No	2.43	1.401	Yes	No	0.919	1.227
M46	No	0.596	No	No	2.494	1.563	Yes	No	1.105	1.394
M47	No	0.641	No	No	2.311	1.631	Yes	No	0.838	1.727
M48	No	0.26	No	No	2.309	1.276	Yes	No	0.504	1.33
M49	No	0.587	No	No	2.489	1.552	Yes	No	1.124	1.248
M50	No	0.585	No	No	2.341	1.626	No	No	0.998	1.591
M53	No	0.187	No	No	2.125	1.494	Yes	No	0.143	2.584
M54	Yes	0.228	No	No	2.501	1.321	Yes	No	0.758	1.249
M56	No	0.452	No	No	2.771	0.815	Yes	No	0.253	2.54
M59	Yes	0.13	No	No	2.605	1.075	Yes	No	0.857	0.666
M63	No	0.353	No	No	2.561	1.857	Yes	No	0.593	0.219
M64	Yes	0.188	No	No	2.527	1.076	Yes	No	0.882	0.672
M67	Yes	0.496	No	No	2.842	0.919	Yes	No	0.663	2.148
M69	No	0.629	No	No	2.523	2.61	Yes	No	0.62	-0.184
M71	No	-0.177	No	Yes	2.634	1.805	Yes	No	1.107	0.762
M72	No	-0.127	No	Yes	2.751	1.531	Yes	No	1.207	0.478
M73	No	-0.154	No	Yes	2.844	1.338	Yes	No	1.116	0.236
M74	No	-0.142	No	Yes	2.776	1.556	Yes	No	0.898	0.748
M75	Yes	0.298	No	No	2.948	1.018	Yes	No	0.546	1.012
M76	No	-0.119	No	Yes	2.615	1.649	Yes	No	1.253	0.768
M77	No	-0.116	No	Yes	2.743	1.523	Yes	No	1.208	0.55
M78	No	-0.1	No	No	2.621	1.798	Yes	No	1.103	0.883
M79	No	-0.124	No	Yes	2.736	1.495	Yes	No	1.204	0.404
M82	Yes	0.434	No	No	1.849	1.665	Yes	No	0.383	1.67
M83	No	0.286	No	Yes	2.672	1.298	Yes	No	0.305	1.457

M86	No	0.143	No	No	2.562	0.507	Yes	No	0.206	2.081

Synthesis section (characteristics of each molecules)

The target compounds were synthesized following the reported procedure **[1-12].**

(E)-N'-benzylidene-5-methyl-1H-pyrazole-3-carbohydrazide (M7) :

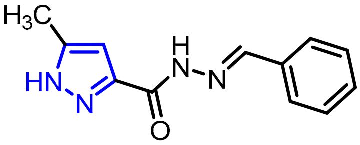

Yield 62% (solid), M.p. 243-245°C; FT-IR (ATR, ν(cm^{-1})) : 3229 (NH), 1656 (C=O); ^{1}H-NMR (300 MHz, DMSO-d_6, δ(ppm)): δ = 2.274 (s,3H, CH_3), 6.488 (s, 1H, Pz-H), 7.410 - 7.673 (m, 5H, ArH), 8.478 (s, 1H, CO-NH), 11.588 (s, 1H, N=CH) 13.076 (s, 1H, Pz-NH); ^{13}C-NMR (75 MHz, DMSO-d_6, δ(ppm)): δ = 10.77, 105.31, 127.41, 129.27, 130.31, 135.03, 140.53,146.30, 147.61, 158.87; MS: m/z = 229.3 $[M+H]^+$.

(E)-N'-(4-methoxybenzylidene)-5-methyl-1H-pyrazole-3-carbohydrazide (M8):

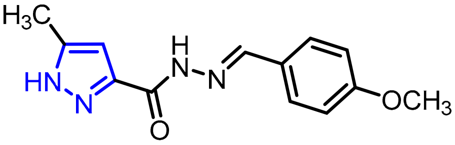

Yield (solid), 78 %, m.p. 260-262 °C; FT-IR (ATR, ν(cm^{-1})) : 3214 (NH), 1651 (C=O), 1605 (N=CH); ^{1}H-NMR (300 MHz, DMSO-d_6, δ(ppm)): 2.26 (s,3H, CH_3), 3.77 (s, 3H, OCH_3), 6.47 (s, 1H, H-pyrazole), 6.98 (d, J = 8.7 Hz, 2H, H-Ar), 7.60 (d, J = 8.7 Hz, 2H, H-Ar), 8.39 (s, 1H, -NH), 11.44 (s, 1H, N=CH) 13.05 (s, 1H, NH-pyrazole) ; ^{13}C NMR: (75MHz, DMSO-d_6, δ (ppm)): 10.80, 55.74, 105.22, 114.76, 127.55, 129.02, 140.45, 146.45, 147.53, 158.72, 161.13. MS: *m/z* = 259.5 $[M+H]^+$, 281.3 $[M+Na]^+$.

(E)-N'-(4-(dimethylamino)benzylidene)-5-methyl-1H-pyrazole-3-carbohydrazide (M9) :

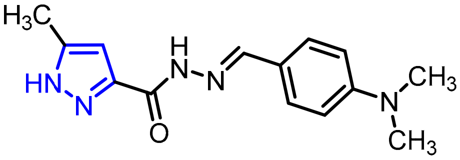

Yield (solid), 81 %, m.p. 259-261 °C; FT-IR (ATR, ν(cm^{-1})) : 3233 (NH), 1648 (C=O), 1602 (N=CH); ^{1}H-NMR (300 MHz, DMSO-*d6*, δ(ppm)): δ = 2.26 (s, 3H, CH_3), 2.94 (s, 6H, $N(CH_3)_2$), 6.45 (s, 1H, H-pyrazole), 6.72 (d, J = 8.7 Hz, 2H, H-Ar), 7.46 (d, J = 8.7 Hz, 2H, H-Ar), 8.30 (s, 1H, -NH), 11.24 (s, 1H, N=CH) 13.01 (s, 1H, NH-pyrazole) ; ^{13}C NMR:

(75MHz, DMSO-d6, δ (ppm)): 10.78, 40.28, 105.11, 112.26, 122.33, 128.76, 140.36, 146.60, 148.42, 151.84, 158.45. EST-MS: m/z = 272.3 $[M+H]^+$.

(E)-5-methyl-N'-(4-nitrobenzylidene)-1H-pyrazole-3-carbohydrazide (M10) :

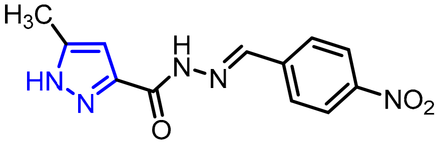

Yield 84% (solid), m.p. 283-285°C; FT-IR (ATR, $\nu(cm^{-1})$) : 3320 (NH), 1681 (C=O), 1512 (N=CH); ^{1}H-NMR (300 MHz, DMSO-d6, δ(ppm)): δ = 2.28 (s,3H, CH_3), 6.44 (s, 1H, H-pyrazole), 7.90 (d, J = 8.7 Hz, 2H, H-Ar), 8.27 (d, , J = 8.7 Hz, 2H, H-Ar), 8.58 (s, 1H, -CONH),11. 92 (s, 1H, N=CH), 13.13 (s, 1H, NH-pyrazole); ^{13}C NMR: (75MHz, DMSO-d6, δ (ppm)): 10.77, 105.52, 124.25, 128.33, 140.71, 141.40, 145.11, 146.01, 148.14, 159.10; MS: m/z = 274.1 $[M+H]^+$.

(E)-N'-(2-chlorobenzylidene)-5-methyl-1H-pyrazole-3-carbohydrazide (M11) :

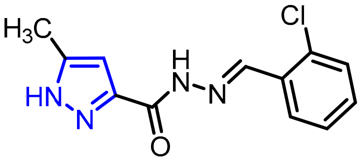

Yield 78% (solid), m.p. 258-260°C; FT-IR (ATR, $\nu(cm^{-1})$) : 3182 (NH), 1665 (C=O), 1552 (N=CH); ^{1}HNMR (300 MHz, DMSO-d6, δ(ppm)): δ = 2.27 (s, 3H, CH_3), 6.49 (s, 1H, H-pyrazole), 7.39 - 7.99 (m, 4H, H-pyrazole), 8.90 (s, 1H, -N=CH), 11. 92 (s, 1H, -CONH), 13.09 (s, 1H, NH-pyrazole) ; ^{13}C NMR: (75MHz, DMSO-d6, δ (ppm)): 10.77, 105.40, 127.31, 128.01, 130.34, 131.70, 132.41, 133.57, 140.56, 143.65, 146.16, 159.05; ESI-MS: m/z = 263.1 $[M+H]^+$.

(E)-N'-(2,4-dichlorobenzylidene)-5-methyl-1H-pyrazole-3-carbohydrazide (M12)

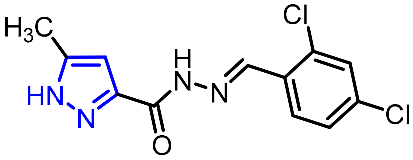

Yield 95% (solid), m.p. 258-260°C; FT-IR (ATR, $\nu(cm^{-1})$) : 3326 (NH), 11684 (C=O), 1584 (N=CH); ^{1}H-NMR (300 MHz, DMSO-*d6*, δ(ppm)): δ = 2.277 (s, 3H, CH_3), 6.498 (s, 1H, Pz-H), 7.475 - 7.509 (dd, 1H, ArH), 7.678, 7.685 (d, 1H, ArH), 7.963, 7.991 (d, 1H, ArH), 8.866 (s, 1H, -N=CH), 11. 986 (s, 1H, CONH), 13.106 (s, 1H, Pz-NH) ; ESI-MS: m/z = 297.1 $[M+H]^+$.

(E)-N'-(2-bromo-5-hydroxybenzylidene)-5-methyl-1H-pyrazole-3-carbohydrazide (M13)

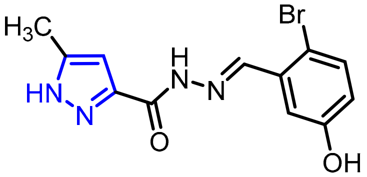

Yield 63% (solid), m.p. 254-256°C; FT-IR (ATR, ν(cm^{-1})) : 3314 (NH), 1692 (C=O), 1533 (N=CH); ^{1}H NMR (300 MHz, d6-DMSO, δ(ppm)): δ = 2.278 (s,3H, –CH3), 6.503 (s, 1H, OH), 6.506 (s, 1H, Pz-H), 6.856, 6.886 (d, 1H, ArH), 7.374 - 7.412 (dd, 1H, ArH), 7.657, 7.665 (d, 1H, ArH), 8.614 (s, 1H, -N=CH), 11. 379 (s, 1H, -CONH), 13.128 (s, 1H, Pz-NH); ^{13}C NMR (75 MHz, DMSO-d6, δ(ppm)): δ = 10.77, 105.46, 110.77, 119.14, 121.77, 131.21, 133.77, 140.64, 145.78, 146.13, 156.88, 158.87; ESI-MS: m/z = 323.2 $[M+H]^{+}$.

(E)-N'-(furan-2-ylmethylene)-5-methyl-1H-pyrazole-3-carbohydrazide (M14) :

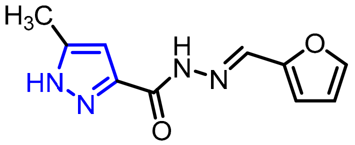

Yield 73% (solid), M.p. 272-274°C; FT-IR (ATR, ν(cm^{-1})) : 3224 (NH), 1652 (C=O), 1545 (N=CH); 1H-NMR (300 MHz, DMSO-d6, δ(ppm)): δ = 2.269 (s,3H, –CH3), 6.467 (s, 1H, Pz-H), 6.591 – 6.608 (m, 1H, FurH), 6.826 – 6.837 (d, 1H, FurH 7.800 – 7.803 (d, 1H, FurH) 8.367 (s, 1H, -N=CH), 11.592 (s, 1H, -CO-NH), 13.059 (s, 1H, Pz-NH); ^{13}C NMR (75 MHz, DMSO-d6, δ(ppm)): δ = 10.77, 105.31, 121.62, 113.22, 137.41, 140.54, 145.35, 146.27, 150.20, 158.80 ; ESI-MS: m/z = 219.3 $[M+H]^{+}$.

(E)-N'-(4-hydroxy-3-methoxybenzylidene)-5-methyl-1H-pyrazole-3-carbohydrazide (M15) :

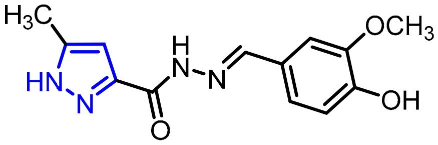

Yield 65 % (solid), m.p. 226-228 °C; FT-IR (ATR, ν(cm^{-1})) : 3483 (OH), 3258 (NH), 1648 (C=O), 1590 (N=CH); ^{1}H-NMR (300 MHz, DMSO-d_6, δ(ppm)): δ = 2.26 (s,3H, CH_3), 3.80 (s,3H, OCH_3), 6.46 (s, 1H, H-pyrazole), 6.80 (d, J = 8.1 Hz, 1H, H-Ar), 7.00 (d, J = 8.1 Hz, 1H, H-Ar), 7.26 sd, 1H, H-Ar), 8.33 (s, 1H, CONH), 9.51 (s, 1H, OH), 11.38 (s, 1H, N=CH) 13.03 (s, 1H, NH-pyrazole) ; ^{13}C NMR: (75MHz, DMSO-d_6, δ (ppm)): 10.77 (CH_3), 56.00 (OCH_3), 105.20 (CH, C4-pyrazole), 109.20 (CH, C6-Ar), 115.86 (CH, C3-Ar), 122.43 (CH,

C2-Ar), 126.42 (C, C1-Ar), 148.20 (C, C3-pyrazole), 148.47 (CH, N=CH), 149.23 (C, C5-pyrazole), 149.85 (C, OCH_3), 158.91 (C, OH), 158.91 (C, C=O); ESI-MS: m/z = 275.2 $[M+H]^+$.

(E)-5-methyl-N'-(4-methylbenzylidene)-1H-pyrazole-3-carbohydrazide (M16) :

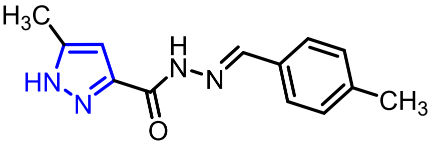

Yield 59 % (solid), m.p. 292-294 °C; FT-IR (ATR, ν(cm^{-1})) : 3224 (NH), 1654 (C=O), 1606 (N=CH); ^{1}H-NMR (300 MHz, DMSO-d_6, δ(ppm)): δ = 2.26 (s,3H, CH_3), 2.31 (s, 3H, CH_3), 6.50 (s, 1H, H-pyrazole), 7.23 (d, J = 8.1 Hz, 2H, H-Ar), 7.55 (d, J = 8.1 Hz, 2H, H-Ar), 8.42 (s, 1H, -NH), 11.98 (s, 1H, N=CH) 13.10 (s, 1H, NH-pyrazole) ; ^{13}C NMR: (75MHz, DMSO-d_6, δ (ppm)): 11.06 (CH_3), 21.48 (CH_3), 105.27 (CH, C4-pyrazole), 127.42 (CH, C2-Ar), 129.88 (CH, C3-Ar), 132.28 (C, C1-Ar), 140.13 (C, C1-Ar), 141.73 (C, C3-pyrazole), 146.95 (CH, N=CH), 147.13 (C, C5-pyrazole), 158.80 (C, C=O); ESI-MS: m/z = 243.1 $[M+H]^+$.

(E)-N'-(4-chlorobenzylidene)-5-methyl-1H-pyrazole-3-carbohydrazide (M17) :

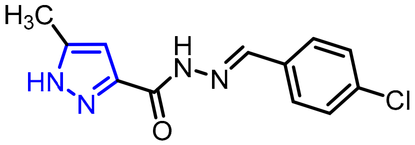

Yield 64 % (solid), m.p. 301-303 °C; FT-IR (ATR, ν(cm^{-1})) : 3237 (NH), 1654 (C=O), 1608 (N=CH); ^{1}H-NMR (300 MHz, DMSO-d_6, δ(ppm)): δ = 2.27 (s,3H, CH_3), 6.49 (s, 1H, H-pyrazole), 7.47 (d, J = 8.7 Hz, 2H, H-Ar), 7.68 (d, J = 8.7 Hz, 2H, H-Ar), 8.45 (s, 1H, CONH), 11.67 (s, 1H, N=CH) 13.09 (s, 1H, NH-pyrazole) ; ^{13}C NMR: (75MHz, DMSO-d_6, δ (ppm)): 10.77 (CH_3), 105.35 (CH, C4-pyrazole), 129.04 (CH, C3-Ar), 129.37 (CH, C2-Ar), 133.98 (C, C1-Ar), 134.70 (C, C4-Ar), 140.57 (C, C3-pyrazole), 146.27 (CH, N=CH), 147.23 (C, C5-pyrazole), 158.91 (C, C=O); ESI-MS: m/z = 263.2 $[M+H]^+$.

(E)-N'-(4-fluorobenzylidene)-5-methyl-1H-pyrazole-3-carbohydrazide (**M18**) :

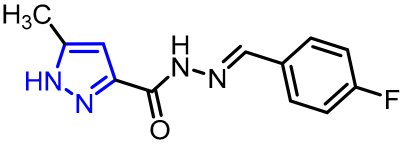

Yield 75 % (solid), m.p. 310-312 °C; FT-IR (ATR, ν(cm^{-1})) : 3189 (NH), 1666 (C=O), 1604 (N=CH); ^{1}H-NMR (300 MHz, DMSO-d_6, δ(ppm)): δ = 2.27 (s,3H, CH_3), 6.48 (s, 1H, H-

pyrazole), 7.47 (d, J = 8.7 Hz, 2H, H-Ar), 7.68 (d, J = 8.7 Hz, 2H, H-Ar), 8.46 (s, 1H, CONH), 11.59 (s, 1H, N=CH) 13.07 (s, 1H, NH-pyrazole) ; ^{13}C NMR: (75MHz, DMSO-d$_6$, δ (ppm)): 10.77 (CH$_3$), 105.31 (CH, C4-pyrazole), 116.19 (CH, C3-Ar), 129.50 (CH, C2-Ar), 131.63 (C, C1-Ar), 140.55 (C, C3-pyrazole), 146.28 (CH, N=CH), 158.89 (C, C5-pyrazole), 161.80 (C, C=O), 165.08 (C, C4-Ar); ESI-MS: m/z = 247.1 [M+H]$^+$.

(E)-N'-(4-bromobenzylidene)-5-methyl-1H-pyrazole-3-carbohydrazide (**M19**) :

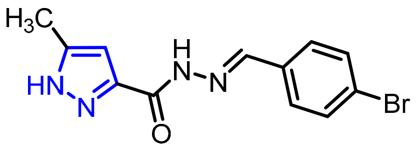

Yield 80 % (solid), m.p. 300-302 °C; FT-IR (ATR, ν(cm^{-1})) : 3226 (NH), 1655 (C=O), 1592 (N=CH); ^{1}H-NMR (300 MHz, DMSO-d$_6$, δ(ppm)): δ = 2.26 (s,3H, CH$_3$), 6.49 (s, 1H, H-pyrazole), 7.42 (d, J = 8.7 Hz, 2H, H-Ar), 7.61 (d, J = 8.7 Hz, 2H, H-Ar), 8.45 (s, 1H, CONH), 11.67 (s, 1H, N=CH) 13.09 (s, 1H, NH-pyrazole) ; ^{13}C NMR: (75MHz, DMSO-d$_6$, δ (ppm)): 10.76 (CH$_3$), 105.36 (CH, C4-pyrazole), 123.48 (CH, C3-Ar), 129.28 (CH, C2-Ar), 132.28 (C, C1-Ar), 134.32 (C, C4-Ar), 140.57 (C, C3-pyrazole), 146.27 (CH, N=CH), 146.36 (C, C5-pyrazole), 158.91 (C, C=O); ESI-MS: m/z = 308.1 [M+H]$^+$.

(E)-5-methyl-N'-(1-phenylethylidene)-1H-pyrazole-3-carbohydrazide (**M20**) :

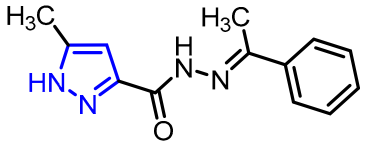

Yield 80 % (solid), m.p. 305-307 °C; FT-IR (ATR, ν(cm^{-1})) : 3242 (NH), 1655 (C=O), 1591 (N=CH); ^{1}H-NMR (300 MHz, DMSO-d$_6$, δ(ppm)): δ = 2.27 (s,3H, CH$_3$), 2.31 (s,3H, CH$_3$), 6.51 (s, 1H, H-pyrazole), 7.40-7.82 (m, 5H, H-Ar), 10.21 (s, 1H, CONH), 13.09 (s, 1H, NH-pyrazole) ; ^{13}C NMR: (75MHz, DMSO-d$_6$, δ (ppm)): 10.80, 13.90, 105.17, 126.81, 128.84, 129.82, 138.47, 140.82, 146.32, 153.13, 158.56; ESI-MS: m/z = 243.1 [M+H]$^+$.

N'-(diphenylmethylene)-5-methyl-1H-pyrazole-3-carbohydrazide (**M21**) :

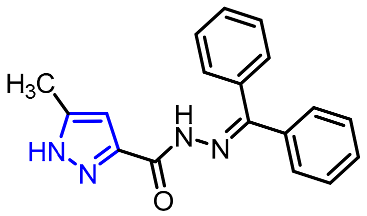

Yield 87 % (solid), m.p. 321-323 °C; IR (ATR, ν(cm^{-1})) : 3241 (NH), 1655 (C=O), 1592 (N=CH); ^{1}H-NMR (300 MHz, DMSO-d_6, δ(ppm)): δ = 2.19 (s,3H, CH_3), 6.46 (s, 1H, H-pyrazole), 7.36-7.65 (m, 10H, H-Ar), 9.80 (s, 1H, CONH), 12.99 (s, 1H, NH-pyrazole) ; ^{13}C NMR: (75MHz, DMSO-d_6, δ (ppm)): 10.77, 13.90, 105.10, 127.66, 128.66, 130.31, 138.47, 137.28, 141.08, 145.70, 153.35, 158.62; ESI-MS: m/z = 304.9 $[M+H]^+$.

(E)-N'-benzylidene-5-phenyl-1H-pyrazole-3-carbohydrazide (**M22**) :

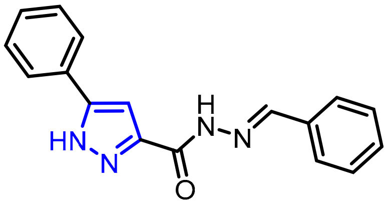

Yield 70% (solid), m.p. 202-204°C; FT-IR [(ATR, ν(cm^{-1})] : 3250 (NH), 1644 (C=O), 1557 (N=CH); ^{1}H-NMR [(300 MHz, DMSO-d6, δ(ppm)]: δ = 7.215 (s, 1H, Pz-H), 7.371 – 7.834 (m, 10H, Ar-H), 8.523 (s, 1H, N=CH), 11.725 (s, 1H, -CONH), 13.812 (s, 1H, Pz-NH) ; ^{13}C-NMR [(75 MHz, DMSO-d6, δ(ppm)]: 105.35 (CH, C4-pyrazole), 125.88 (CH, C2-Ar), 127.52 (CH, C3-Ar), 128.65 (CH, C4-Ar), 129.08 (CH, C3-Ar), 129.31 (CH, C2-Ar), 129.53 (CH, C4-Ar), 130.43 (C, C1-Ar), 134.95 (C, C4-Ar), 144.20 (CH=N), 147.19 (C, C3-pyrazole), 148.06 (C, C5-pyrazole), 158.65 (C=O) ; ESI-MS: m/z = 291.1 $[M+H]^+$.

(E)-N'-(4-methoxybenzylidene)-5-phenyl-1H-pyrazole-3-carbohydrazide (**M23**)

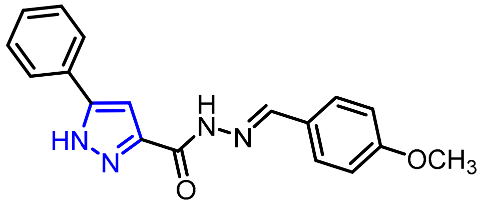

Yield 82% (solid); m.p. 276–278 °C; FT-IR (ATR, ν(cm^{-1})): 3214 (NH), 1656 (C=O), 1603 (N = CH); ^{1}H-NMR (300 MHz, DMSO-d6: δ = 3.78 (s, 3H, OCH_3), 7.00 (s, 1H, CH-pz), 7.21 (d, J = 8.7Hz, 2H, H-Ar), 7.36–7.48 (m, 5H, H-Ar), 7.64 (d, J = 8.7Hz, 2H, H-Ar), 8.44 (s, 1H, NHCO), 11.59 (s, 1H, N = CH), 13.80 (s, 1H, NH-pz); ^{13}C-NMR (75 MHz, DMSO-d6: δ = 55.75, 103.87, 114.79, 125.81, 127.44, 128.50, 129.16, 129.50, 130.11, 144.15, 147.27, 148.02, 158.49, 161.26; ESI-MS: m/z = 321.0 $[M+H]^+$.

(E)-N'-(4-(dimethylamino)benzylidene)-5-phenyl-1H-pyrazole-3-carbohydrazide (**M24**) :

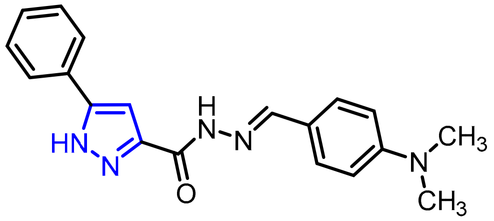

Yield 78% (solid); m.p. 262–264 °C; FT-IR (ATR, ν(cm^{-1})) : 3219 (NH), 1648 (C = O); 1H-NMR (300 MHz, DMSO-d6, δ(ppm)): δ = 2.95 (s, 6H, 2 × CH3), 6.72 (s, 1H, CHpz), 7.16–6.72 (m, 9H, H-Ar), 8.35 (s, 1H, NHCO), 11.35 (s, 1H, N = CH), 13.72 (s, 1H, NH-pz) ; 13C-NMR (75 MHz, DMSO-d6, δ(ppm)): δ = 40.95, 103.73, 112.26, 122.15, 125.80, 128.91, 129.31, 129.73, 133.95, 144.20, 147.19, 148.06, 151.96, 158.12.; ESI-MS : m/z = 334.5 $[M+H]^+$.

(E)-5-phenyl-N'-(4-nitrobenzylidene)-1H-pyrazole-3-carbohydrazide (**M25**):

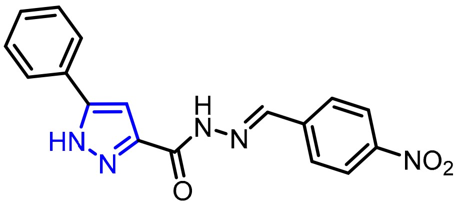

Yield 72% (solid), m.p. 288-290°C; FT-IR [(ATR, ν(cm^{-1})] : 3225 (NH), 1669 (C=O), 1510 (N=CH); ^{1}H-NMR [(300 MHz, d_6-DMSO, δ(ppm)]: δ = 7.237 (s, 1H, Pz-H), 7.386 – 8.306 (m, 9H, ArH), 8.636 (s, 1H, N=CH), 12.047 (s, 1H, -CON-H), 13.839 (s, 1H, Pz-NH) ; ^{13}C-NMR [(75 MHz, DMSO-d6, δ(ppm)]: 103.92 (C4-pyrazole), 124.54 (3×CH), 125.81 (3×CH), 128.42 (3×CH), 128.96 (C), 129.50 (2×C), 141.21 (CH=N), 145.64 (C3-pyrazole), 148.24 (C5-pyrazole), 158.21 (C=O); ESI-MS: m/z = 336.2 $[M+H]^+$.

(E)-N'-(2-chlorobenzylidene)-5-phenyl-1H-pyrazole-3-carbohydrazide (**M26**) :

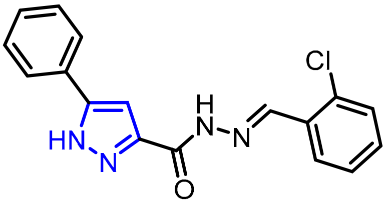

Yield 89% (solid), m.p. 230-232°C; FT-IR [(ATR, ν(cm^{-1})] : 3145 (NH), 1643 (C=O), 1556 (N=CH); ^{1}HNMR (300 MHz, DMSO-d6, δ(ppm)): δ = 7.233 (s, 1H, Pz-H), 7.368 – 8.034 (m, 9H, ArH), 8.950 (s, 1H, N=CH), 12.074 (s, 1H, -CON-H), 13.834 (s, 1H, Pz-NH); ^{13}C-NMR [(75 MHz, DMSO-d6, δ(ppm)]: 103.822 (CH, C4-pyrazole), 125.82 (CH, C2-Ar), 127.53 (CH, C3-Ar), 127.90 (CH, C4-Ar), 128.99 (CH, C3-Ar), 129.49 (CH, C2-Ar), 129.91 (CH,

C4-Ar), 132.18 (C, C1-Ar), 140.28 (C, C4-Ar), 148.20 (CH=N), 156.88 (C, C3-pyrazole), 158.62 (C, C5-pyrazole), 164.13 (C=O) ; ESI-MS: m/z = 325.3 $[M+H]^+$.

(E)-N'-(2,4-dichlorobenzylidene)-5-phenyl-1H-pyrazole-3-carbohydrazide (**M27**) :

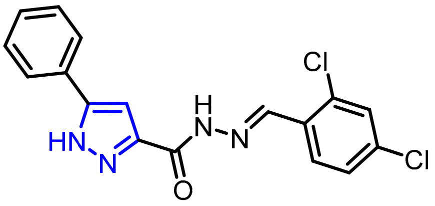

Yield 62 % (solid), m.p. 234-236 °C; FT-IR (ATR, ν(cm^{-1})) : 3182 (NH), 1657 (C=O), 1586 (N=CH); ^{1}H-NMR (300 MHz, DMSO-d_6, δ(ppm)) : 7.24 (s, 1H, H-pyrazole), 7.52-7.34 (m, 4H, H-Ar), 7.69 (d, J = 1.8 Hz, 2H, H-Ar), 7.82 (d, J = 7.5 Hz, 2H, H-Ar), 8.01 (d, J = 8.4 Hz, 2H, H-Ar), 8.89 (s, 1H, -CONH), 12.12 (s, 1H, N=CH) 13.83 (s, 1H, NH-pyrazole); MS: m/z = 359.0 $[M+H]^+$, 381.0 $[M+Na]^+$.

(E)-N'-(2-bromo-5-hydroxybenzylidene)-5-phenyl-1H-pyrazole-3-carbohydrazide (**M28**) :

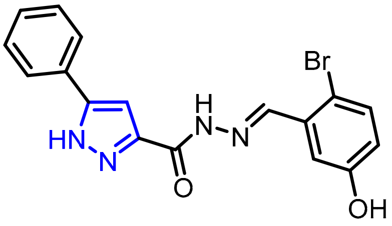

Yield 91% (solid), m.p. 287-289°C; FT-IR (ATR, ν(cm^{-1})) : 3205 (NH), 1664 (C=O), 1615 (N=CH); ^{1}H NMR (300 MHz, DMSO-d_6, δ(ppm)): δ = 6.872 (s, 1H, OH), 7.223 (s, 1H, PzH),7.381 – 7.840 (m, 8H, ArH), 8.674 (s, 1H, N=CH), 12.144 (s, 1H, -CON-H), 13.838 (s, 1H, Pz-NH) ; ESI-MS: m/z = 386.2 $[M+H]^+$.

(E)-N'-(furan-2-ylmethylene)-5-phenyl-1H-pyrazole-3-carbohydrazide (**M29**) :

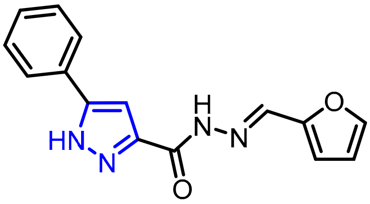

Yield 85% (solid), m.p. 207-209°C; FT-IR (ATR, ν(cm^{-1})) : 3276 (NH), 1681 (C=O), 1611 (N=CH); ^{1}H-NMR (300 MHz, DMSO-d_6, δ(ppm)): δ = 6.609 - 6.654 (m, 1H, Fur-H), 6.871 (s, 1H, Pz-H) 7.186 – 7.825 (m, 7H, Ar-H, Fur-H), 8.417 (s, 1H, N=CH), 11.717 (s, 1H, CON-H), 13.767 (s, 1H, Pz-NH) ; ESI-MS: m/z = 281.3 $[M+H]^+$.

(E)-N'-(4-hydroxy-3-methoxybenzylidene)-5-phenyl-1H-pyrazole-3-carbohydrazide (**M30**) :

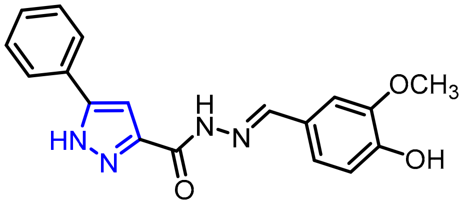

Yield: 85% (solid); m.p: 229–231 °C; FT-IR (ATR, ν(cm^{-1})): 3276 (NH), 1681 (C=O), 1568 (C=N); ^{1}H NMR: (300 MHz, DMSO-d_6, δ(ppm)): 3.81 (3H, s, -OCH_3), 6.83 (d, J = 8.1 Hz, 1*H*, H-Ar), 7.18 (1*H*, s, CH-pyrazole), 7.26–7.49 (5*H*, m, Ar-H), 7.54 (s, 1*H*, H-Ar), 7.82 (d, J = 8.1 Hz, 1*H*, H-Ar), 8.39 (1*H*, s, N=CH), 11.51 (s, 1*H*, OH), 11.72 (s, 1*H*, NHCO), 13.72 (1*H*, s, NH-pyrazole); ^{13}C NMR: (75MHz, DMSO-d6, δ (ppm)): 56.04 (OCH_3), 103.85 (CH, C4-pyrazole), 115.91 (CH, C-Ar), 122.53 (CH, C-Ar), 125.53 (CH, C-Ar), 126.37 (CH, C-Ar), 128.28 (CH, C-Ar), 129.07 (CHC-Ar), 129.33 (C, C-Ar), 129.54 (C-Ar), 144.12 (CH, N=CH), 145.85 (C, C3-pyrazole), 147.36 (C, C5-pyrazole), 148.63 (C, C-OH), 156.48 (C, C-OCH_3), 158.40 (C, C=O). ESI-MS: *m*/*z* = 337.0 $[M+H]^+$, 359.0 $[M+Na]^+$.

(E)-5-phenyl-N'-(4-methylbenzylidene)-1H-pyrazole-3-carbohydrazide (**M31**) :

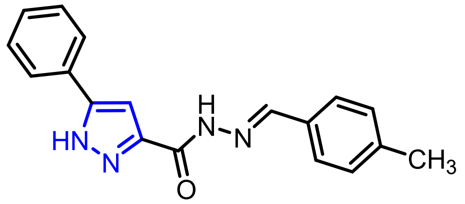

Yield 90% (solid); m.p: 297–299 °C; FT-IR (ATR, ν(cm^{-1})): 3205 (NH), 1680 (C=O), 1561 (C=N); ^{1}H NMR: (300 MHz, DMSO-d_6, δ(ppm)): 2.32 (3*H*, s, CH_3), 7.25 (1*H*, s, CH-pyrazole), 7.33–7.61 (5*H*, m, Ar-H), 7.59 (d, J = 7.8 Hz, 2*H*, H-Ar), 7.81 (d, J = 7.8 Hz, 1*H*, H-Ar), 8.45 (1*H*, s, N=CH), 11.65 (s, 1*H*, NHCO), 13.78 (1*H*, s, NH-pyrazole); ^{13}C NMR: (75MHz, DMSO-d_6, δ (ppm)): 21.50 (OCH_3), 103.85 (CH, C4-pyrazole), 125.80 (CH, C-Ar), 127.53 (CH, C-Ar), 127.90 (CH, C-Ar), 128.94 (CH, C-Ar), 129.49 (CH, C-Ar), 129.92 (C, C-Ar), 132.18 (C, C-Ar), 136.80 (C, C-CH_3), 140.28 (C, C3-pyrazole), 146.18 (CH, N=CH), 148.28 (C, C5-pyrazole), 158.50 (C, C=O). ESI-MS: *m*/*z* = 304.9 $[M+H]^+$, 326.9 $[M+Na]^+$.

(E)-N'-(4-chlorobenzylidene)-5-phenyl-1H-pyrazole-3-carbohydrazide (**M32**) :

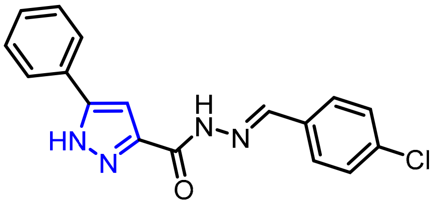

Yield 89% (solid); m.p: 301–303 °C; FT-IR (ATR, ν(cm^{-1})): 3207 (NH), 1680 (C=O), 1605 (C=N); ^{1}H NMR: (300 MHz, DMSO-d$_6$, δ(ppm)): 7.11 (1*H*, s, CH-pyrazole), 7.33–7.60 (5*H*, m, Ar-H), 7.59 (d, J = 7.2 Hz, 2*H*, H-Ar), 7.81 (d, J = 7.2 Hz, 1*H*, H-Ar), 8.46 (1*H*, s, N=CH), 11.65 (s, 1*H*, NHCO), 13.79 (1*H*, s, NH-pyrazole); ^{13}C NMR: (75MHz, DMSO-d6, δ (ppm)): 103.82 (CH, C4-pyrazole), 125.82 (CH, C-Ar), 127.53 (CH, C-Ar), 127.90 (CH, C-Ar), 128.99 (CH, C-Ar), 129.49 (CH, C-Ar), 129.91 (C, C-Ar), 132.18 (C, C-Ar), 140.28 (C, C-CH$_3$), 148.20 (C, C3-pyrazole), 156.88 (CH, N=CH), 158.62 (C, C5-pyrazole), 164.13 (C, C=O). ESI-MS: *m*/*z* = 325.1 [M+H]$^+$, 347.3 [M+Na]$^+$.

(E)-N'-(4-fluorobenzylidene)-5-phenyl-1H-pyrazole-3-carbohydrazide (**M33**) :

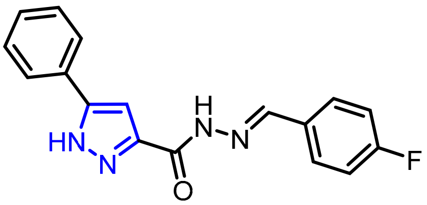

Yield 98% (solid); m.p: 294–296 °C; FT-IR (ATR, ν(cm^{-1})) : 3320 (NH), 1672 (C=O), 1604 (C=N); ^{1}H NMR: (300MHz, DMSO-d$_6$, δ(ppm)): 7.21 (1*H*, s, CH-pyrazole), 7.25–7.38 (5*H*, m, Ar-H), 7.42 (d, J = 7.8 Hz, 2*H*, H-Ar), 7.81 (d, J = 7.8 Hz, 1*H*, H-Ar), 8.50 (1*H*, s, N=CH), 11.72 (s, 1*H*, NHCO), 13.79 (1*H*, s, NH-pyrazole); ^{13}C NMR: (75 MHz, DMSO-d$_6$, δ (ppm)): 103.86 (CH, C4-pyrazole), 125.82 (CH, CH-Ar), 128.98 (CH, C-Ar), 129.49 (CH, CH-Ar), 129.64 (C, C-Ar), 129.74 (CH, CH-Ar), 130.10 (C, CH-Ar), 130.22 (C, C-Ar), 146.97 (C, C3-pyrazole), 150.50 (CH, N=CH), 156.68 (C, C5-pyrazole), 161.90 (C, C=O), 165.18 (C, C-F). ESI-MS: *m*/*z* = 309.3 [M+H]$^+$.

(E)-N'-(4-bromobenzylidene)-5-phenyl-1H-pyrazole-3-carbohydrazide (**M34**) :

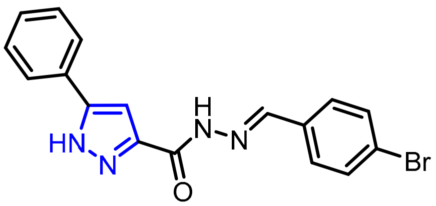

Yield 85% (solid); m.p: 291–293 °C; FT-IR (ATR, ν(cm^{-1})) : 3319 (NH), 1670 (C=O), 1589 (C=N); ^{1}H NMR: (300MHz, DMSO-d$_6$, δ(ppm)): 7.22 (1*H*, s, CH-pyrazole), 7.37 (d, J = 7.2

Hz, 2*H*, H-Ar), 7.43–7.64 (5*H*, m, Ar-H), 7.82 (d, J = 7.2 Hz, 1*H*, H-Ar), 8.48 (1*H*, s, N=CH), 11.80 (s, 1*H*, NHCO), 13.81 (1*H*, s, NH-pyrazole); ^{13}C NMR: (75 MHz, DMSO-d$_6$, δ (ppm)): 103.96, 123.63, 125.84, 129.03, 129.39, 129.75, 132.32, 133.60, 134.19, 144.71, 146.86, 148.63, 158.58. ESI-MS: *m*/*z* = 368.1 $[M+H]^+$.

(E)-5-phenyl-N'-(1-phenylethylidene)-1H-pyrazole-3-carbohydrazide (**M35**) :

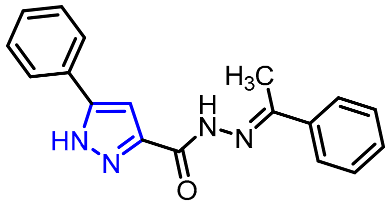

Yield 85% (solid); m.p: 251–253 °C; FT-IR (ATR, ν(cm^{-1})) : 3320 (NH), 1667 (C=O), 1589 (C=N); ^{1}H NMR: (300 MHz, DMSO-d6, δ(ppm)): 2.36 (3*H*, s, CH_3), 7.24 (1*H*, s, CH-pyrazole), 7.42–7.84 (10*H*, m, Ar-H), 10.37 (s, 1*H*, NHCO), 13.82 (1*H*, s, NH-pyrazole); ^{13}C NMR: (75 MHz, DMSO-d$_6$, δ (ppm)): 21.51 (OCH_3), 103.60 (CH, C4-pyrazole), 125.73 (CH, C-Ar), 127.72 (CH, C-Ar), 128.70 (CH, C-Ar), 129.01 (CH, C-Ar), 129.18 (CH, C-Ar), 129.56 (CH, C-Ar), 130.34 (C, C-Ar), 132.07 (C, C-Ar), 137.21 (C, C3-pyrazole), 144.82 (CH, N=CH), 146.45 (C, C5-pyrazole), 157.23 (C, C=O). ESI-MS: *m*/*z* = 305.4 $[M+H]^+$.

N'-(diphenylmethylene)-5-phenyl-1H-pyrazole-3-carbohydrazide (**M36**) :

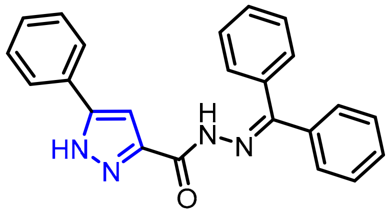

Yield 82% (solid); m.p: 200–202 °C; FT-IR (ATR, ν(cm^{-1})): 3360 (NH), 1664 (C=O), 1537 (C=N); ^{1}H NMR: (300 MHz, DMSO-d$_6$, δ(ppm)): 7.20 (1*H*, s, CH-pyrazole), 7.32–7.76 (15*H*, m, Ar-H), 9.89 (s, 1*H*, NHCO), 13.84 (1*H*, s, NH-pyrazole); ^{13}C NMR: (75 MHz, DMSO-d$_6$, δ (ppm)): 105.10 (CH, C4-pyrazole), 127.66 (CH, C-Ar), 128.66 (CH, C-Ar), 128.96 (CH, C-Ar), 130.21 (CH, C-Ar), 130.31 (CH, C-Ar), 130.46 (CH, C-Ar), 132.02 (C, C-Ar), 137.28 (C, C-Ar), 141.08 (C, C3-pyrazole), 145.70 (CH, N=CH), 153.35 (C, C5-pyrazole), 157.62 (C, C=O). ESI-MS: *m*/*z* = 366.9 $[M+H]^+$, 389.0 $[M+H]^+$.

(E)-N'-benzylidene-2-(3,5-dimethyl-1H-pyrazol-1-yl)acetohydrazide (**M37**) :

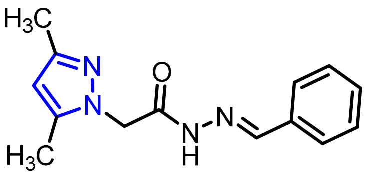

Yield 75% (solid) ; m.p : 170-172 °C ; FT-IR (ATR, ν(cm^{-1})) : 3369 (NH), 1678 (C=O), 1619 (C=N); ^{1}H NMR: (300MHz, DMSO-d_6, δ(ppm)) : 2.05 (s, 3H,CH_3), 2.13 (s, 3H, CH_3),5.19 (s, 2H, N-<u>CH2</u>-), 5.80 (1H, s, CH-pyrazole), 7.41-7.70 (m, 5H, H-Ar), 7.26-7.49 (5H, m, Ar-H), 8.00 (1H, s, N=CH), 11.59 (s, 1H, NHCO); ^{13}C NMR: (75MHz, DMSO-d_6, δ (ppm)): 11.13 (CH_3), 13.73 (CH_3), 50.82 (-CH_2), 105.21 (CH, C4-pyrazole), 127.60 (CH, C-Ar), 129.28 (CH, C-Ar), 130.45 (CH, C-Ar), 134.43 (C-Ar), 140.56 (C, C3-pyrazole), 144.55 (CH, N=CH), 147.94 (C, C5-pyrazole), 169.04 (C, C=O). ESI-MS: m/z = 257.2 $[M+H]^+$.

(E)-2-(3,5-dimethyl-1H-pyrazol-1-yl)-N'-(4-methoxybenzylidene)acetohydrazide (**M38**) :

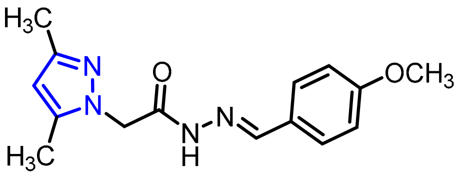

Yield 60% (solid), m.p : 185-187 °C ; FT-IR (ATR, ν(cm^{-1})) : 3114 (NH), 1676 (C=O), 1606 (C=N); ^{1}H NMR: (300MHz, DMSO-d_6, δ(ppm)) : 2.05 (s, 3H, CH_3), 2.12 (s, 3H, CH_3),3.78 (, s, 3H, OCH_3), 5.15 (s, 2H, N-<u>CH2</u>-), 5.79 (1H, s, CH-pyrazole),6.98 (d, J=8.7Hz,2H, H-Ar), 7.63 (d, J=8.7Hz, 2H, H-Ar), 8.14 (1H, s, N=CH), 11.44 (s, 1H, NHCO); ^{13}C NMR: (75MHz, DMSO-d_6, δ (ppm)): 11.60 (CH_3), 13.72 (CH_3), 49.96 (OCH_3), 50.77 (-CH_2), 105.30 (CH, C4-pyrazole), 114.78 (CH, C-Ar), 127.03 (CH, C-Ar), 140.54 (C, C5-pyrazole), 144.32 (CH, N=CH), 147.82 (C, C3-pyrazole), 163.75 (C-OCH_3), 168.77 (C, C=O). ESI-MS: m/z = 287.2 $[M+H]^+$.

(E)-2-(3,5-dimethyl-1H-pyrazol-1-yl)-N'-(4-(dimethylamino)benzylidene)acetohydrazide (**M39**) :

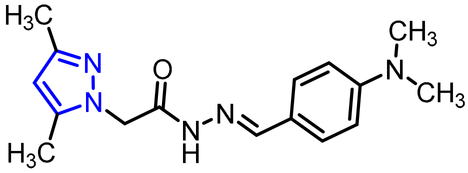

Yield 96% (solid), m.p : 212-214 °C ; FT-IR (ATR, ν(cm^{-1})) : 3192 (NH), 1675 (C=O), 1608 (C=N); ^{1}H NMR: (300MHz, DMSO-d_6, δ(ppm)) : 2.05 (s, 3H, CH_3), 2.12 (s, 3H, CH_3),2.94 (s, 6H, $N(CH_3)_2$), 5.13 (s, 2H, N-<u>CH2</u>-), 5.79 (1H, s, CH-pyrazole), 6.71 (d, J=9.0Hz, 2H, H-Ar), 7.48 (d, J=9.0Hz, 2H, H-Ar), 8.05 (1H, s, N=CH), 11.39 (s, 1H, NHCO); ^{13}C NMR:

(75MHz, DMSO-d_6, δ (ppm)): 11.09 (CH_3), 13.73 (CH_3), 49.94 ($N(CH_3)_2$), 50.84 (-CH_2), 105.15 (CH, C4-pyrazole), 112.25 (CH, C-Ar), 121.73 (C-Ar), 128.66 (CH, C-Ar), 140.53 (C, C5-pyrazole), 144.25(CH, N=CH), 144.45 (C, C3-pyrazole), 152.05 (C-$N(CH_3)_2$), 168.39 (C, C=O). ESI-MS: m/z = 300.01 $[M+H]^+$.

(E)-2-(3,5-dimethyl-1H-pyrazol-1-yl)-N'-(4-nitrobenzylidene)acetohydrazide (**M40**) :

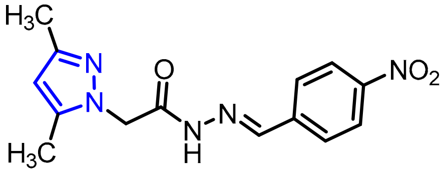

Yield 80% (solid), m.p : 199-201 °C ; FT-IR (ATR, ν(cm^{-1})) : 3393 (NH), 1679 (C=O), 1618 (C=N); ^{1}H NMR: (300MHz, DMSO-d_6, δ(ppm)) : 2.05 (s, 3H, CH_3), 2.13 (s, 3H, CH_3),5.24 (s, 2H, N-<u>CH_2</u>-), 5.81 (1H, s, CH-pyrazole), 7.97 (d, J=9.0Hz, 2H, H-Ar), 8.26 (d, J=9.0Hz, 2H, H-Ar), 8.32 (1H, s, N=CH), 11.91 (s, 1H, NHCO); ^{13}C NMR: (75MHz, DMSO-d_6, δ (ppm)): 11.05 (CH_3), 13.73 (CH_3),50.83 (-CH_2), 105.26 (CH, C4-pyrazole), 124.47 (CH, C-Ar), 128.55 (CH, C-Ar), 140.59 (C-Ar), 144.45 (C, C5-pyrazole), 145.54 (CH, N=CH), 146.78 (C, C3-pyrazole), 148.41 (C-NO_2), 169.50 (C, C=O). ESI-MS: m/z = 302.2 $[M+H]^+$.

(E)-N'-(2-chlorobenzylidene)-2-(3,5-dimethyl-1H-pyrazol-1-yl)acetohydrazide (**M41**) :

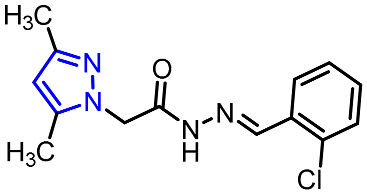

Yield 67% (solid), m.p : 283-285 °C; FT-IR (ATR, ν(cm^{-1})) : 3223 (NH), 1673 (C=O), 1596 (C=N); ^{1}H NMR: (300MHz, DMSO-d_6, δ(ppm)) : 2.05 (s, 3H, CH_3), 2.12 (s, 3H, CH_3),5.20 (s, 2H, N-<u>CH_2</u>-), 5.80 (1H, s, CH-pyrazole), 7.37-7.8.03 (m, 4H, H-Ar), 8.39 (1H, s, N=CH), 11.78 (s, 1H, NHCO); ^{13}C NMR: (75MHz, DMSO-d_6, δ (ppm)): 11.04 (CH_3), 13.73 (CH_3),50.86 (-CH_2), 105.36 (CH, C4-pyrazole), 127.50 (CH, C-Ar), 128.12 (CH, C-Ar), 131.71 (CH, C-Ar),131.87 (CH, C-Ar), 132.10 (C-Cl), 133.44 (C-Ar), 140.56 (C, C5-pyrazole), 143.86 (CH, N=CH), 146.76 (C, C3-pyrazole), 169.23 (C, C=O). ESI-MS: m/z = 290.8 $[M+H]^+$.

(E)-N'-(2,4-dichlorobenzylidene)-2-(3,5-dimethyl-1H-pyrazol-1-yl)acetohydrazide (**M42**) :

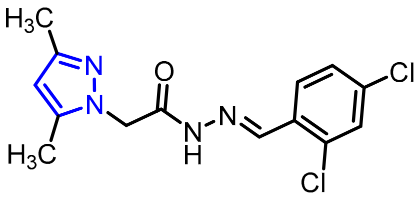

Yield 67% (solid), m.p : 203-205 °C ; FT-IR (ATR, ν(cm^{-1})) : 3413 (NH), 1681 (C=O), 1605 (C=N); ^{1}H NMR: (300MHz, DMSO-d$_6$, δ(ppm)) : 2.05 (s, 3H, CH$_3$), 2.12 (s, 3H, CH$_3$),5.20 (s, 2H, N-CH$_2$-), 5.80 (1H, s, CH-pyrazole), 7.48 (d, J=8.4Hz, 1H, H-Ar), 7.48 (s, 1H, H-Ar), 8.02 (d, J=8.4Hz, 1H, H-Ar), 8.55 (1H, s, N=CH), 11.82 (s, 1H, NHCO); ^{13}C NMR: (75MHz, DMSO-d$_6$, δ (ppm)): 11.03 (CH$_3$), 13.71 (CH$_3$),50.85 (-CH$_2$), 105.39 (CH, C4-pyrazole), 128.53 (CH, C-Ar), 128.73 (C-Cl), 129.82 (CH, C-Ar),130.83 (CH, C-Ar), 134.34 (C-Cl), 135.72 (C-Ar), 140.57 (C, C5-pyrazole), 142.83 (CH, N=CH), 146.80 (C, C3-pyrazole), 169.29 (C, C=O). ESI-MS: m/z = 326.8 [M+H]$^+$.

(E)-N'-(2-bromo-5-hydroxybenzylidene)-2-(3,5-dimethyl-1H-pyrazol-1-yl)acetohydrazide (**M43**) :

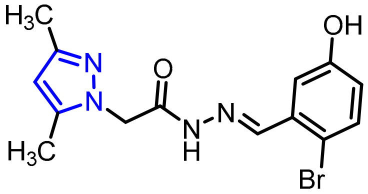

Yield 91% (solid), m.p : 271-273 °C ; FT-IR (ATR, ν(cm^{-1})) : 3153 (NH), 1674 (C=O), 1609 (C=N); ^{1}H NMR: (300MHz, DMSO-d$_6$, δ(ppm)) : 2.05 (s, 3H, CH$_3$), 2.18 (s, 3H, CH$_3$),5.18 (s, 2H, N-CH$_2$-), 5.79 (1H, s, CH-pyrazole), 6.86 (d, J=8.7Hz, 1H, H-Ar), 7.48 (dd, J=8.7Hz, J=2.4Hz, 1H, H-Ar), 7.66 (d, J=2.4Hz, 1H, H-Ar), 8.61 (1H, s, N=CH), 11.38 (s, 1H, OH), 12.04 (s, 1H, NHCO); ^{13}C NMR: (75MHz, DMSO-d$_6$, δ (ppm)): 11.04 (CH$_3$), 13.73 (CH$_3$),50.67 (-CH$_2$), 105.18 (CH, C4-pyrazole), 110.92 (C-Br), 128.73 (CH, C-Ar), 118.87 (CH, C-Ar),121.63 (C-Ar), 128.37 (CH, C-Ar), 134.17 (CH, C-Ar), 140.52 (C, C5-pyrazole), 145.72 (CH, N=CH), 152.02 (C, C3-pyrazole), 164.09 (C-OH), 168.97 (C, C=O). ESI-MS: m/z = 352.1 [M+H]$^+$.

(E)-2-(3,5-dimethyl-1H-pyrazol-1-yl)-N'-(furan-2-ylmethylene)acetohydrazide (**M44**) :

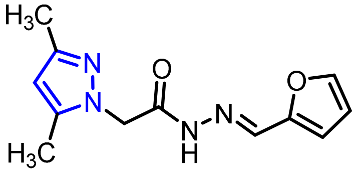

Yield 66% (solid), m.p : 178-180 °C ; FT-IR (ATR, ν(cm^{-1})) : 3211 (NH), 1675 (C=O), 1615 (C=N); ^{1}H NMR: (300MHz, DMSO-d_6, δ(ppm)) : 2.04 (s, 3H, CH_3), 2.12 (s, 3H, CH_3), 5.09 (s, 2H, N-<u>CH_2</u>-), 5.79 (1H, s, CH-pyrazole), 6.59-6.61 (m, 3H, H-Ar), 8.09 (1H, s, N=CH), 11.54 (s, 1H, NHCO); ^{13}C NMR: (75MHz, DMSO-d_6, δ (ppm)): 11.03, 13.72, 50.81, 105.34, 112.63 (C-Br), 114.40, 137.73, 140.61, 145.75, 146.69, 149.58, 168.82. ESI-MS: m/z = 247.1 $[M+H]^+$.

(E)-2-(3,5-dimethyl-1H-pyrazol-1-yl)-N'-(4-methylbenzylidene)acetohydrazide (**M45**) :

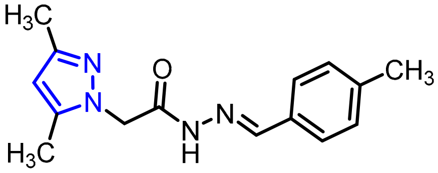

Yield 65% (solid), m.p : 188-190 °C ; FT-IR (ATR, ν(cm^{-1})) : 3425 (NH), 1678 (C=O), 1619 (C=N); ^{1}H NMR: (300MHz, DMSO-d_6, δ(ppm)) : 2.05 (s, 3H, CH_3), 2.12 (s, 3H, CH_3),2.31 (, s, 3H, CH_3), 5.17 (s, 2H, N-<u>CH_2</u>-), 5.79 (1H, s, CH-pyrazole), 7.23 (d, J=7.8Hz, 2H, H-Ar), 7.57 (d, J=7.8Hz, 2H, H-Ar), 8.16 (1H, s, N=CH), 11.52 (s, 1H, NHCO); ^{13}C NMR: (75MHz, DMSO-d_6, δ (ppm)): 11.05 (CH_3), 13.81 (CH_3), 19.94(CH_3), 50.95 (-CH_2), 105.45 (CH, C4-pyrazole), 112.63 (CH, C-Ar), 122.73 (CH, C-Ar), 128.66 (CH, C-Ar), 140.53 (C, C5-pyrazole), 144.75 (C, C3-pyrazole), 146.22 (CH, N=CH), 150.05 (C-CH_3), 169.30 (C, C=O). ESI-MS: m/z = 271.0 $[M+H]^+$.

(E)-N'-(4-chlorobenzylidene)-2-(3,5-dimethyl-1H-pyrazol-1-yl)acetohydrazide (**M46**) :

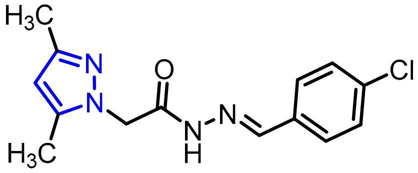

Yield 57% (solid), m.p : 174-176 °C ; FT-IR (ATR, ν(cm^{-1})) : 3397 (NH), 1677 (C=O), 1618 (C=N); ^{1}H NMR: (300MHz, DMSO-d_6, δ(ppm)) : 2.05 (s, 3H, CH_3), 2.12 (s, 3H, CH_3),5.18 (s, 2H, N-<u>CH_2</u>-), 5.80 (1H, s, CH-pyrazole), 7.48 (d, J=8.7Hz, 2H, H-Ar), 7.73 (d, J=8.7Hz, 2H, H-Ar), 8.20 (1H, s, N=CH), 11.67 (s, 1H, NHCO); ^{13}C NMR: (75MHz, DMSO-d_6, δ (ppm)): 11.05 (CH_3), 13.73 (CH_3), 50.81 (-CH_2), 105.33 (CH, C4-pyrazole), 129.06 (CH, C-Ar), 129.33 (CH, C-Ar), 133.48 (C-Ar), 135.09 (C-Cl), 140.55 (C, C5-pyrazole), 143.15 (C, C3-pyrazole), 146.64(CH, N=CH), 169.13 (C, C=O). ESI-MS: m/z = 291.0 $[M+H]^+$.

(E)-2-(3,5-dimethyl-1H-pyrazol-1-yl)-N'-(4-fluorobenzylidene)acetohydrazide (**47**) :

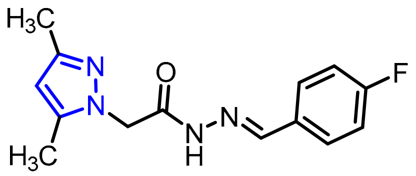

Yield 94% (solid), m.p : 182-184 °C ; FT-IR (ATR, ν(cm^{-1})) : 3336 (NH), 1677 (C=O), 1619 (C=N); ^{1}H NMR: (300MHz, DMSO-d$_6$, δ(ppm)) : 2.05 (s, 3H, CH_3), 2.18 (s, 3H, CH_3),5.18 (s, 2H, N-<u>CH$_2$</u>-), 5.80 (1H, s, CH-pyrazole), 7.24 (d, J=8.7Hz, 2H, H-Ar), 7.78 (d, J=8.7Hz, 2H, H-Ar), 8.00 (1H, s, N=CH), 11.60 (s, 1H, NHCO); ^{13}C NMR: (75MHz, DMSO-d$_6$, δ (ppm)): 11.05 (CH_3), 13.73 (CH_3), 50.80 (-CH_2), 105.20 (CH, C4-pyrazole), 116.74 (CH, C-Ar), 129.85 (CH, C-Ar), 131.05 (C-Ar), 140.54 (C, C5-pyrazole), 143.25 (C, C3-pyrazole), 146.81 (CH, N=CH), 165.14 (C-Cl), 169.05 (C, C=O). ESI-MS: m/z = 275.1 $[M+H]^+$.

(E)-2-(3,5-dimethyl-1H-pyrazol-1-yl)-N'-(4-hydroxy-3-methoxybenzylidene)acetohydrazide (**M48**) :

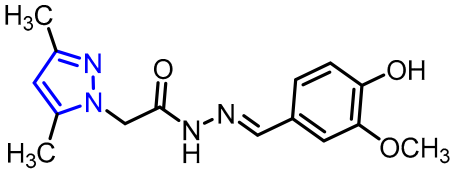

Yiel 69% (solid), m.p : 210-212 °C, FT-IR (ATR, ν(cm^{-1})) : 3215 (NH), 1673 (C=O), 1588 (C=N); ^{1}H NMR: (300MHz, DMSO-d$_6$, δ(ppm)) : 2.05 (s, 3H, CH_3), 2.18 (s, 3H, CH_3),3.80 (s, 3H, OCH_3), 5.17 (s, 2H, N-<u>CH$_2$</u>-), 5.79 (1H, s, CH-pyrazole),6.80 (d, J=8.4Hz, 2H, H-Ar), 7.26 (d, J=8.4Hz, 2H, H-Ar), 7.88 (s, 2H, H-Ar), 8.08 (1H, s, N=CH), 9.50 (1H, s, OH), 11.42 (s, 1H, NHCO); ^{13}C NMR: (75MHz, DMSO-d$_6$, δ (ppm)): 11.09 (CH_3), 13.72 (CH_3), 50.83 (-CH_2), 56.03 (-CH_2), 105.29 (CH, C4-pyrazole), 109.77 (CH, C-Ar), 115.92 (CH, C-Ar), 122.51 (CH, C-Ar), 125.88 (C-Ar), 140.53 (C, C5-pyrazole), 146.62 (C, C3-pyrazole), 148.45 (CH, N=CH), 149.52 (C-OCH_3), 163.64 (C-OH), 168.71 (C, C=O). ESI-MS: m/z = 303.1 $[M+H]^+$.

(E)-N'-(4-bromobenzylidene)-2-(3,5-dimethyl-1H-pyrazol-1-yl)acetohydrazide (**M49**) :

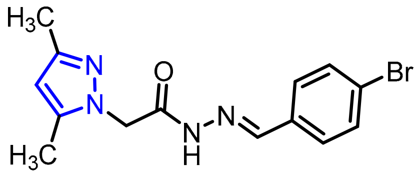

Yield 57% (solid), m.p : 196-198 °C ; FT-IR (ATR, ν(cm^{-1})) : 3296 (NH), 1677 (C=O), 1617 (C=N); ^{1}H NMR: (300MHz, DMSO-d$_6$, δ(ppm)) : 2.05 (s, 3H, CH_3), 2.17 (s, 3H, CH_3),5.18 (s, 2H, N-<u>CH$_2$</u>-), 5.80 (1H, s, CH-pyrazole), 7.61 (d, J=9.0Hz, 2H, H-Ar), 7.66 (d, J=9.0Hz, 2H, H-Ar), 8.18 (1H, s, N=CH), 11.68 (s, 1H, NHCO); ^{13}C NMR: (75MHz, DMSO-d$_6$, δ

(ppm)): 11.05 (CH_3), 13.73 (CH_3), 50.81 ($-CH_2$), 105.33 (CH, C4-pyrazole), 123.65 (C-Br), 129.46 (CH, C-Ar), 133.73 (CH, C-Ar), 133.82 (C-Ar), 140.56 (C, C5-pyrazole), 143.25 (C, C3-pyrazole), 146.72(CH, N=CH), 169.14 (C, C=O). ESI-MS: m/z = 335.2 $[M+H]^+$.

(E)-2-(3,5-dimethyl-1H-pyrazol-1-yl)-N'-(1-phenylethylidene)acetohydrazide (**M50**) :

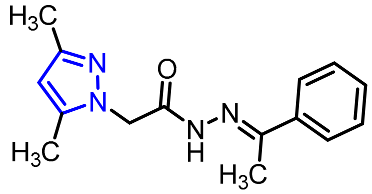

Yield 94% (solid) ; m.p : 155-157 °C ; FT-IR (ATR, ν(cm^{-1})) : 3204 (NH), 1680 (C=O), 1653 (C=N); ^1H NMR: (300MHz, DMSO-d_6, δ(ppm)) : 2.06 (s, 3H,CH_3), 2.18 (s, 3H, CH_3), 5.22 (s, 2H, N-$\underline{CH_2}$-), 5.80 (1H, s, CH-pyrazole), 7.38-7.82 (m, 5H, H-Ar), 10.84 (s, 1H, NHCO); ^{13}C NMR: (75MHz, DMSO-d_6, δ (ppm)): 10.06, 13.74, 14.64, 50.58, 105.22, 126.81, 128.84, 129.65, 138.45, 140.80, 146.68, 149.02, 169.89. ESI-MS: m/z = 271.1 $[M+H]^+$.

4-amino-5-(5-methyl-1H-pyrazol-3-yl)-4H-1,2,4-triazole-3-thiol (**M53**):

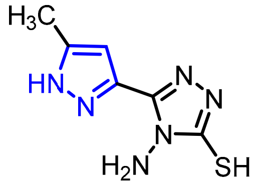

Yield 67% (solid); mp : 216-218 °C; FT-IR (ATR, ν(cm^{-1})): 3144-3367 (NH, NH_2), 2732 (SH); 1H NMR: (300 MHz, DMSO-d6, δ (ppm)): 2.26 (3H, s, CH3), 5.98 (2H, s, NH2), 6.63 (1H, s, CH-pyrazole), 13.10 (1H, s, NH-pz), 13.75 (1H, s, SH); ^{13}C NMR: (75 MHz, DMSO-*d_6*, δ (ppm)): 10.69 (CH3), 105.08 (CH, C4-pyrazole), 138.64 (C, C3-pyrazole), 139.90 (C, C5-pyrazole), 155.40 (C, C5-triazole), 165.06 (C, C-SH). ESI-MS: *m/z* = 197.0 $[M+H]^+$.

4-amino-5-(5-phenyl-1H-pyrazol-3-yl)-4H-1,2,4-triazole-3-thiol (**M54**):

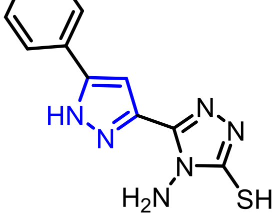

Yield 76% (solid); mp = 287-289 °C; FT-IR (ATR, ν(cm^{-1})): 3133-3277 (NH, NH_2), 2741 (SH); 1H NMR: (300 MHz, DMSO-d6, δ (ppm)): 5.85 (2H, s, NH2), 5.95 (1H, s, CH-pyrazole), 7.28-7.83 (m, 5H, H-Ar), 13.80 (1H, s, NH-pz), 14.05 (1H, s, SH); ^{13}C NMR: (75

MHz, DMSO-d_6, δ (ppm)): 103.57, 128.80, 128.35, 129.14, 133.25, 142.94, 145.07, 151.40, 165.42 (C, C-SH). ESI-MS: *m/z* = 259.0 $[M+H]^+$.

4-amino-5-((3,5-dimethyl-1H-pyrazol-1-yl)methyl)-4H-1,2,4-triazole-3-thiol (**M56**):

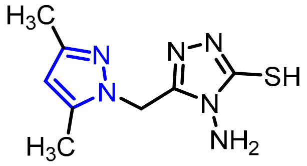

Yield 67 % (solid); m.p: 287-289 °C; FT-IR (ATR, ν(cm^{-1})) : 3188 (NH_2), 2722 (SH); ^{1}H NMR: (300MHz, DMSO-d6, δ(ppm)) : 2.02 (s, 3H, -CH_3), 2.23 (s, 3H, CH_3), 5.19 (s, 2H, N-CH_2-), 5.56 (s, 2H, NH_2), 5.81 (s, 1H, CH-pyrazole); 13.66 (s, 1H, -SH); ^{13}C NMR: (75MHz, DMSO-d6, δ(ppm)) : 11.08 (-CH_3), 13.73 (CH_3), 42.75 (N-CH_2), 105.58 (CH), 140.33 (C); 140.40 (C, C5-pyrazole), 147.22 (C, C5-pyrazole), 148.77 (C, C-SH), 166.78 (CO); ESI-MS: m/z = 225.1 $[M+H]^+$.

(E)-4-((4-(dimethylamino)benzylidene)amino)-5-(5-methyl-1H-pyrazol-3-yl)-4H-1,2,4-triazole-3-thiol (**M59**):

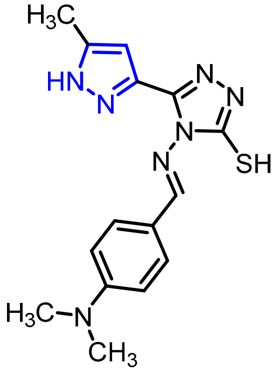

Yield 81% (solid); m.p: 201-203 °C; IR (ν(cm^{-1})): 3143 (NH), 1583 (C=N); ^{1}H NMR: (300MHz, DMSO-d_6, δ(ppm)) : 2.23 (3H, s, CH_3), 3.01 (6H, s, N(CH_3)$_2$, 6.42 (1H, s, CH-pyrazole), 6.79 (2H, d, J = 8.7Hz, Ar-H), 7.69 (2H, d, J =8.7 Hz, Ar-H), 8.96 (1H, s, N=CH), 13.06 (1H, s, NH-pyrazole), 13.90 (1H, s, SH); ^{13}C NMR: (75MHz, DMSO-d_6, δ(ppm)): 10.66 (CH_3), 49.64 (N(CH_3)$_2$, 105.30 (CH, C4-pyrazole), 112.01 (CH, C3-Ar), 119.12 (C, C1-Ar), 131.10 (CH, C2-Ar), 138.49 (C, C5-pyrazole), 139.81 (C, C3-pyrazole), 145.53 (C, C5-triazole), 153.73 (C, C4-Ar), 162.51 (CH, N=C̲H), 168.99 (C, C-SH). ESI-MS: m/z = 328.0 $[M+H]^+$, m/z = 350.5 $[M+Na]^+$.

(E)-4-bromo-3-(((3-mercapto-5-(5-methyl-1H-pyrazol-3-yl)-4H-1,2,4-triazol-4-yl)imino)methyl)phenol (**M63**):

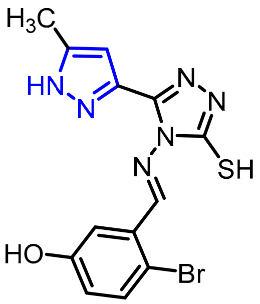

Yield 63% (solid); m.p: 272-274 °C; FT-IR (ATR, ν(cm^{-1})) **:** 3213 (NH), 1571 (C=N); ^{1}H NMR: (300MHz, DMSO-d_6, δ(ppm)) : 2.25 (s, 3H, CH_3), 6.48 (s, 1H, CH-pyrazole), 6.95 (d, *J* = 7.8 Hz, 2H, Ar-H), 7.58 (d, *J* = 7.8 Hz, 2H, Ar-H), 9.83 (s, 1H, OH), 10.92 (1H, s, N=CH), 11.17 (1H, s, NH-pyrazole), 14.09 (1H, s, SH); ^{13}C NMR: (75MHz, DMSO-d_6, δ(ppm)): 10.6, 105.2, 111.1, 119.6, 120.4, 130.7, 135.4, 136.9, 140.1, 144.9, 156.8, 158.0, 162.3. ESI-MS: m/z = 380.4 $[M+H]^+$.

(E)-5-(5-methyl-1H-pyrazol-3-yl)-4-((4-methylbenzylidene)amino)-4H-1,2,4-triazole-3-thiol (**M64**):

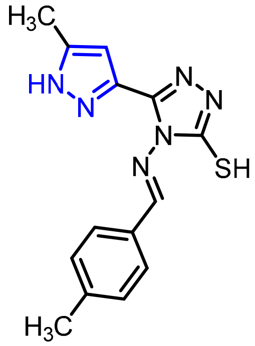

Yield 72% (solid); m.p: 357-359 °C; FT-IR (ATR, ν(cm^{-1})) **:** 3212 (NH), 1572 (C=N); ^{1}H NMR: (300MHz, DMSO-d_6, δ(ppm)) : 2.23 (3H, s, CH_3), 2.38 (3H, s, CH_3), 6.45 (1H, s, CH-pyrazole), 7.36 (2H, d, J = 7.8 Hz, Ar-H), 7.79 (2H, d, J = 7.8 Hz, Ar-H), 9.34 (1H, s, N=CH), 13.08 (1H, s, NH-pyrazole), 14.02 (1H, s, SH); ^{13}C NMR: (75MHz, DMSO-d_6, δ(ppm)): 10.64 (CH_3), 21.74 (CH_3), 105.35 (CH, C4-pyrazole), 129.35 (CH, C3-Ar), 130.23(C, C1-Ar), 138.37 (CH, C2-Ar), 139.96 (C, C5-pyrazole), 143.70 (C, C3-pyrazole), 144.85 (C, C5-triazole), 162.47 (CH, N=C̲H), 168.60 (C, C-SH). ESI-MS: m/z = 299.1 $[M+H]^+$, m/z = 321.1 $[M+Na]^+$.

(E)-4-(((3-mercapto-5-(5-methyl-1H-pyrazol-3-yl)-4H-1,2,4-triazol-4-yl)imino)methyl)-2-methoxyphenol (**M67**):

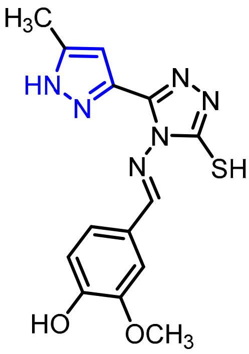

Yield 71% (solid); m.p: 250-252 °C; FT-IR (ATR, ν(cm^{-1})) **:** 3214 (NH), 1573 (C=N); ^{1}H NMR: (300MHz, DMSO-*d6*, δ(ppm)) : 2.23 (3H, s, CH_3), 3.18 (3H, s, OCH_3), 6.45 (1H, s, CH-pyrazole), 6.92 (2H, d, J = 8.1 Hz, Ar-H), 7.33 (2H, d, J = 8.1 Hz, Ar-H), 7.47 (s, 1H, H(Ar), 9.13 (1H, s, N=CH), 10.04 (s, 1H, OH), 13.40 (1H, s, NH-pyrazole), 13.97 (1H, s, SH); ^{13}C NMR: (75MHz, DMSO-d6, δ(ppm)): 10.64, 56.11, 105.37, 111.23, 116.13, 123.75, 125.00, 138.42, 139.93, 144.81, 148.58, 151.98, 162.48, 168.79. ESI-MS: m/z = 331.2 $[M+H]^+$.

(E)-4-(benzylideneamino)-5-(5-phenyl-1H-pyrazol-3-yl)-4H-1,2,4-triazole-3-thiol (**M69**):

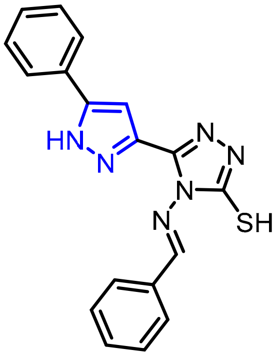

Yield 70% (solid); m.p: 240-242 °C; FT-IR (ATR, ν(cm^{-1})) **:** 3107 (NH), 1596 (C=N); ^{1}H NMR: (300MHz, DMSO-*d6*, δ(ppm)) : 7.16 (1H, s, CH-pyrazole), 6.34-8.76 (m, 10H, Ar-H), 9.78 (1H, s, N=CH), 14.20 (1H, s, NH-pyrazole), 14.40 (1H, s, SH); ^{13}C NMR: (75MHz, DMSO-d6, δ(ppm)): 103.72, 125.78, 129.14, 129.38, 129.58, 129.68, 132.46, 133.38, 139.35, 143.63, 144.51, 162.64, 168.78. ESI-MS: m/z = 347.1 $[M+H]^+$.

(E)-4-((4-(dimethylamino)benzylidene)amino)-5-(5-phenyl-1H-pyrazol-3-yl)-4H-1,2,4-triazole-3-thiol (**M71**):

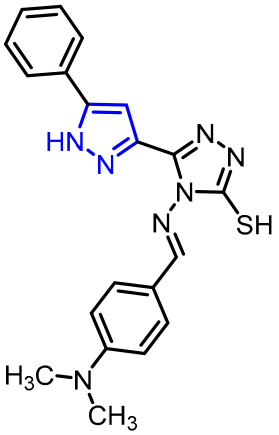

Yield 78% (solid); m.p: 290-292 °C; FT-IR (ATR, ν(cm^{-1})) **:** 3194 (NH), 1599 (C=N); ^1H NMR: (300MHz, DMSO-d_6, δ(ppm)) : 2.98 (s, 6H, $N(CH_3)_2$), 6.72 (1H, s, CH-pyrazole), 6.98 (d, J =8.7Hz, 2H, Ar-H), 7.63 (d, J =8.7Hz, 2H, Ar-H), 7.66-7.83 (m, 5H, H-Ar), 9.38 (1H, s, N=CH), 13.65 (1H, s, NH-pyrazole), 14.06 (1H, s, SH); ^{13}C NMR: (75MHz, DMSO-d_6, δ(ppm)): 46.0, 103.89, 112.55, 122.77, 126.01, 128.22, 128.95, 129.32, 131.12, 134.35, 148.62, 151.31, 152.90, 155.43, 160.13. ESI-MS: m/z = 390.0 $[M+H]^+$.

(E)-4-((2-chlorobenzylidene)amino)-5-(5-phenyl-1H-pyrazol-3-yl)-4H-1,2,4-triazole-3-thiol (**M72**):

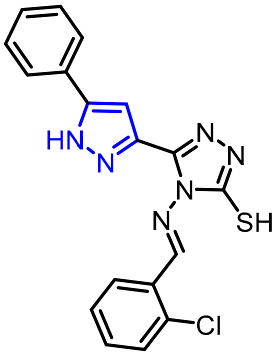

Yield 89% (solid), m.p: 312-314 °C; FT-IR (ATR, ν(cm^{-1})) **:** 3118 (NH), 1593 (C=N); ^1H NMR: (300MHz, DMSO-d_6, δ(ppm)) : 7.20 (1H, s, CH-pyrazole), 7.34-8.20 (m, 9H, H-Ar), 10.18 (1H, s, N=CH), 13.95 (1H, s, NH-pyrazole), 14.24 (1H, s, SH); ^{13}C NMR: (75MHz, DMSO-d_6, δ(ppm)): 104.07, 125.78, 128.52, 128.86, 129.14, 129.62, 130.17, 130.84, 134.64, 135.70, 139.28, 143.63, 144.92, 162.10 ; 162.44. ESI-MS: m/z = 381.0 $[M+H]^+$.

(E)-4-((2,4-dichlorobenzylidene)amino)-5-(5-phenyl-1H-pyrazol-3-yl)-4H-1,2,4-triazole-3-thiol (**M73**):

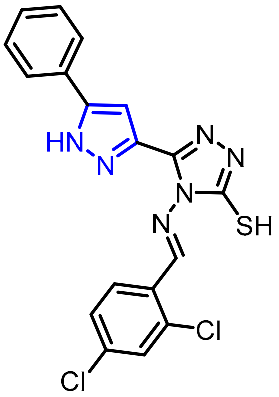

Yield 62% (solid); m.p: 317-319 °C; FT-IR (ATR, ν(cm^{-1})) **:** 3111 (NH), 1589 (C=N); ^{1}H NMR: (300MHz, DMSO-*d*$_6$, δ(ppm)) : 7.20 (1H, s, CH-pyrazole), 7.34 (d, J =8.7Hz, 1H, Ar-H), 7.42-7.93 (m, 5H, H-Ar), 8.17 (d, J =8.7Hz, 1H, Ar-H), 8.75 (s, 1H, Ar-H), 10.24 (1H, s, N=CH), 13.99 (1H, s, NH-pyrazole), 14.08 (1H, s, SH); ^{13}C NMR: (75MHz, DMSO-d$_6$, δ(ppm)): 104.33, 125.83, 128.94, 129.10, 129.27, 129.33, 129.42, 129.51, 129.65, 130.37, 136.51, 138.48, 145.35, 153.23, 160.98, 162.38. ESI-MS: m/z = 415.1 [M+H]$^+$.

(E)-4-bromo-3-(((3-mercapto-5-(5-phenyl-1H-pyrazol-3-yl)-4H-1,2,4-triazol-4-yl)imino)methyl)phenol (**M74**):

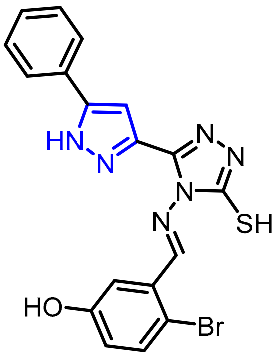

Yield 91% (solid); m.p: 271-273 °C; FT-IR (ATR, ν(cm^{-1})) **:** 3220 (NH), 1599 (C=N); ^{1}H NMR: (300MHz, DMSO-*d*$_6$, δ(ppm)) : 6.91 (1H, s, CH-pyrazole), 7.34-7.90 (m, 8H, H-Ar), 8.90 (1H, s, N=CH), 13.93 (1H, s, NH-pyrazole), 14.19 (1H, s, SH); ^{13}C NMR: (75MHz, DMSO-d$_6$, δ(ppm)): 103.86, 111.04, 119.38, 120.61, 125.79, 128.78, 129.17, 130.71, 132.09, 135.96, 136.97, 144.51, 153.45, 158.11, 161.29, 162.49. ESI-MS: m/z = 441.1 [M+H]$^+$.

(E)-4-((furan-2-ylmethylene)amino)-5-(5-phenyl-1H-pyrazol-3-yl)-4H-1,2,4-triazole-3-thiol (**M75**):

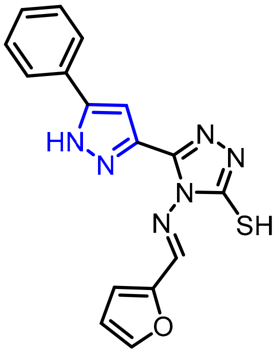

Yield 69% (solid); m.p: 326-328 °C; FT-IR (ATR, ν(cm^{-1})) **:** 3120 (NH), 1612 (C=N); ^{1}H NMR: (300MHz, DMSO-*d6*, δ(ppm)) : 6.78 (1H, s, CH-pyrazole), 7.16-8.07 (m, 8H, H-Ar), 9.35 (1H, s, N=CH), 13.02 (1H, s, NH-pyrazole), 13.98 (1H, s, SH); ^{13}C NMR: (75MHz, DMSO-d6, δ(ppm)): 103.53, 113.53, 121.03, 125.75, 125.95, 129.13, 129.61, 132.50, 142.12, 143.55, 147.75, 148.66, 156.34, 162.59. ESI-MS: m/z = 337.1 [M+H]$^{+}$.

(E)-4-((4-methylbenzylidene)amino)-5-(5-phenyl-1H-pyrazol-3-yl)-4H-1,2,4-triazole-3-thiol (**M76**):

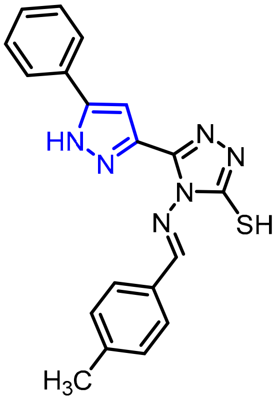

Yield 44% (solid); m.p: 339-341 °C; FT-IR (ATR, ν(cm^{-1})) **:** 3207 (NH), 1561 (C=N); ^{1}H NMR: (300MHz, DMSO-*d6*, δ(ppm)) : 3.54 (s, 3H, CH_3), 6.92 (1H, s, CH-pyrazole), 7.25 (d, J =8.1Hz, 2H, Ar-H), 7.36-7.46 (m, 5H, H-Ar), 7.82 (d, J =8.1Hz, 2H, Ar-H), 9.45 (1H, s, N=CH), 12.98 (1H, s, NH-pyrazole), 13.65 (1H, s, SH); ^{13}C NMR: (75MHz, DMSO-d6, δ(ppm)): 21.65, 103.70, 126.80, 127.56, 128.54, 128.91, 129.05, 129.48, 132.19, 134.15, 140.33, 148.26, 152.90, 150.92, 155.11, 161.03. ESI-MS: m/z = 361.0 [M+H]$^{+}$.

(E)-4-((4-chlorobenzylidene)amino)-5-(5-phenyl-1H-pyrazol-3-yl)-4H-1,2,4-triazole-3-thiol (**M77**):

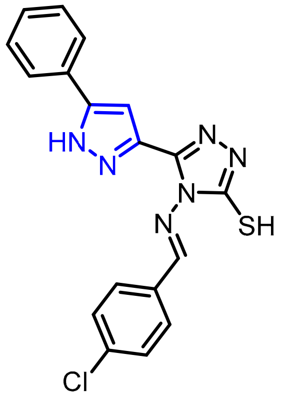

Yield 40% (solid); m.p: 319-321 °C; FT-IR (ATR, ν(cm^{-1})) **:** 3114 (NH), 1588 (C=N); ^{1}H NMR: (300MHz, DMSO-*d$_6$*, δ(ppm)) : 7.16 (1H, s, CH-pyrazole), 7.18-7.41 (m, 5H, H-Ar), 7.77 (d, J =8.1Hz, 2H, Ar-H), 7.95 (d, J =8.1Hz, 2H, Ar-H), 9.53 (1H, s, N=CH), 13.93 (1H, s, NH-pyrazole), 14.19 (1H, s, SH); ^{13}C NMR: (75MHz, DMSO-d$_6$, δ(ppm)): 103.74, 125.79, 128.40, 128.81, 129.55, 130.49, 131.40, 133.10, 138.02, 139.33, 144.51, 151.61, 162.68, 167.36. ESI-MS: m/z = 381.1 [M+H]$^+$.

(E)-4-((4-fluorobenzylidene)amino)-5-(5-phenyl-1H-pyrazol-3-yl)-4H-1,2,4-triazole-3-thiol (**M78**):

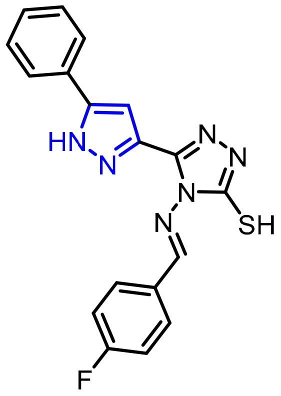

Yield 59% (solid); m.p: 301-303 °C; FT-IR (ATR, ν(cm^{-1})) **:** 3165 (NH), 1558 (C=N); ^{1}H NMR: (300MHz, DMSO-*d$_6$*, δ(ppm)) : 7.21 (1H, s, CH-pyrazole), 7.38-8.03 (m, 9H, H-Ar), 9.27 (1H, s, N=CH), 13.23 (1H, s, NH-pyrazole), 14.15 (1H, s, SH); ^{13}C NMR: (75MHz, DMSO-d$_6$, δ(ppm)): 103.16, 123.13, 125.44, 129.39, 129.51, 129.73, 129.92, 132.20, 134.15, 146.83, 152.03, 155.88, 162.23, 164.40. ESI-MS: m/z = 365.1 [M+H]$^+$.

(E)-4-((4-bromobenzylidene)amino)-5-(5-phenyl-1H-pyrazol-3-yl)-4H-1,2,4-triazole-3-thiol (**M79**):

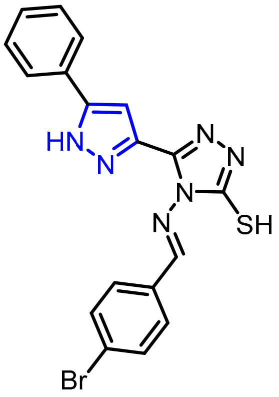

Yield 56% (solid); m.p: 285-287 °C; FT-IR (ATR, ν(cm^{-1})) **:** 3133 (NH), 1590 (C=N); ^{1}H NMR: (300MHz, DMSO-*d$_6$*, δ(ppm)) : 7.16 (1H, s, CH-pyrazole), 7.34-7.48 (m, 5H, H-Ar), 7.77 (d, J =8.4Hz, 2H, Ar-H), 7.88 (d, J =8.4Hz, 2H, Ar-H), 9.52 (1H, s, N=CH), 13.90 (1H, s, NH-pyrazole), 14.18 (1H, s, SH); ^{13}C NMR: (75MHz, DMSO-d$_6$, δ(ppm)): 103.75, 125.79, 127.08, 128.81, 129.15, 129.62, 131.14, 131.72, 132.18, 134.31, 145.53, 150.80, 162.65, 167.50. ESI-MS: m/z = 426.2 [M+H]$^+$.

5-(5-methyl-1H-pyrazol-3-yl)-4-phenyl-2,4-dihydro-3H-1,2,4-triazol-3-one (**M82**):

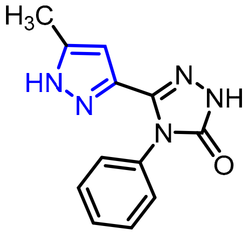

Yield 81% (solid); m.p: 368-370 °C; FT-IR (ATR, ν(cm^{-1})) **:** 3168-3406 (NH), 1655 (C=O); ^{1}H NMR: (300MHz, DMSO-*d$_6$*, δ(ppm)) : 2.13 (s, 3H, CH_3), 6.06 (1H, s, CH-pyrazole), 7.23-7.42 (m, 5H, H-Ar), 12.06 (s, 1H, NH-triazole), 12.79 (s, 1H, NH-pyrazole); ^{13}C NMR: (75MHz, DMSO-d$_6$, δ(ppm)): 10.58, 104.06, 128.47, 128.77, 129.28, 134.50, 139.44, 142.52, 146.02, 154.97. ESI-MS: m/z = 258.2 [M+H]$^+$.

4-phenyl-5-(5-phenyl-1H-pyrazol-3-yl)-2,4-dihydro-3H-1,2,4-triazol-3-one (**M83**):

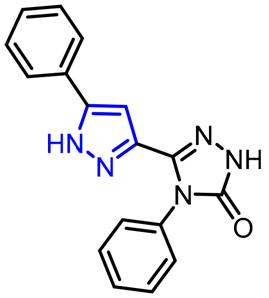

Yield 82% (solid); m.p: 277-279 °C; FT-IR (ATR, ν(cm^{-1})) **:** 3169-3389 (NH), 1655 (C=O); ^{1}H NMR: (300MHz, DMSO-*d6*, δ(ppm)) : 6.66 (1H, s, CH-pyrazole), 7.29-7.6 (m, 5H, H-Ar), 11.98 (s, 1H, NH-triazole), 12.66 (s, 1H, NH-pyrazole); ESI-MS: m/z = 304.1 [M+H]$^+$.

5-(5-methyl-1H-pyrazol-3-yl)-1,3,4-oxadiazole-2-thiol (**M86**):

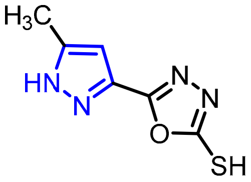

Yield 68% (solid); m.p: 352-354 °C; FT-IR (ATR, ν(cm^{-1})) **:** 3220 (NH), 2720 (SH), 1637 (C=O); ^{1}H NMR: (300MHz, DMSO-*d6*, δ(ppm)) : 2.27 (s, 3H, CH_3), 6.54 (1H, s, CH-pyrazole), 13.34 (s, 1H, NH-pyrazole), 14.62 (s, 1H, SH) ; ^{13}C NMR: (75MHz, DMSO-d6, δ(ppm)): 14.02, 104.14, 134.16, 149.91, 155.48, 179.26. ESI-MS: m/z = 183.2 [M+H]$^+$.

References

[1]. K. Karrouchi, S. Fettach, S. Radi, E. bekkaye Yousfi, J. Taoufik, Y.N. Mabkhot, S. Alterary, M.E.A.F. and M. Ansar, Synthesis, Characterization, Free-radical Scavenging Capacity and Antioxidant Activity of Novel Series of Hydrazone, 1,3,4-oxadiazole and 1,2,4- triazole Derived from 3,5-dimethyl-1H-pyrazole, Lett. Drug Des. Discov. 16 (2019) 712–720. https://doi.org/http://dx.doi.org/10.2174/1570180815666180516103050.

[2]. K. Karrouchi, L. Chemlal, J. Taoufik, Y. Cherrah, S. Radi, M. El Abbes Faouzi, M. Ansar, Synthesis, antioxidant and analgesic activities of Schiff bases of 4-amino-1,2,4-triazole derivatives containing a pyrazole moiety, Ann. Pharm. Françaises. 74 (2016) 431–438. https://doi.org/https://doi.org/10.1016/j.pharma.2016.03.005.

[3]. R.R. Pillai, K. Karrouchi, S. Fettach, S. Armaković, S.J. Armaković, Y. Brik, J. Taoufik, S. Radi, M. El Abbes Faouzi, M. Ansar, Synthesis, spectroscopic characterization, reactive properties by DFT calculations, molecular dynamics simulations and biological evaluation of Schiff bases tethered 1,2,4-triazole and pyrazole rings, J. Mol. Struct. 1177 (2019) 47–54. https://doi.org/https://doi.org/10.1016/j.molstruc.2018.09.037.

[4]. K. Karrouchi, E.B. Yousfi, N.K. Sebbar, Y. Ramli, J. Taoufik, Y. Ouzidan, M. Ansar, Y.N. Mabkhot, H.A. Ghabbour, S. Radi, New Pyrazole-Hydrazone Derivatives: X-ray Analysis, Molecular Structure Investigation via Density Functional Theory (DFT) and

Their High In-Situ Catecholase Activity, Int. J. Mol. Sci. 18 (2017). https://doi.org/10.3390/ijms18112215.

[5]. K. Karrouchi, S.A. Brandán, Y. Sert, H. El-marzouqi, S. Radi, M. Ferbinteanu, M.E.A. Faouzi, Y. Garcia, M. Ansar, Synthesis, X-ray structure, vibrational spectroscopy, DFT, biological evaluation and molecular docking studies of (E)-N'-(4-(dimethylamino)benzylidene)-5-methyl-1H-pyrazole-3-carbohydrazide, J. Mol. Struct. 1219 (2020) 128541. https://doi.org/https://doi.org/10.1016/j.molstruc.2020.128541.

[6]. K. Karrouchi, S.A. Brandán, Y. Sert, M. El Karbane, S. Radi, M. Ferbinteanu, Y. Garcia, M. Ansar, Synthesis, structural, molecular docking and spectroscopic studies of (E)-N'-(4-methoxybenzylidene)-5-methyl-1H-pyrazole-3-carbohydrazide, J. Mol. Struct. 1225 (2021) 129072. https://doi.org/https://doi.org/10.1016/j.molstruc.2020.129072.

[7]. K. Karrouchi, S.A. Brandán, M. Hassan, K. Bougrin, S. Radi, M. Ferbinteanu, Y. Garcia, M. Ansar, Synthesis, X-ray, spectroscopy, molecular docking and DFT calculations of (E)-N'-(2,4-dichlorobenzylidene)-5-phenyl-1H-pyrazole-3-carbohydrazide, J. Mol. Struct. 1228 (2021) 129714. https://doi.org/https://doi.org/10.1016/j.molstruc.2020.129714.

[8]. K. Karrouchi, M. Ansar, S. Radi, M. Saadi, L. El Ammari. Crystal structure of N′-diphenylmethylidene-5-methyl-1H-pyrazole-3-carbohydrazide. Acta Cryst E. 71 (2015) o890-o891. doi: 10.1107/S2056989015020071.

[9]. K. Karrouchi, S. Radi, J. Taoufik, H. A. Ghabbour, Y. N. Mabkhot. Crystal structure of N′-(4-(dimethylamino) benzylidene)-5-phenyl-1H-pyrazole-3-carbohydrazide, C19H19N5O. Z. Kristallogr. NCS. 231 (2016) 883-886. https://doi.org/10.1515/ncrs-2015-0303.

[10]. K. Karrouchi, S. Radi, J. Taoufik, H. A. Ghabbour, Y. N. Mabkhot. Crystal structure of N′-(4-methoxybenzylidene)-5-phenyl-1H-pyrazole-3-carbohydrazide, C18H16N4O2. Z. Kristallogr. NCS. 231 (2016) 835-837. https://doi.org/10.1515/ncrs-2015-0286.

[11]. K. Karrouchi, S. Radi, J. Taoufik, H. A. Ghabbour, Y. N. Mabkhot. Crystal structure of N′-(4-nitrobenzylidene)-5-phenyl-1H-pyrazole-3-carbohydrazide, C17H13N5O3. Z. Kristallogr. NCS. 231 (2016) 839-841. https://doi.org/10.1515/ncrs-2015-0287.

[12]. Karrouchi, K., Fettach, S., Tüzün, B., Radi, S., Alharthi, A. I., Ghabbour, H. A., Mabkhot, Y.N, Faouzi, M.E.A., Ansar, M., Garcia, Y. (2021). Synthesis, crystal structure, DFT, α-glucosidase and α-amylase inhibition and molecular docking studies

of (E)-N'-(4-chlorobenzylidene)-5-phenyl-1H-pyrazole-3-carbohydrazide, J. Mol. Struct., 1245 (2021)131067. https://doi.org/10.1016/j.molstruc.2021.131067.
